# Statistical modeling of gut microbiota for personalized health status monitoring

**DOI:** 10.1186/s40168-023-01614-x

**Published:** 2023-08-18

**Authors:** Jinlin Zhu, Heqiang Xie, Zixin Yang, Jing Chen, Jialin Yin, Peijun Tian, Hongchao Wang, Jianxin Zhao, Hao Zhang, Wenwei Lu, Wei Chen

**Affiliations:** 1 State Key Laboratory of Food Science and Resources, Jiangnan University, Wuxi, Jiangsu 214122 China; 2 School of Food Science and Technology, Jiangnan University, Wuxi, Jiangsu 214122 China; 3 (Yangzhou) Institute of Food Biotechnology, Jiangnan University, Yangzhou, Jiangsu 225004 China; 4 National Engineering Research Center for Functional Food, Jiangnan University, Wuxi, Jiangsu 214122 China; 5 Wuxi Translational Medicine Research Center, Jiangsu Translational Medicine Research Institute Wuxi Branch, Wuxi, Jiangsu China; 6 International Joint Research Laboratory for Pharmabiotics & Antibiotic Resistance, Jiangnan University, Wuxi, Jiangsu 214122 China

**Keywords:** Personalized health prediction, Gut microbiome, Principal component analysis, Statistical inference, Machine learning

## Abstract

**Background:**

The gut microbiome is closely associated with health status, and any microbiota dysbiosis could considerably impact the host’s health. In addition, many active consortium projects have generated many reference datasets available for large-scale retrospective research. However, a comprehensive monitoring framework that analyzes health status and quantitatively present bacteria-to-health contribution has not been thoroughly investigated.

**Methods:**

We systematically developed a statistical monitoring diagram for personalized health status prediction and analysis. Our framework comprises three elements: (1) a statistical monitoring model was established, the health index was constructed, and the health boundary was defined; (2) healthy patterns were identified among healthy people and analyzed using contrast learning; (3) the contribution of each bacterium to the health index of the diseased population was analyzed. Furthermore, we investigated disease proximity using the contribution spectrum and discovered multiple multi-disease-related targets.

**Results:**

We demonstrated and evaluated the effectiveness of the proposed monitoring framework for tracking personalized health status through comprehensive real-data analysis using the multi-study cohort and another validation cohort. A statistical monitoring model was developed based on 92 microbial taxa. In both the discovery and validation sets, our approach achieved balanced accuracies of 0.7132 and 0.7026, and AUC of 0.80 and 0.76, respectively. Four health patterns were identified in healthy populations, highlighting variations in species composition and metabolic function across these patterns. Furthermore, a reasonable correlation was found between the proposed health index and host physiological indicators, diversity, and functional redundancy. The health index significantly correlated with Shannon diversity ($\rho = 0.07$) and species richness ($\rho = 0.44$) in the healthy samples. However, in samples from individuals with diseases, the health index significantly correlated with age ($\rho = 0.12$), species richness ($\rho = 0.46$), and functional redundancy ($\rho = - 0.16$). Personalized diagnosis is achieved by analyzing the contribution of each bacterium to the health index. We identified high-contribution species shared across multiple diseases by analyzing the contribution spectrum of these diseases.

**Conclusions:**

Our research revealed that the proposed monitoring framework could promote a deep understanding of healthy microbiomes and unhealthy variations and served as a bridge toward individualized therapy target discovery and precise modulation.

Video Abstract

**Supplementary Information:**

The online version contains supplementary material available at 10.1186/s40168-023-01614-x.

## Introduction

Gut microbiota is a large community of microorganisms in the human gastrointestinal tract. Deciphering the role of such vital organs to one’s health has drawn great interest within the health research community. After decade-long research, there is now a global consensus that these microbes are significant to human health [1], as they replace many functional aspects of the host, and any dysbiosis of microbiota could largely influence the host’s immune, metabolic, and even neurobehaviors [2]. Moreover, many active consortium projects have significantly contributed to the extensive profiling of massive data and understanding of individual health, making available many reference datasets for large-scale retrospective research [3–5]. Therefore, highly automated and powerful bioinformatics tools for personalized health status inference are expected to translate the composition of the human microbiome into useful clinical indications for non-invasive wellness monitoring, diagnosis, and treatment [6, 7].

Typically, these high-throughput raw sequencing data reads are clustered and organized into operational taxonomic units for downstream analyses [8], which is usually a high-dimensional matrix with large variability and great sparsity [9]. A statistical monitoring panel is imperative in population-level health analysis and disease-associated signature exploration to distill advisable knowledge and intelligence from the compositional table and to promote timely health warnings. In microbiome literature, principal component analysis (PCA) is the most widely adopted statistical method [10–13]. PCA allows feature extraction and knowledge representation by deconstructing variation or correlations among samples as a simple and effective model for data inspection, interpretation, and utilization. In this view, the high-dimensional composition is significantly reduced, and an elegant ordination visualization can be presented for differentiation judgment among sample groups. However, studies in this area are limited to qualitative assessment and lack quantitative contextualization disentanglement, which in turn impacts the utility ranges for phenotype parsing and health understanding. A pioneering work recently proposed the gut microbiota health index (GMHI) for differentiating healthy from nonhealthy populations [6]. GMHI was formulated on 50 species containing both health-prevalent and health-scarce species, and the method could distinguish between healthy and unhealthy individuals with relatively high balanced accuracy. Although GMHI has achieved some success, it has a few limitations. First, the method was designed to distinguish healthy from unhealthy individuals and could potentially overestimate a patient’s health literacy. This could prove deceptive in clinical applications, as missing alarms could be disastrous for accurate disease diagnosis. Second, the model deployed on the collective abundance index cannot trace back to those most responsible species associated with the reported phenotype. These drawbacks restricted the model interpretation for further personalized medication. More recently, a microbiome risk score (MRS) method was induced by the alpha diversity of the identified candidate taxa [14]. Likewise, it generally reported comparable accuracies with GMHI on the selected community taxa but still lacked model interpretation and could hardly support personalized health analysis.

**Fig. 1:**
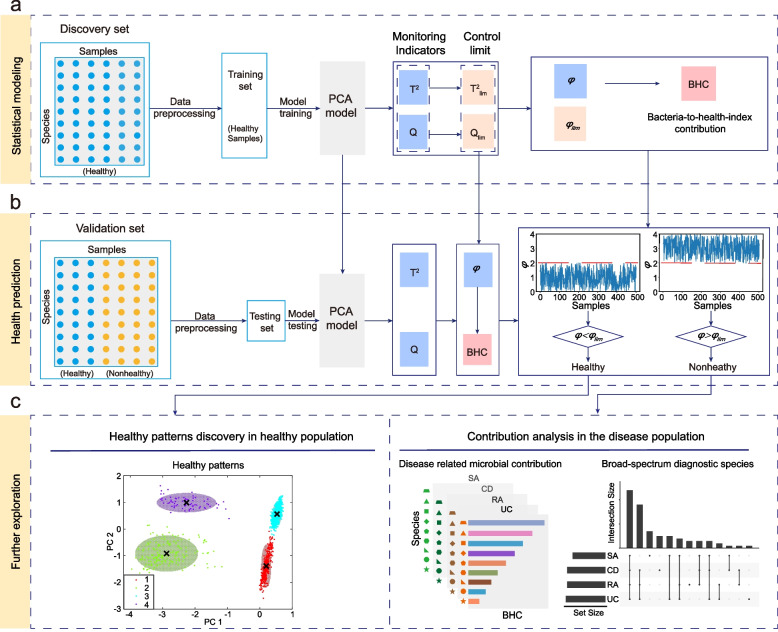
The workflow of personalized health status monitoring framework.** a** Statistical modeling: Healthy samples from the discovery set are subjected to data preprocessing and subsequently used as a training set to train the model. The model establishes quantitatively a computational pipeline to infer the health index ($\phi$) and the BHC.** b** Health prediction: The health index and related threshold output from the PCA model are used to predict the health of the samples. **c** Further exploration: Health patterns discovery in the healthy population through contrastive PCA learning. In disease populations, the target diagnostic species and the contribution of species, determined by contribution analysis, identify broad-spectrum diagnostic species

In this study, we defined a novel and systematic monitoring flowchart for gut microbiota health prediction and disease analysis, the workflow of which is shown in Fig. 1. Our primary aim was to define a rational health index with a health boundary to make further inferences on various nonhealthy cases. As healthy samples are usually far more numerous than those of specific human diseases, a statistical monitoring framework should become viable to acquire such a boundary. To this end, several questions should be answered: (1) How to convert correlations among a set of core species into a robust health index panel and select core species that offer a better and more balanced prediction? (2) How to determine potential healthy microbial patterns given the health boundary? (3) How to judge the rationality of the health index in respect of species community properties and host physiological measures? (4) How to identify the microorganisms that mainly contribute to different human diseases in unhealthy samples? (5) Are there any broad-spectrum contributing species across the various disease phenotypes? Based on these explorations, we can employ the globalized population to conduct quantitative and qualitative research, from the macro-ecology investigation of health pattern discovery to the micro-ecology evaluation of personalized health status analysis. In a broader context, the entire working pipeline will greatly extend the merits of statistical inference in conventional microbiome study, not only for primitive data visualization but also for deeper data understanding with nonhealthy detection and reasoning, which paves the way for a valuable prototype of the global microbiome research community.

## Methods

### Multi-study integration and metagenomic upstream analysis of human stool metagenomes

The 4347 samples (discovery cohort) and the other 782 samples (validation cohort) were all extracted from publicly available research [6]. The discovery cohort was collected from 34 studies, including healthy and 12 unhealthy phenotypes. The validation cohort comprised 15 sub-cohorts across 11 healthy and nonhealthy phenotypes from nine studies. In both cohorts, subjects with various diseases were pooled into an unhealthy group, and the rest were reported as healthy in the healthy group. The detailed description of health in each previous article is shown in Table S1. The additional test cohort was derived from five independent studies containing 605 samples. After integration, reclassification, and quality control, 2636 samples were considered healthy and 1711 unhealthy in the discovery cohort. There were 118 healthy and 664 unhealthy samples in the validation cohort, and test cohort included 292 healthy and 313 unhealthy samples.

The species identification and abundance calculation of metagenomic cohorts were consistent with the previous study [6]. Sequence read of stool samples using KneadData v0.5.1 quality control pipe for processing. The metagenomic reads were then classified into species-level categories using MetaPhlAn2 and a database of clade-specific marker genes obtained from approximately 17,000 microbial genomes (mpa_v20_m200). Species were removed based on taxonomic profiles, and the species table was subsequently obtained.

### Data preprocessing

Data preprocessing included feature selection, data transformation, and normalization. Feature selection attempts were made to identify principal microbial species by reducing the number of unimportant features, with the expectation of reducing the computational cost and improving the predictive model’s performance. In the GMHI study, the prevalence-based (PR) strategy was used, and health-prevalent/health-scarce species were determined by investigating the optimal classification performance. In this study, besides the PR strategy, we further considered other widely used methods, including machine learning classifiers, such as random forest (RF), eXtreme Gradient Boosting (XGB), and correlation analysis methods like Spearman’s correlation (SPC) and maximum information coefficient (MIC). The hypothesis test method in healthy and unhealthy groups has been deployed as the third category. For classifiers, species were ranked according to the important values, and those with high importance were considered for PCA modeling. For correlation-based methods, species are ranked and selected based on the absolute values of correlations. The Kolmogorov-Smirnov test was used for the hypothesis test method to identify those health-prevalent and health-scarce features. Only species features with *P*-values under 0.001 were considered. Transformation is first required to perform a reasonable analysis using PCA. In this study, we considered the relative abundances. The values may range from 0 to large real values, and most of the magnitudes range from 10^−1^ to 10^−3^. The following logarithmic transformation was designed and applied since low-abundance species may play important roles in health status. When $x < = 1$, we use $lt(x) = \log_{2} (2x + \sigma )$; when $x > 1$, we use $lt(x) = \sqrt x$ to process the data.

A small $\sigma$ is added to avoid numerical issues at the origin. Once the data has been transformed, the *z*-score normalization is engaged to adjust the mean to 0 and the standard deviation to 1.

### Health index with PCA (hiPCA)

Microbiome data is usually large and difficult to interpret. Thus, PCA is widely used to drastically reduce the high dimensionality so that the maximal variability (i.e., statistical information) in the data can be preserved. To achieve this, PCA is translated into the eigenvalue/eigenvector problem in the standard context, based on which the eigenvalues of the covariance or correlation matrix are rearranged after singular value decomposition (SVD). Since the eigenvalues imply the variances defined by the corresponding eigenvector, the principal component variables can be selected based on $k$ largest eigenvalues, and the rest of the variations are set apart as residuals or noises. In general, assuming that the matrix ${\mathbf{X}} \in R^{N \times D}$ consists of $N$ records by $D$ microbial features, the PCA model structure is given as follows

1 $${\mathbf{X}} = {\mathbf{TP}}^{T} + {\mathbf{E}}$$

where ${\mathbf{T}} \in R^{N \times d}$ is the score matrix, ${\mathbf{P}} \in R^{D \times d}$ is the loading matrix, $d$ is the retained latent dimensionality, and ${\mathbf{E}}$ is the residual matrix. Technically, if we consider the covariance as the example, by performing the Eigen-decomposition of the covariance matrix ${{{\mathbf{S}} = ({\mathbf{X}}^{T} {\mathbf{X}})} \mathord{\left/ {\vphantom {{{\mathbf{S}} = ({\mathbf{X}}^{T} {\mathbf{X}})} {(N - 1}}} \right. \kern-0pt} {(N - 1}})$, we get

2 $${\mathbf{S}} = \left[ {\begin{array}{*{20}c} {\mathbf{P}} & {{\tilde{\mathbf{P}}}} \\ \end{array} } \right]\left[ {\begin{array}{*{20}c} {{\varvec{\Lambda}}} & {\mathbf{0}} \\ {\mathbf{0}} & {{\tilde{\mathbf{\Lambda }}}} \\ \end{array} } \right]\left[ {\begin{array}{*{20}c} {\mathbf{P}} & {{\tilde{\mathbf{P}}}} \\ \end{array} } \right]^{T}$$

where ${\tilde{\mathbf{P}}}$ is the residual loading, ${{\varvec{\Lambda}}}$ and ${\tilde{\mathbf{\Lambda }}}$ are eigenvalues for latent and residual subspaces, respectively. Accordingly, the principal component subspace (PCS) and residual subspace (RS) for data can be defined as

3 $${\hat{\mathbf{X}}} = {\mathbf{XPP}}^{T} = {\mathbf{XC}},$$

4 $$\widetilde{\mathbf X}={\mathbf X\overset{\boldsymbol\sim}{\mathbf P}\overset{\boldsymbol\sim}{\mathbf P}}^T=\mathbf X\overset{\boldsymbol\sim}{\mathbf C},$$

where ${\mathbf{C}}$ and ${\tilde{\mathbf{C}}}$ are projection matrices to latent and residual subspaces, respectively. We can calculate the cumulative percentage sum of explained variances to determine the right number of principal components (PCs). Assuming that the eigenvalues are arranged in descending order as $\lambda_{1} ,\lambda_{2} , \ldots ,\lambda_{D}$, the percentage of explained variances (PEVs) for each eigenvalue is defined as ${{\lambda_{i} } \mathord{\left/ {\vphantom {{\lambda_{i} } {\sum\nolimits_{i}^{{}} {\lambda_{i} } }}} \right. \kern-0pt} {\sum\nolimits_{i}^{{}} {\lambda_{i} } }}$. The right number of PCs can be determined by accumulating the PEVs until the total variance is satisfactory for the research.

The main idea of the health index was to quantitatively determine a health boundary based on the explanatory model. This was similar to statistical process control, where the control charts were used to display measurements of process samples over time. In contrast, our microbiome study considered the health index chart that evaluated the gut microbiota composition samples over population. Specifically, three charts were designed, namely Hoteling’s $T^{2}$ chart, the Q chart, and the combined chart $\phi$ [15, 16] to reflect the degree of deviation from health.

#### Hoteling’s $T^{2}$ index

Given a composition sample ${\mathbf{x}}$, the $T^{2}$ index monitors the PC subspace defined as $T^{2} \left( {\mathbf{x}} \right) = {\mathbf{x}}^{T} {\mathbf{Dx}}$, where ${\mathbf{D}} = {\mathbf{P\Lambda }}^{ - 1} {\mathbf{P}}^{T}$. The control limit or threshold at the confidence level $(1 - \alpha )100\%$ is determined by the chi-squared distribution $\tau^{2} = \chi_{\alpha }^{2} \left( d \right)$, where latent dimension *d* is the degree of freedom.

#### Q index

The Q index monitors the residual subspace defined as $Q\left( {\mathbf{x}} \right) = {\mathbf{x}}^{T} {\tilde{\mathbf{C}}\mathbf{x}}$. The control limit is $\delta^{2} = \frac{{\theta_{2} }}{{\theta_{1} }}\chi_{\alpha }^{2} \left( {\frac{{\theta_{1}^{2} }}{{\theta_{2} }}} \right)$, where $\theta_{1} = \sum\nolimits_{i = d + 1}^{D} {\lambda_{i} }$, $\theta_{2} = \sum\nolimits_{i = d + 1}^{D} {\lambda_{i}^{2} }$ are computed with eigenvalues.

#### Combined index $\phi$

The combined index is defined as $\phi = {\mathbf{x}}^{T} \Phi {\mathbf{x}}$, where $\Phi = \frac{{{\tilde{\mathbf{C}}}}}{{\delta^{2} }} + \frac{{\mathbf{D}}}{{\tau^{2} }}$. The control limit is $\varsigma^{2} = g^{\varphi } \chi_{\alpha }^{2} \left( {h^{\varphi } } \right)$, where $g^{\varphi } = {{\left( {\frac{d}{{\tau^{4} }} + \frac{{\theta_{2}^{{}} }}{{\delta^{4} }}} \right)} \mathord{\left/ {\vphantom {{\left( {\frac{d}{{\tau^{4} }} + \frac{{\theta_{2}^{{}} }}{{\delta^{4} }}} \right)} {\left( {\frac{d}{{\tau^{2} }} + \frac{{\theta_{1}^{{}} }}{{\delta^{2} }}} \right)}}} \right. \kern-0pt} {\left( {\frac{d}{{\tau^{2} }} + \frac{{\theta_{1}^{{}} }}{{\delta^{2} }}} \right)}}$, and $h^{\varphi } = {{\left( {\frac{d}{{\tau^{2} }} + \frac{{\theta_{1}^{{}} }}{{\delta^{2} }}} \right)^{2} } \mathord{\left/ {\vphantom {{\left( {\frac{d}{{\tau^{2} }} + \frac{{\theta_{1}^{{}} }}{{\delta^{2} }}} \right)^{2} } {\left( {\frac{d}{{\tau^{4} }} + \frac{{\theta_{2}^{{}} }}{{\delta^{4} }}} \right)}}} \right. \kern-0pt} {\left( {\frac{d}{{\tau^{4} }} + \frac{{\theta_{2}^{{}} }}{{\delta^{4} }}} \right)}}$.

All indexes can be generalized as a quadratic form ${\text{Ind}}\left( {\mathbf{x}} \right) = {\mathbf{x}}^{T} {\mathbf{Mx}}$, and ${\mathbf{M}}$ is defined for each assigned index as per above. Please note that T^2^ and Q play asymmetric roles in health prediction, while the combined index merges both indexes into a single index. Theoretically, one can report an unhealthy situation given that any of the indexes exceeds the corresponding threshold.

### Bacteria-to-health-index contribution (BHC) inference

Once unhealthy conditions have been reported, health diagnosis aimed at identifying the responsible species that showed significant disease signals compared to the controlled healthy cohort. In accordance with the monitoring indexes, the BHC plots were induced for diagnosis. The diagnosis scheme was to reconstruct the normal status by adding a corrective term to the unhealthy composition. Assuming that species *i* has potential abnormal behaviors, the reconstructed composition unit can be expressed as:

5 $${\mathbf{z}}_{i} = {\mathbf{x}} - \xi_{i} f_{i}$$

where $\xi_{i}$ is the direction and $f_{i}$ is the magnitude. Then the objective can be formulated to optimize the health index as

6 $$\min Ind\left( {{\mathbf{z}}_{i} } \right) = \left( {{\mathbf{x}} - \xi_{i} f_{i} } \right)^{T} {\mathbf{M}}\left( {{\mathbf{x}} - \xi_{i} f_{i} } \right).$$

This can be done by taking the first derivative with respect to $f_{i}$ and then equaling it to zero, which finally yields

7 $$\phi_{i} = {\mathbf{x}^{T}{M}}\xi_{i} \left( {\xi_{i}^{T} {\mathbf{M}}\xi_{i} } \right)^{ - 1} \xi_{i}^{T} {\mathbf{Mx}}.$$

In real-world applications, the direction does not have to be a vector, as multiple species may be disordered in a specific disease. From this perspective, the BHC is preferred for health condition diagnosis.

### Contrastive PCA learning for health pattern discovery

To determine the underlying health patterns, we considered contrastive PCA learning on healthy and unhealthy populations reported by hiPCA. ${\mathbf{S}}_{h}$ and ${\mathbf{S}}_{uh}$ denote the covariance matrices of healthy and unhealthy cohorts, respectively, while contrastive PCA seeks to find the contrastive direction ${\mathbf{p}}^{ * }$ that can quantify the trade-off between having a high target healthy variance and low unhealthy variance by solving ${\mathbf{p}}^{ * } = \arg \max \left( {{\mathbf{S}}_{h} - \alpha {\mathbf{S}}_{uh} } \right)$. The contrast parameter $\alpha$ regulates the balance between healthy/unhealthy variances, which can be determined from a list of predefined sets [17]. Thus, healthy patterns can be highlighted through the elimination of unhealthy obfuscations. Once the contrastive direction was calculated and the latent projection completed, we used the Gaussian mixture model for unsupervised clustering, which can be determined by adjusting the number of components and tracking the Akaike information criterion (AIC) and Bayesian information criterion (BIC) for each fit.

### Metabolic subsystem analysis

In this study, we first mapped the species table to the genome-scale metabolic models (GSMM) [18] at the family rank or lower to at least one GSMM. The reactions were normalized to species abundance in the sample considered. We then performed a two-sample *t*-test for the reaction abundance from each pair of health patterns to identify reactions with significantly different abundance between each group. The metabolic subsystem of reactions was extracted from GSMM, and Fisher’s exact test was performed for the enrichment analysis of subsystems. Finally, the mean abundance differences in the subsystems were calculated in each health pattern pair for comparative analysis.

### Functional redundancy analysis

This study used the previous Genome Content Network (GCN) to calculate functional redundancy [19]. First, we searched all species in the species table in the Integrated Microbial Genome & Microbiome (IMG/M) database to construct a reference GCN. Here, we focused on the Human Microbiome Project (HMP). Representative strains of each species in the HMP project were randomly selected, if the species was not present in the HMP project, we randomly selected representative strains of each species. A reference GCN was constructed based on the retrieved representative strains, and the functional distance between two random species was calculated using the GCN. Finally, each sample’s taxonomic diversity, functional diversity, and functional redundancy were calculated by combining the functional distance and species table.

## Results and discussion

To evaluate the health prediction performance of hiPCA, we compared and validated large-scale metagenomic data. Metagenomic data were extracted from the GMHI study (referred to as the GMHI dataset), where 4347 preprocessed samples (2636 healthy and 1711 unhealthy individuals) were collected for model discovery and an additional 782 cohort (118 healthy and 664 unhealthy individuals) for validation purposes. In addition, a separate test cohort (consisting of 292 healthy and 313 unhealthy individuals) was included for further validation.

### hiPCA with health-scarce species can stratify healthy and unhealthy subgroups

**Fig. 2:**
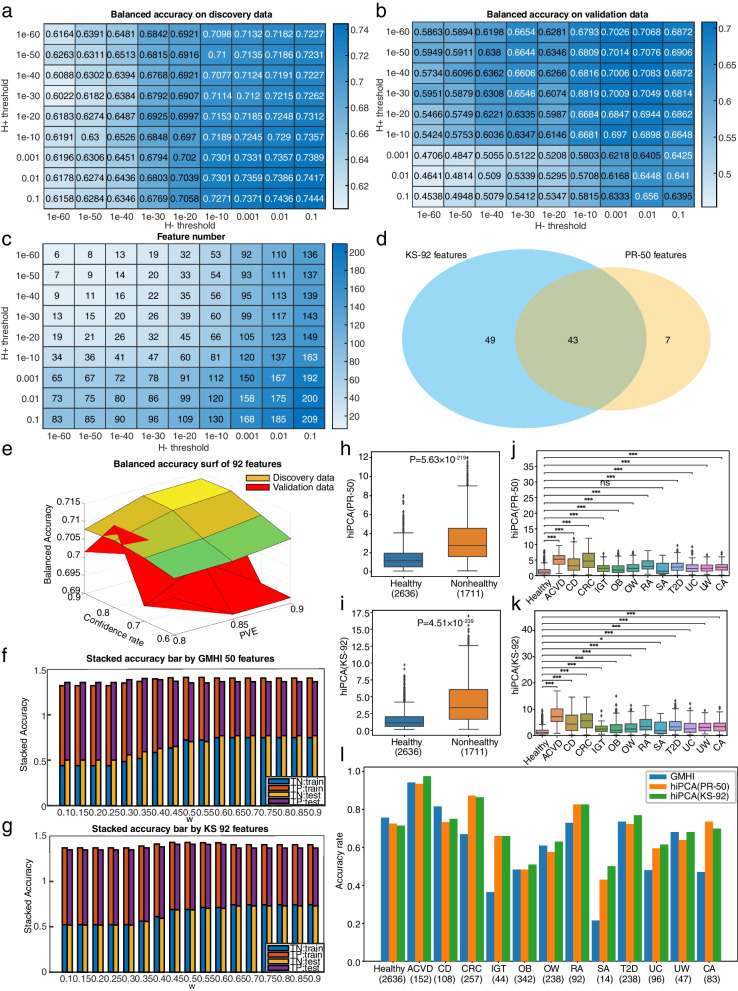
The hiPCA can stratify healthy and unhealthy groups. **a** The balanced accuracy of hiPCA under various H+/H− thresholds on discovery data (PVE 0.9 and confidence rate 0.9). **b** The balanced accuracy of hiPCA under various H+/H− thresholds on validation data (PVE 0.9 and confidence rate 0.9). **c** The respective features are selected under various H+/H− thresholds. **d** The intersection between GMHI 50 features and KS 92 features. **e** The surf plot of balanced accuracy by 92 features under different parameter configurations. The yellow zone represents the balanced accuracy results from the discovery set, while the red zone represents the balanced accuracy results from the validation set. **f** Stacked accuracy bar over different weighting factors from discovery data and test data with GMHI 50 features. **g** Stacked accuracy bar over different weighting factors from discovery data and test data with KS 92 features. TP and TN denote the true positive rate and true negative rate, respectively, and the balanced accuracy is the average between TN and TP in each single bar. **h**,**i** Box plot of hiPCA (PR-50 and KS-92) in healthy and nonhealthy groups. **j**,**k** Box plot of hiPCA (PR-50 and KS-92) in healthy and 12 nonhealthy phenotypes. **l** The hiPCA (PR-50 and KS-92) accuracy rates over different phenotypes. All* P*-values shown above the box plots were found using the two-sided Mann-Whitney *U* test: *, *P* ≤0.05; **, *P* ≤0.01; ***, *P* ≤0.001; ns, not significant. The sample size of each group is shown within parentheses

We first considered hunting the core species for health monitoring. The two-sample Kolmogorov-Smirnov test was performed on both the healthy and unhealthy groups of the discovery dataset. Then, abundance features were sorted according to the *P*-values in both groups. Health-prevalent features were defined by the rejection of the alternative hypothesis that the empirical cumulative density of healthy individuals was smaller than that of unhealthy individuals at the significance level of 0.1 and vice versa. After this step, 77 species were identified as health-prevalent (H+) species, and 136 species as health-scarce (H−) species. Based on the 209 species in total (four shared in both sets, as shown in Table S3), we were able to determine the ideal core set by evaluating the health prediction performance of our method. To investigate how health-prevalent and health-scarce taxonomic features may impact the monitoring performance, we made the sectional inspection at different significance levels ranging from 10^−60^ to 10^−1^, a smaller set point incurring a stricter selection standard. The balanced accuracy of hiPCA under various H+/H− thresholds on the discovery and validation datasets is shown in Fig. 2a–b. No H+ species were found below the threshold 10^−60^ (Fig. 2c). These results indicated that H− species were more appealing for health prediction, and hiPCA made desirable predictions at 92 H− species without H+ species. Interestingly, all 43 H− species engaged in GMHI were included in our subset (Fig. 2d). If we fixed the H− threshold at any set point, including more H+ species by adjusting the H+ threshold, we could only make a trivial contribution to the improvement in the discovery data, and in most cases, they were found to degrade the overall balanced prediction performance in the validation data. This result confirmed that the generalization of H+ features could become substandard in validation samples owing to the heterogeneity and unevenness in healthy populations.

Next, we discussed and investigated the trade-off between missing unhealthy alarms and false alarms. If the predictor reported that an unhealthy alarm is triggered when any of the indexes exceeded the threshold, there would be more false alarms. Conversely, if an unhealthy alarm is triggered only when all three indexes are triggered simultaneously, the alarm missing rate increased significantly. In practice, we found that either the majority rule or combined index alone can work well. For the former rule, hiPCA will report a disease alarm once any of the two indexes report a threshold violation. The latter rule only considers that the combined index as both *T*^2^ and Q have been combined into this index. Here, we used a combined index as the global health index. Therefore, one only needs to use the alarm missing and false alarm rates in the training set as indicators and then change the confidence and percentage of variance explained (PVE) until a satisfactory balance has been achieved (Fig. 2e). In fact, by doing so, our hiPCA can realize a customized health definition through the weighting factor scheme between true positive rate (TP, denoted by $\eta$) and false positive rate (FP, denoted by $\upsilon$). The global prediction performance index PI becomes ${\text{PI}} = w\upsilon + \left( {1 - w} \right)\eta$. We conducted a grid search investigation of the weighting factor, as shown in the stacking bar of Fig. 2f–g. We found that a desirable PI that considers the trade-off can be determined by setting $w$ in the range of [0.45,0.55]. It should be noted that the healthy can be flexibly user-defined by setting the weighting factor to a small value for those health critical conditions, whereas a balanced *w* can be configured for those normal situations. In either case, the balanced accuracy varied from 0.67 to 0.70 in both charts, which remained at a very stable high level for disease detection and health prediction.

We further evaluated the species-level health prediction performance among classifiers (see Table 1). The prefix is ​the feature selection method for all methods, and the suffix refers to the health prediction model. Using GMHI-hiPCA as an example, we used the prevalence-based strategy in GMHI for feature selection and hiPCA with the combined index for health monitoring. Generally, most classifiers achieved good results on the training data, but their generalization ability decreased dramatically on the validation dataset. In contrast, unsupervised hiPCA can achieve balanced results on both discovery and validation datasets. Interestingly, by transferring the microbial species features provided by GMHI, hiPCA can achieve overall comparable results to the original GMHI. However, by transferring the features from MRS, both GMHI and hiPCA deteriorate considerably, implying MRS features are largely methodology dependent.

**Table 1:** Health prediction results using different methods

Method	Feature number	Discovery dataset			Validation dataset		
		Healthy	Unhealthy	Average	Healthy	Unhealthy	Average
RF	313	1.0000	1.0000	1.0000	0.8220	0.5350	0.6780
XGB	313	1.0000	1.0000	1.0000	0.7120	0.5800	0.6460
GMHI	50	0.7560	0.6376	0.6970	0.7712	0.6220	0.6966
hiPCA	313	0.6988	0.7680	0.7334	0.5678	0.7259	0.6469
RF-hiPCA	50	0.7117	0.6645	0.6881	0.3305	0.7605	0.5455
XGB-hiPCA	50	0.7595	0.6078	0.6837	0.7119	0.5422	0.6270
GMHI-hiPCA	50	0.7242	0.6885	0.7063	0.7034	0.6461	0.6747
MIC-hiPCA	50	0.7303	0.6511	0.6907	0.3305	0.7244	0.5275
SPM-hiPCA	50	0.7470	0.6727	0.7098	0.6780	0.6235	0.6507
KS-hiPCA	50	0.7128	0.7031	0.7080	0.6864	0.6732	0.6798
KS-hiPCA	92	0.7140	0.7124	0.7132	0.7034	0.7018	0.7026
MRS-GMHI	6	0.6495	0.6172	0.6334	0.5508	0.6054	0.5781
MRS-hiPCA	6	0.8524	0.3641	0.6083	0.8475	0.3283	0.5879

Finally, we investigated the health prediction performance of hiPCA for 13 different phenotypes. As can be seen from Fig. 2h–k, the health index from PCA showed significant differences in the healthy group compared with that in the unhealthy groups. The overall balanced accuracy was similar to that of the GMHI (Table 1). However, considering the detection accuracy rates for each phenotype (Fig. 2l), we found that the detection rates in our hiPCA were 71.4% for healthy groups, whereas for unhealthy groups, the hiPCA vs. GMHI was 97.37% vs. 94.08% for arteriosclerotic cardiovascular disease (ACVD), 86.38% vs. 66.93% for colorectal cancer (CRC), 82.61% vs. 72.83% for rheumatoid arthritis (RA), 69.88% vs. 46.99% for colorectal adenoma (CA), 76.89% vs. 73.53% for type 2 diabetes (T2D), 68.09% vs. 68.09% for underweight (UW), 75% vs. 81.48% for Crohn’s disease (CD), 65.91% vs. 36.36% for impaired glucose tolerance (IGT), 63.03% vs. 60.92% for overweight (OW), 61.46% vs. 47.92% for ulcerative colitis (UC), 50.88% vs. 48.25% for obesity (OB), and 50% vs. 21.43% for symptomatic atherosclerosis (SA). One can see that our hiPCA outperformed GMHI in most unhealthy phenotypes. For hiPCA, the KS-92 feature set boosted the health detection rate but showed slightly lower disease alarming rates than PR-50 features in some unhealthy phenotypes. Nevertheless, the KS-92 panel achieved a better overall balanced accuracy in healthy and unhealthy populations. Our study revealed that (1) the proposed hiPCA performs outstandingly against existing methods with more stability under the core microbiome set by H− features; (2) our hiPCA can realize customized health standards for different wellness care levels and clinical conditions; and (3) the hiPCA can detect truly unhealthy groups much better than GMHI, which makes it more useful for health management in nonhealthy populations.

### Contrastive PCA learning discloses four healthy patterns

**Fig. 3:**
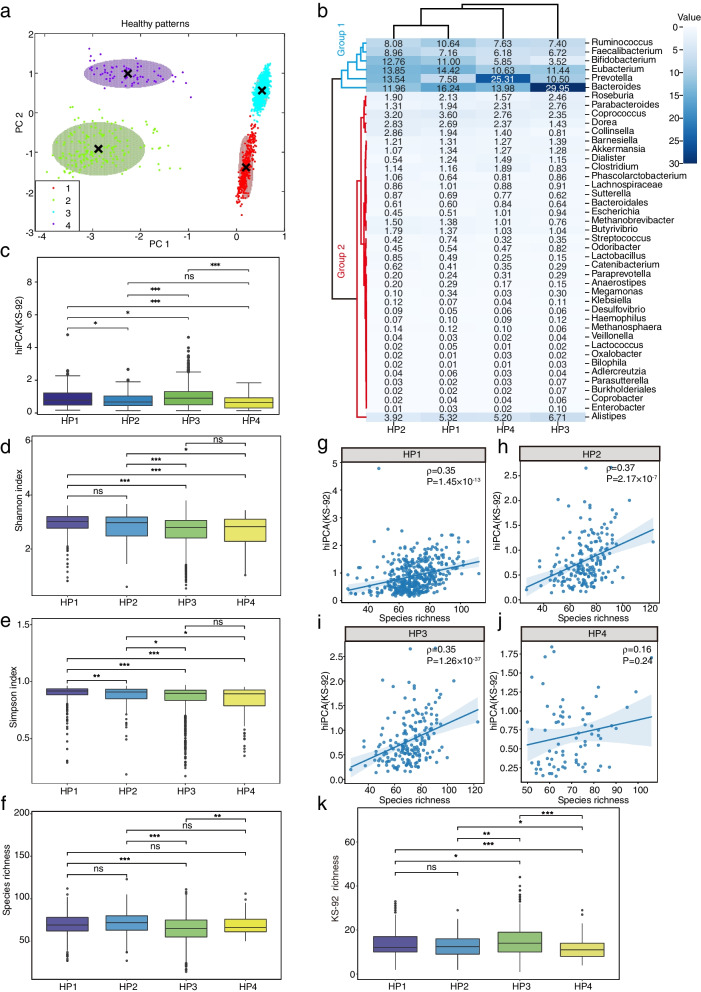
Comparative details among four healthy microbial composition patterns. **a** Clustering plot of healthy microbial composition patterns.** b** Thermal clustering plot of the population on averaged microbial compositions. *Bacteroides, Eubacterium, Faecalibacterium, Prevotella, Ruminococcus,* and *Bifidobacterium* genus for Group 1, others for Group 2. **c** Box plots for hiPCA(KS-92) distributions over four healthy patterns. **d–f** Alpha diversity of four healthy microbial composition patterns. **g–j** Correlation between hiPCA(KS-92) and species richness under four healthy patterns. **k** Richness of 92 H− species in four healthy patterns. All *P*-values shown above the box plots are found using the two-sided Mann-Whitney *U* test: *, *P* ≤0.05; **, *P* ≤0.01; ***, *P* ≤0.001; ns, not significant

The correlations between gut microbiota and health have been the subject of extensive discussions. However, evidence of a core taxa set that constitute a healthy gut is still lacking. To explore healthy patterns, we made the basic assumption known as the “Anna Karenina principle”: unhealthy ways vary more than healthy ways [20]. On this basis, and to exhibit the most differences across healthy and unhealthy populations, we performed contrastive PCA learning on the species level of all hiPCA reported healthy populations, whereas the unhealthy samples were used as the background dataset and healthy samples as the target foreground. The objective of contrastive PCA learning is to obtain low-dimensional projections with high target variance and low background variance. Once such principal components have been obtained, the Gaussian mixture model can be used to perform unsupervised clustering under the AIC fit criteria. After the contrastive learning, we identified four health patterns (Fig. 3a).

**Fig. 4:**
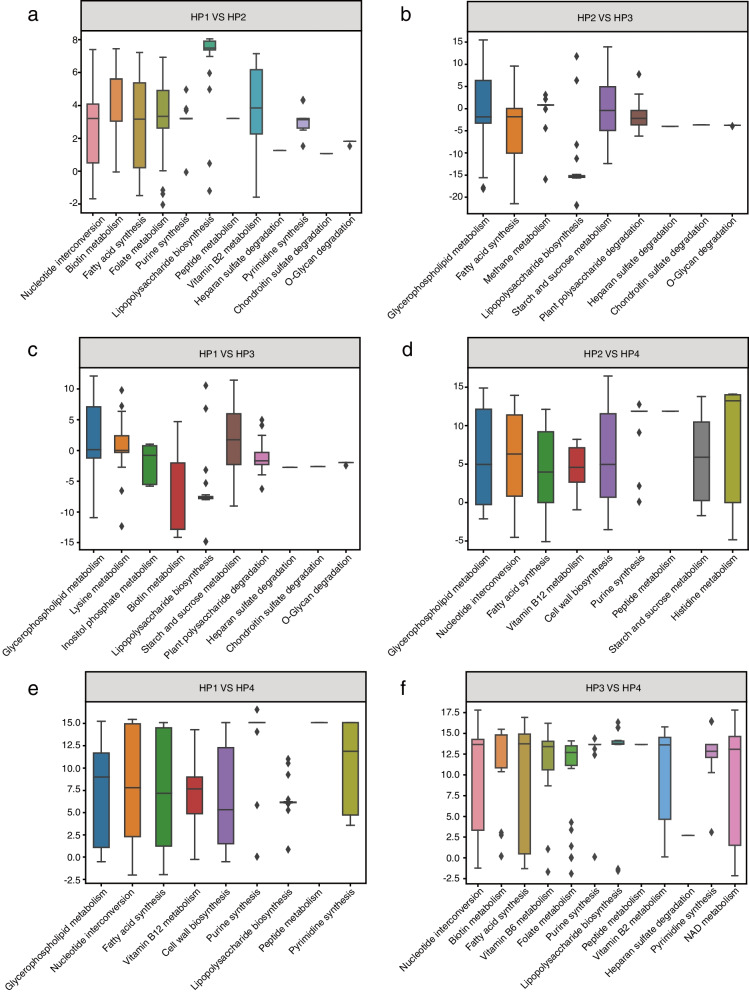
Metabolic subsystems in different healthy compositional patterns. **a–f** Comparison of metabolic subsystems with different healthy composition patterns. Only subsystems with significant differences (*P *<0.01) would be considered enriched

Following healthy pattern identification, we could examine each pattern’s microbial composition. By accumulating the species abundance into genus level and excluding low-abundance genera, the comparative details among four healthy patterns were presented. As shown in Fig. 3b, there were distinctions and connections. A cursory glance indicated that all patterns shared those most dominant genera, including *Bacteroides*, *Bifidobacterium*, *Eubacterium*, *Faecalibacterium*, *Prevotella*, and *Ruminococcus*. Interestingly, the *Bifidobacterium* genus showed an increased prevalence in both HP1 and HP2, which might be connected with milk-associated diets [21]. In addition, there were also various discrepancies among detailed compositions. Particularly, both *Bifidobacterium and Faecalibacterium* were elevated in HP1/2 compared with HP3/4. The HP3 cluster was mainly characterized by *Bacteroides*, whereas the HP4 cluster was driven by *Prevotella*. Interestingly, the abundance of the *Prevotella* genus showed varying ratios compared to *Bacteroides* in all four baseline patterns, the *Prevotella*-to-*Bacteroides* (P/B) ratio was roughly estimated as 1:2 in HP1 baseline, 1:1 in HP2 baseline, 1:3 in HP3 baseline, and 2:1 in HP4 baseline. Apart from such discrepancies, the two-sided Mann-Whitney *U* test showed significant differences in hiPCA levels among different healthy patterns (Fig. 3c), indicating that HP2 could be superior to HP1 and HP3 when contributing to host health. Moreover, the health index and diversity plots in Fig. 3d–f indicated that HP3 had a lower level of richness and a higher hiPCA level, which implied a substandard health status. Interestingly, by considering the hiPCA and species richness correlations among four health patterns, we found significant positive correlations for all patterns except HP4 (Fig. 3g–j). A stronger correlation indicated that (1) a slight increase in species richness could result in high unhealthy risks due to greater engagement of H− species and (2) more modulation efforts could be required for H− species depletion to shift the microbial composition toward a healthier pattern. To determine this, we computed richness for all health patterns using 92 H− species, and HP3 was significantly higher in H− richness than the other patterns (Fig. 3k). Lastly, we investigated the functional context of the microbial composition in each health pattern using metabolic reaction set analysis. As shown in Fig. 4, the different healthy compositional patterns were consistent with the differences in metabolic functions. For HP3, there was significant enrichment in bacterial biotin metabolism, which is a vital component in host physiological activities, such as carbohydrate and lipid metabolism [22–24]. In addition, HP3 exhibited a higher level of lipopolysaccharides (LPS), which acted as the prototypical endotoxin and was associated with health effects, such as obesity [25], diabetes [26], cardiovascular diseases [27], and insulin sensitivity [28]. From this perspective, we speculated that HP3 could be regarded as a substandard health style.

### hiPCA has reasonable correlations with species diversity, functional redundancy, and host physiological measure

**Fig. 5:**
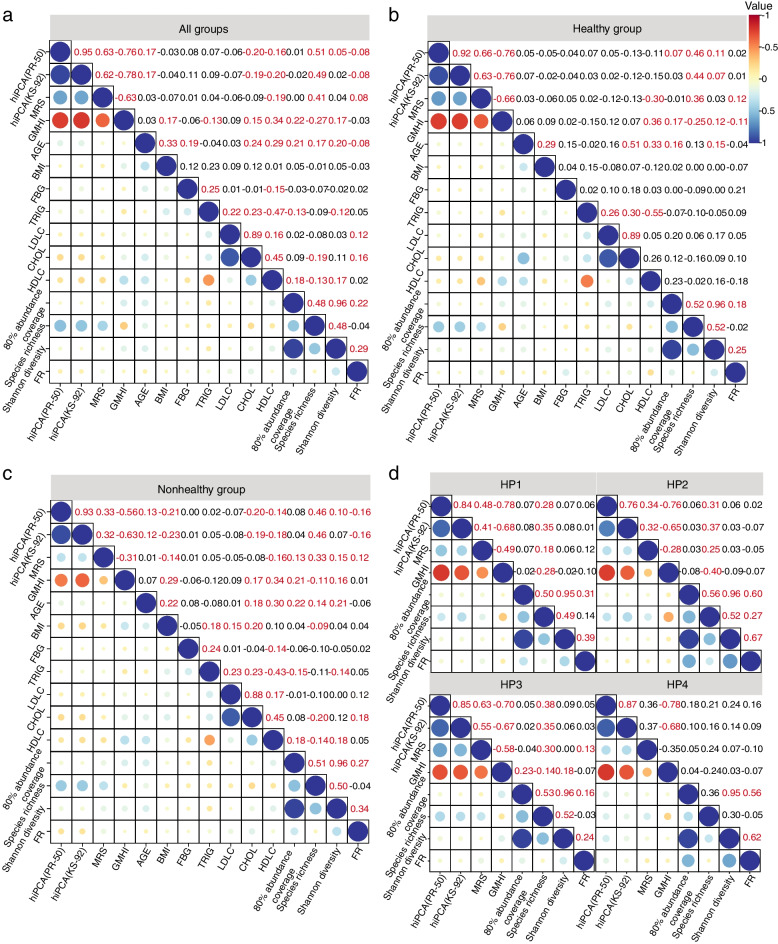
Correlation analysis for the health index and other indexes. The heat map of the intra-group correlation analysis of variable pairs is displayed along the lower left corner of the matrix. The correlation of variable pairs appears in the upper right corner of the matrix. The correlation coefficients highlighted in red indicate that the pairs of variables have significant correlations (*P *<0.001).** a** Heat map of correlation analysis in all groups.** b** Heat map of correlation analysis in the healthy group. **c** Heat map of correlation analysis in the nonhealthy group. **d** Heat map of correlation analysis in four health patterns

First, we considered its association with community diversity. Alpha diversity is a popular metric used for ecological community analysis because of its correlations with productivity, functionality, and stability [29]. It is interesting to find that the correlation conclusions differ across the whole population, healthy/nonhealthy divided subgroups, and health pattern divided subgroups. By analyzing the inter-relationships shown in Fig. 5a–d, all health predictors consistently correlated with species richness in most cases. This implied that species richness could be strongly linked to health status [30], which also provided evidence of unhealthy risks in healthy people, as all health predictors showed that an increased richness could be associated with unhealthy gut microbiota. Consequently, we inferred that richness is a biased indicator of healthy microbiota. As found in previous research, richness primarily reflects the ecosystem development stage in the gut or is an indicator of gut ecosystem age [31]. For Shannon diversity, which presented community heterogeneity, only weak correlations appeared with hiPCA(KS-92) in the healthy subgroup, but no significant correlations were reported with health predictors in the combined groups or the nonhealthy subgroup. Moreover, both GMHI and hiPCA revealed no significant correlations in most subgroups assigned to health patterns; GMHI only showed a positive association with HP3. Interestingly, our hiPCA results indicated that healthy people were inclined to present some orderly ecological community organizations but retained the margin of variation, contrary to the nonhealthy subgroup. Accordingly, we verified the previous assumption that a higher Shannon diversity may not always imply better health [32], as the enrichment of pathogenic bacteria may also lead to increased Shannon diversity.

Despite species compositional diversity, metabolic function is usually regarded as much less diverse and highly conserved in healthy populations. However, it remains an open challenge to reasonably describe the correlation between functional redundancy (FR) and health status. To this end, we first performed the FR analysis with different health indexes for (1) all samples, (2) health samples only, and (3) unhealthy samples only. Analyzing the results from Fig. 5a–c, we found that FR showed significantly adverse correlations with hiPCA on the entire meta-data, especially in the unhealthy cohort, implying potential functional redundancy loss in unhealthy populations. However, functional redundancy was not significantly related to the health index among healthy individuals. Our findings strengthen the evidence regarding the conversed properties of micro-ecological functions in healthy populations. In contrast, the GMHI reported insignificant correlations with FR in unhealthy people and negative correlations in healthy people. In other words, GMHI preferred a lower FR in the healthy cohort. This could contradict the mainstream perspective that an increased level of FR generally plays a role in stabilizing microbiota functions during perturbations, which is a positive signal for health promotion. Likewise, MRS leveraged positive correlations with FR in both healthy and unhealthy cohorts, which may also become confusing for health representation. We found no significant correlations between FR and health index by further inspecting the function-hiPCA relations in the healthy subgroup divided into health patterns (Fig. 5d). Our results revealed that the health patterns were quite different but also had certain common functions that contribute to host health.

Finally, physiological measures are routinely adopted as health indicators, and it is important to connect PCA-based health index with physiological measures. In this study, all subjects’ phenotypes, such as age, body mass index (BMI), cholesterol (CHOL), fasting blood glucose (FBG), triglycerides (TRIG), high-density lipoprotein cholesterol (HDLC), and low-density lipoprotein cholesterol (LDLC), were considered for health index association analysis. To conduct a rational analysis, we first used the filtering strategy on each physiological index to eliminate outlying records, and then Spearman’s correlation coefficients were calculated between all pairs of index variables. As shown in Fig. 5a–c, in healthy controls, both GMHI and MRS showed meaningful correlations with HDLC, implying that a higher level of HDLC was better, even in healthy populations. However, our hiPCA reported that none of the physiological measures significantly correlated with the health index among healthy populations, including HDLC. Just as the classic HDL hypothesis, “*intervention to raise HDLC concentrations will reduce cardiovascular risk*” is questionable as raising HDLC levels may have no effect on reducing cardiovascular risk [33]; therefore, we still could not come to the sound conclusion that a higher level of HDLC was conclusively associated with the better health status in healthy populations. We observed that BMI and HDLC were associated with all health indexes for the case subgroup, implying potential abnormal weight loss and HDLC loss in diseased populations. Interestingly, although used with different species, hiPCA using PR-50 and KS-92 feature sets showed consistent associations. More importantly, hiPCA presented meaningful correlations with chronological age in the entire population. The human microbiota is significantly associated with the aging process and is usually considered as an important healthy aging modulator [34]. The microbiota alterations during aging may imply accelerated age-related health deterioration in some subjects [35]. A recent study reported that age-related physiological changes in older adults, rather than those in diet and lifestyle, could have profound effects on the human gut microbiota [36]. However, this does not mean healthy seniors always had a bad health index in our hiPCA. In fact, we took a further step by abandoning unhealthy samples and recomputing the correlations with only healthy populations, and the results indicated that the hiPCA had no significant correlations with age in healthy populations, and significant correlations were only reported for nonhealthy populations. From this perspective, our health index was reasonably associated with physiological health status and had good application prospects in a wide range of populations.

### Personalized inference reveals disease-specific microbial responses

**Fig. 6:**
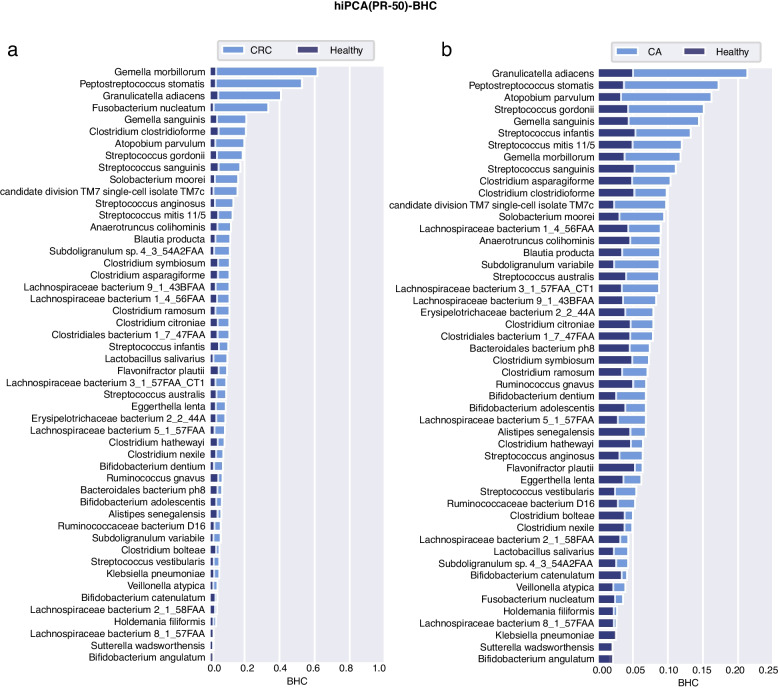
BHC bar plot for the healthy and two disease subgroups under PR-50 features. The background dark blue bar denotes the population-averaged BHC in the healthy subgroup, and the light blue bar represents the population-averaged BHC in the disease subgroup. **a** CRC, **b** CA

**Fig. 7:**
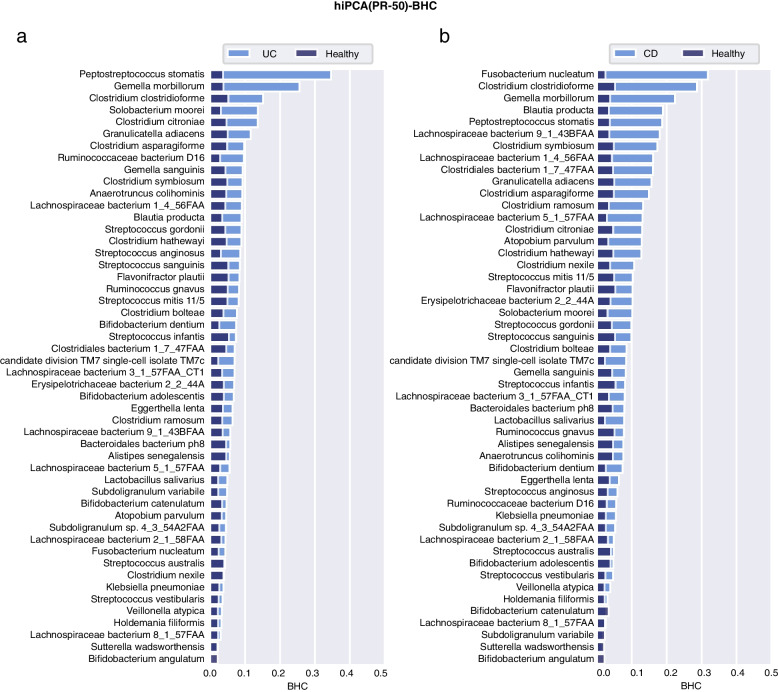
BHC bar plot for the healthy and two disease subgroups under PR-50 features. **a** UC, **b** CD

**Fig. 8:**
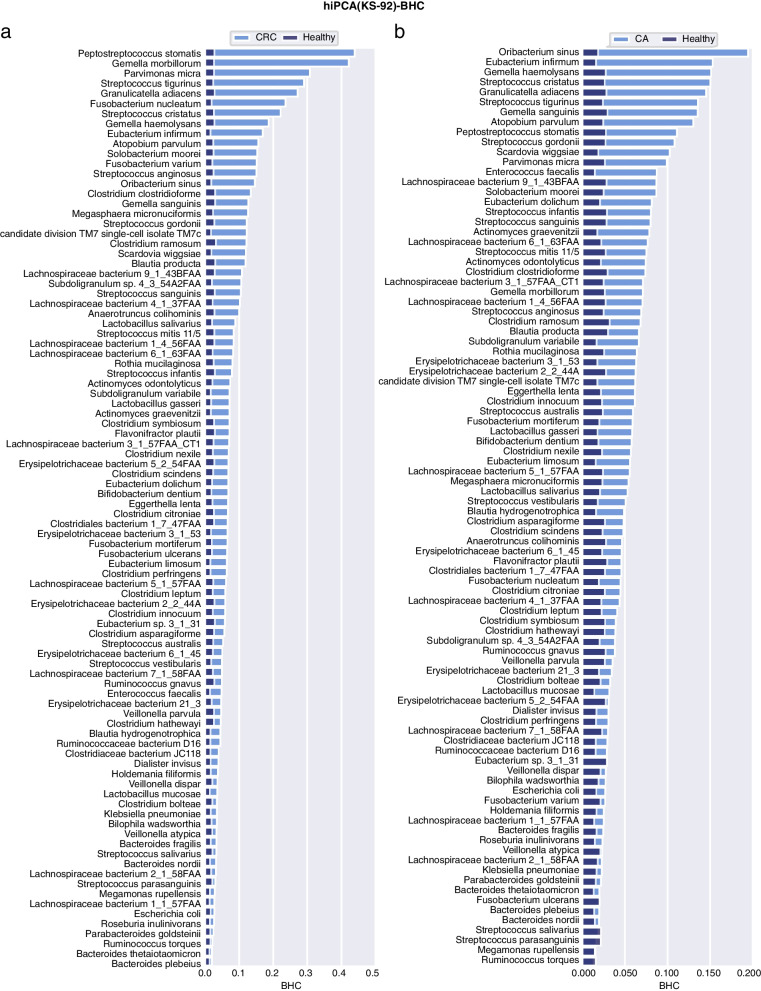
BHC bar plot for the healthy and two disease groups under KS-92 features. **a** CRC, **b** CA

**Fig. 9:**
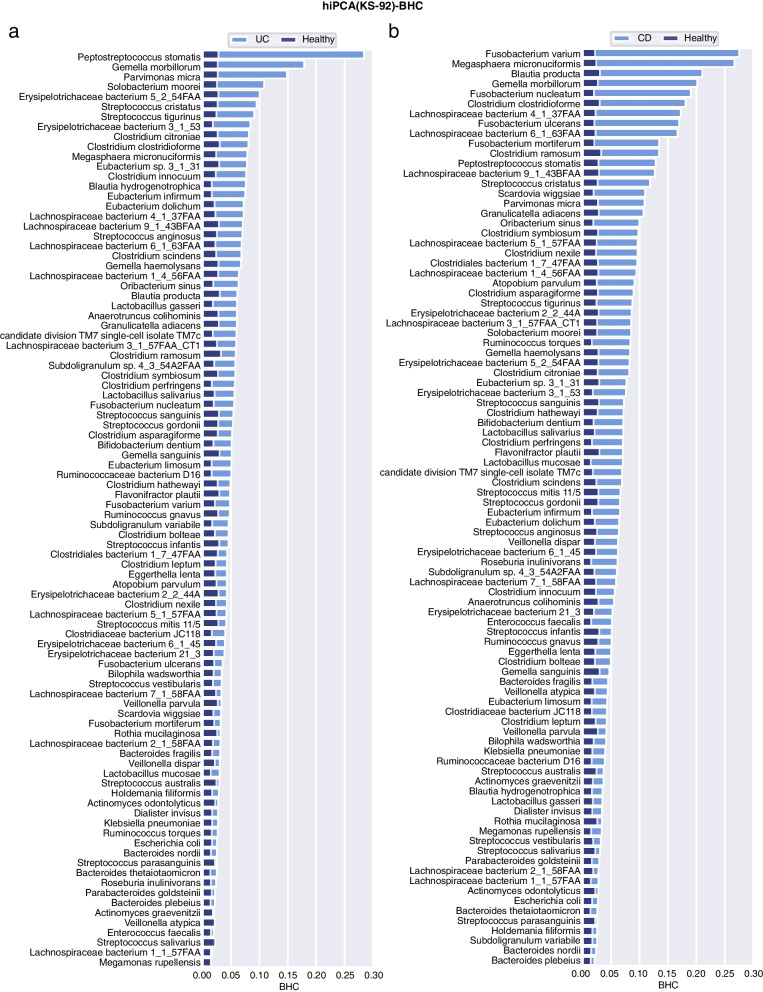
BHC bar plot for the healthy and two disease groups under KS-92 features. **a** UC, **b** CD

To thoroughly examine the diagnostic capability of hiPCA, we investigated the contribution of each biomarker to each person’s health index. However, to comprehensively evaluate populations and find meaningful biomarkers, analyses were performed on the population-averaged BHC with both PR-50 and KS-92 features. Through BHC inference with hiPCA, we derived the contribution panel of all species to the health index. To observe this, the overlay bar graph over microbial features regarding health and four diseases (CRC, CA, UC, and CD) are shown in Figs. 6, 7, 8, and 9, and additional diseases are analyzed in the Additional file 4: Supplementary note 5. One can judge that most of the high-contribution H− species in PR-50 were also shared in the KS-92 scenario. As the KS-92 contribution plot can be viewed as a broad panel for contribution analysis, the following discussion will be based on the integrated analysis under both PR-50 and KS-92 contribution plots.

#### CRC and CA

CRC begins as a benign adenomatous polyp and then progresses into a CA, which winds up as an invasive cancer [37]. CRC development was previously considered to correlate strongly with intestinal microbiota [38]. By exploring the hiPCA performance for CRC diagnosis, these marker species contributed the most to the health index in PR-50, which could be listed as *Peptostreptococcus stomatis*, *Gemella morbillorum*, *Fusobacterium nucleatum*, *Granulicatella adiacens*, *Solobacterium moorei*, *Atopobium parvulum*, and *Streptococcus* spp. KS-92 shared the most contributors but was supplemented with *Parvimonas micra*, *Streptococcus tigurinus*, *Streptococcus cristatus*, and *Eubacterium infirmum*. Among them, the oral pathogens *Peptostreptococcus stomatis* and *Fusobacterium nucleatum* were among the most discriminative enriched species and were the most reported, suggesting an oral-gut translocation route [39–44]. *F. nucleatum* has been reported to promote oncogenic effects through the E-cadherin/$\beta$-catenin signaling pathway, which activates downstream pro-inflammatory responses [45]. In addition, other species, such as *Streptococcus* and *Lachnospiraceae*, were found to be significantly different in patients with CRC than that in healthy populations [46–48]. For instance, *S. anginosus* was shown to have a high discriminatory capacity in a biomarker panel for CRC diagnostic classification [49]. Recently, *P. micra* was reported as a putative non-invasive fecal biomarker for CRC [50]. Several bacterial species, including *Peptostreptococcus stomatis*, *Fusobacterium nucleatum*, and *Parvimonas micra*, were enriched in CRC, which were highly similar to our results [51]. Advanced adenomas are regarded as clinical precursors of CRC. According to the results, high contributors for CA shared the most part as CRC, including *Atopobium parvulum*, *Granulicatella adiacens*, *Oribacterium sinus, Peptostreptococcus stomatis*, *Gemella sanguinism*, *Subdoligranulum variabile*, *Solobacterium moorei,* and *Gemella morbillorum*. In addition, several *Lachnospiraceae* species, along with *Streptococcus* species showed statistical discrepancies from healthy controls. In a recent study, *Atopobium parvulum* was defined as a “progressive microbiota biomarker” from the control to advanced adenoma and then to the CRC group [52]. However, *F. nucleatum* did not seem to be an intensively significant identifier, as was found in the CRC population. This was in line with a previous study [53], where they found that the *F. nucleatum* in CA cases was 3.8-fold higher than that in the controls (*P* = 0.022); however, for CRC, it became 132-fold higher (*P* < 0.001). Nevertheless, further efforts are required to answer mechanistic questions regarding the role of these bacteria in tumor initiation and progression [42].

#### UC and CD

UC and CD are the two major subtypes of inflammatory bowel disease (IBD). They share some clinical and pathological features, and variability in disease distributions [54]. The detailed discussion of the mechanism of such variability is beyond our scope, but here we are interested in comparing the averaged population-level contributions. In general, UC and CD share several contributors, although their variations in UC were not as significant as those in CD. For UC, high-contribution candidates include *Peptostreptococcus stomatis*, *Gemella morbillorum*, *Parvimonas micra*, *Solobacterium moorei*, *Ruminococcaceae bacterium, Streptococcus cristatus, Streptococcus tigurinus*, and *Erysipelotrichaceae bacterium*. Interestingly, KS-92 indicated that *P. stomatis*, *G. morbillorum*, and *P. micra* were the top three contributing species to UC, which were exactly the same top three H− species as in CRC, indicating that patients with UC may have high risk associations with CRC. In a multidisease study, *G. morbillorum* and *P. stomatis* showed strong coaggregation in UC regardless of the data sources [55]. Moreover, commensal *Peptostreptococcus* species have been reported to produce indoleacrylic acid and suppress inflammation [56]. For CD, these candidates became more evident, including *Fusobacterium nucleatum*, *Fusobacterium varium*, *Clostridium clostridioforme*, *Blautia producta*, *Gemella morbillorum*, *Lachnospiraceae bacterium 4_1_37FAA*, and *Megasphaera micronuciformis*. Among them, *F. nucleatum* has long been associated with the etiology of IBD, particularly CD [57]; *C. clostridioforme* was previously observed to be enriched in patients with CD [58]. *B. producta* is a butyrate-producing bacterium involved in the design of bacterial consortia for treating patients with IBD [59]. *F. varium* has been previously studied as an infectious bacterium that can cause IBD [60, 61]. Here, we observed that it was a high contributor species in both CD and UC and was even higher in CD than that in UC. Despite the great variability between UC and CD, we could observe that both UC and CD should be linked with oral lesions. The role of the oral microbiome in pathogenesis can be partly attributed to ectopic colonization [62]. Specifically, we also observed that *K. pneumoniae* and *A. parvulum* show more variations in CD. A recent study revealed the ectopic colonization mechanism of oral *Klebsiella* strains when the intestinal microbiota is dysbiotic [63]. In contrast, *A. parvulum* was found to induce pancolitis in colitis-susceptible interleukin-10-deficient mice, which provided novel mechanistic insights into CD pathogenesis [64].

### Contribution spectrum analysis identifies broad-spectrum disease-related species

**Fig. 10:**
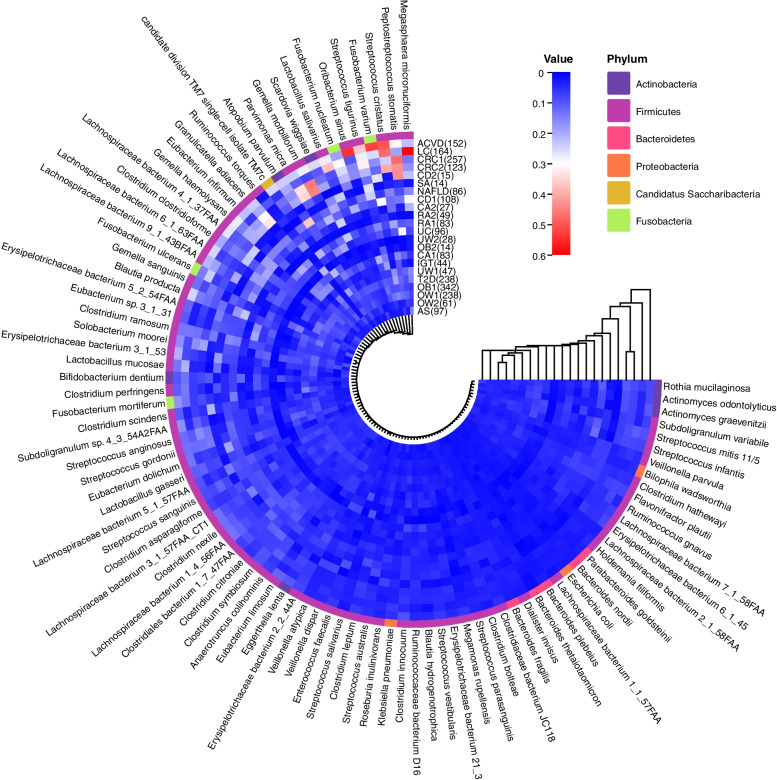
BHC spectrum clustering heat map for 15 disease subgroups under KS-92 features. Here, CRC1 represents CRC in the discovery set, CRC2 represents CRC in the validation set, and the same rule also applies to other diseases

**Fig. 11:**
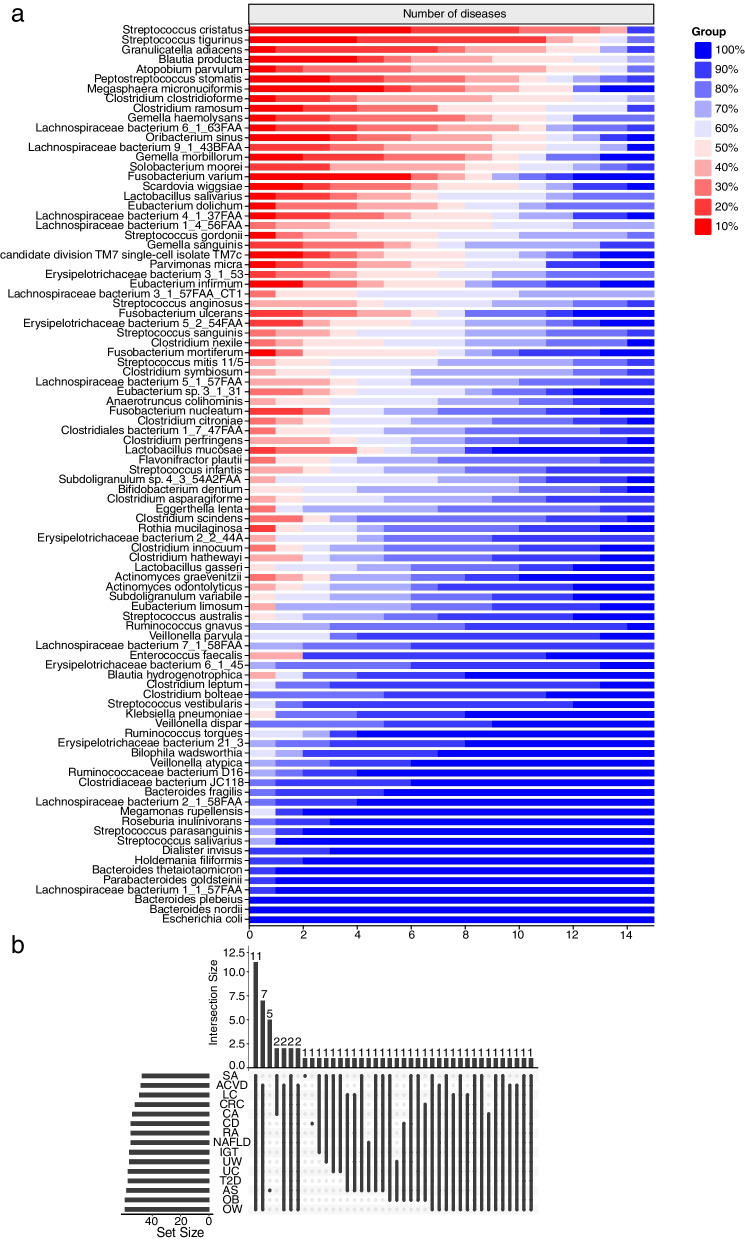
Intersection of broad-spectrum species with high contribution among 15 diseases in the discovery and validation sets.** a** Distribution of each species in diseases under different ratios of accumulated contribution over total contribution. **b** Intersection of high-contribution species among multiple diseases under the ratios of accumulated contribution over total contribution reached 80%

We found several interesting associations among different diseases through clustering analysis of the health contribution spectrum of species across all diseases in both the discovery and validation cohorts. As shown in Fig. 10, most of the diseases existing in both cohorts will be clustered together by contribution spectra, including CRC, RA, and OW. For CD, CA, UW, and OB, the contribution spectra in the validation set were not neighbors with the discovery counterpart, which we speculate should be due to the insufficient samples in the validation set. Nevertheless, each pair from the same disease was still assigned to adjacent positions. Metabolic disorder-related diseases, such as OB, T2D, and IGT, were closely clustered, which directly implied a common pathogenesis in related diseases. UC had close clustering with RA, and many studies revealed that a large number of patients with UC developed RA within a few years. In addition, UC also had closed clustering with CA, which confirmed previous findings that the prevalence of CA among patients with UC is high [65, 66]. Furthermore, we found that SA and RA were closely related to UC and CD. UC, CD, RA, and SA are immune-mediated inflammatory diseases (IMIDs), which may share some underlying pathogenic features [67]. Of note, the microbiome health contribution spectrum of non-alcoholic fatty liver disease (NAFLD) remained close to that of IMIDs, which was also reasonable as the main molecular and immunological mechanisms in NAFLD were regarded to be shared with IMIDs. The bacterial contributions of 12 diseases in the discovery cohort and three diseases in the validation cohort were further analyzed. As shown in Fig. 11a, the red bands indicated that bacteria made an important contribution to the health index among multiple diseases. Subsequently, we further made the classifier prediction performance under different ratios of accumulated contribution over total contribution and found that the prediction performance was best with the ratio 0.8 (Table 2). We chose the species for which the ratio of accumulated contribution to total contribution reached 80% as the high-contribution species. Considering all 12 diseases in the discovery cohort, we found 12 shared high contributors including *Streptococcus tigurinus*, *Gemella haemolysans*, *Lactobacillus salivarius*, *Blautia producta*, *Atopobium parvulum*, *Erysipelotrichaceae bacterium 3_1_53*, *Clostridium clostridioforme*, *Solobacterium moorei*, *Eubacterium dolichum*, *Lachnospiraceae bacterium 1_4_56FAA*, *Streptococcus gordonii*, and *Lachnospiraceae bacterium_3_1_57FAA_CT1* (Table 3). A recent gene-level analysis study reported *G. haemolysans*, *S. moorei*, *Erysipelotrichaceae*, and *Streptococcus* as potentially broad-spectrum multidisease-associated species [68]. Adding three new diseases (Ankylosing spondylitis [AS], Liver cirrhosis [LC], and NAFLD) in the validation set resulted in a reduced broad spectrum covering 11 high contributors (Fig. 11b), and only *S. moorei* was not included due to the absence of AS, which further demonstrated the powerful diagnostic ability of our hiPCA framework and offered new insights into potential microbiome targeted therapy [67].

**Table 2:** Classification performance using RF under different levels of features covering total contributions

Diseases	Accumulated contribution percentage for feature selection							KS-92 features	All 313 features
	0.3	0.4	0.5	0.6	0.7	0.8	0.9		
ACVD	0.731	0.738	0.747	0.762	0.752	0.791	0.788	0.770	0.784
SA	0.617	0.708	0.592	0.492	0.633	0.600	0.683	0.600	0.883
CRC	0.652	0.765	0.746	0.762	0.815	0.794	0.789	0.827	0.828
CA	0.539	0.463	0.550	0.529	0.554	0.632	0.621	0.523	0.554
UC	0.706	0.809	0.807	0.797	0.773	0.742	0.696	0.668	0.725
CD	0.862	0.868	0.878	0.934	0.934	0.959	0.950	0.953	0.986
T2D	0.575	0.665	0.623	0.693	0.712	0.696	0.694	0.618	0.591
IGT	0.674	0.487	0.530	0.560	0.622	0.562	0.462	0.540	0.592
RA	0.525	0.579	0.613	0.616	0.576	0.629	0.646	0.631	0.571
OB	0.723	0.706	0.756	0.786	0.773	0.777	0.800	0.800	0.883
OW	0.467	0.511	0.571	0.551	0.539	0.621	0.509	0.454	0.525
UW	0.497	0.683	0.733	0.811	0.751	0.805	0.737	0.638	0.516
Averaged	0.602	0.658	0.683	0.701	0.694	0.721	0.698	0.659	0.664

^a^The BHC of each species among diseases in the discovery cohort is ranked in descending order, and the total contribution is $\sum\nolimits_{i=1}^D{\mathrm{BHC}}_{x_i}$. Features $x_{1} ,x_{2} ,...,x_{p}$ are selected until the ratio of accumulated contribution $\sum\nolimits_{i = 1}^{p} {{\text{BHC}}_{{x_{i} }} }$ to total contribution exceeds the given percentage $\eta$, that is ${{\sum\nolimits_{i = 1}^{p} {{\text{BHC}}_{{x_{i} }} } } \mathord{\left/ {\vphantom {{\sum\nolimits_{i = 1}^{p} {{\text{BHC}}_{{x_{i} }} } } {\sum\nolimits_{i = 1}^{D} {{\text{BHC}}_{{x_{i} }} } }}} \right. \kern-0pt} {\sum\nolimits_{i = 1}^{D} {{\text{BHC}}_{{x_{i} }} } }} > \eta$. Once the features have been selected, the RF is used for classification evaluation.

^b^Excluding the SA with the least sample size, the CD with the best AUC result, and the IGT with the worst AUC result, the remaining AUC results were averaged, and when the ratio exceeded 0.8, the average AUC reached the best

**Table 3:** The shared high contributors among diseases

12 shared high contributors among 12 diseases in the discovery set	11 shared high contributors among 15 diseases in the discovery and validation set
*Erysipelotrichaceae bacterium 3_1_53*	*Clostridium clostridioforme*
*Clostridium clostridioforme*	*Streptococcus gordonii*
*Streptococcus gordonii*	*Streptococcus tigurinus*
*Streptococcus tigurinus*	*Blautia producta*
*Blautia producta*	*Lachnospiraceae bacterium 3_1_57FAA_CT1*
*Lachnospiraceae bacterium 3_1_57FAA_CT1*	*Gemella haemolysans*
*Gemella haemolysans*	*Lachnospiraceae bacterium 1_4_56FAA*
*Lachnospiraceae bacterium 1_4_56FAA*	*Atopobium parvulum*
*Atopobium parvulum*	*Lactobacillus salivarius*
*Lactobacillus salivarius*	*Eubacterium dolichum*
*Eubacterium dolichum*	*Erysipelotrichaceae bacterium 3_1_53*
*Solobacterium moorei*	

## Conclusion

This study presented an effective and interpretable gut microbiome health monitoring diagram to quantify and diagnose personal health status. Our monitoring framework was constructed with a healthy population understanding based on statistical inference theory to infer any deviation from the nominal health level with the universal boundary. Under this framework, we found that the microbiome can reflect healthy status or potential unhealthy risks by employing only H− species. Four health patterns can be determined in healthy population analysis after contrastive learning. The average health patterns with various P/B ratios and different levels of health superiority were discussed among health patterns. Our health index was reasonably associated with the diversity index, physiological measures, and functional redundancy. More importantly, the BHC spectrum can leverage personalized health diagnosis, which further discloses those diseases shared and specific diagnostic indicators by aggregating the population samples for potential clinical investigation and modulation analysis.

Despite the strong reproducibility and interpretability of the hiPCA, some limitations were noted. For instance, hiPCA is constructed with a linear embedding framework, which cannot unveil nonlinear bacterial interaction patterns. From the information integrity, further information such as the contextualization of the microbiota community to metatranscriptomics and metabolism may allow high data quality and accurate predictions. Nevertheless, as a general framework, we believe that hiPCA greatly facilitates the individualized assessment of health status and identification of potential biomarkers, contributing to a comprehensive understanding of the roles of gut microbiota in personalized human health.

### Supplementary Information

**Additionalfile 1.** 

**Additionalfile 2.** 

**Additionalfile 3.** 

**Additionalfile 4.**

## Data Availability

For the discovery cohort and validation cohort, we used GMHI data. For the test cohort, all sequencing data for this analysis can be obtained from the European Nucleotide Archive (ENA) databases, and the project numbers are PRJEB27005, PRJEB29127, PRJNA449784, PRJNA504891, PRJNA529124, PRJNA529400, and PRJNA531203. The datasets supporting the conclusions of this article are included within the article (Additional file 1). The gut microbiome analysis codes generated in this study are available at this link: https://github.com/XieHeq/hiPCA.

## Abbreviations

GMHI: Gut microbiome health index
PCA: Principal component analysis
MRS: Microbiome risk score
RF: Random forest
XGB: eXtreme Gradient Boosting
SPC: Spearman’s correlation
MIC: Maximum information coefficient
hiPCA: Health index with PCA
SVD: Singular value decomposition
PCs: Principal components
PCS: Principal component subspace
RS: Residual subspace
PEVs: Percentage of explained variances
BHC: Bacteria-to-health-index contribution
AIC: Akaike information criterion
BIC: Bayesian information criterion
GSMM: Genome-scale metabolic models
GCN: Genome content network
IMG/M: Integrated microbial genome & microbiome
HMP: Human microbiome project
FR: Functional redundancy
IBD: Inflammatory bowel disease
ACVD: Arteriosclerotic cardiovascular disease
CRC: Colorectal cancer
RA: Rheumatoid arthritis
CA: Colorectal adenoma
T2D: Type 2 diabetes
UW: Underweight
CD: Crohn’s disease
IGT: Impaired glucose tolerance
OW: Overweight
UC: Ulcerative colitis
OB: Obesity
SA: Symptomatic atherosclerosis
AS: Ankylosing spondylitis
LC: Liver cirrhosis
NAFLD: Non-alcoholic fatty liver disease
