# Supplementary material for: Statistical modeling of gut microbiota for personalized health status monitoring
Source: Microbiome. 2023 Aug 18;11:184. doi: 10.1186/s40168-023-01614-x (PMC10436630; doi:10.1186/s40168-023-01614-x)
Supplement: Supplementary file 3 — Additionalfile 2. [file 40168_2023_1614_MOESM2_ESM.pdf]

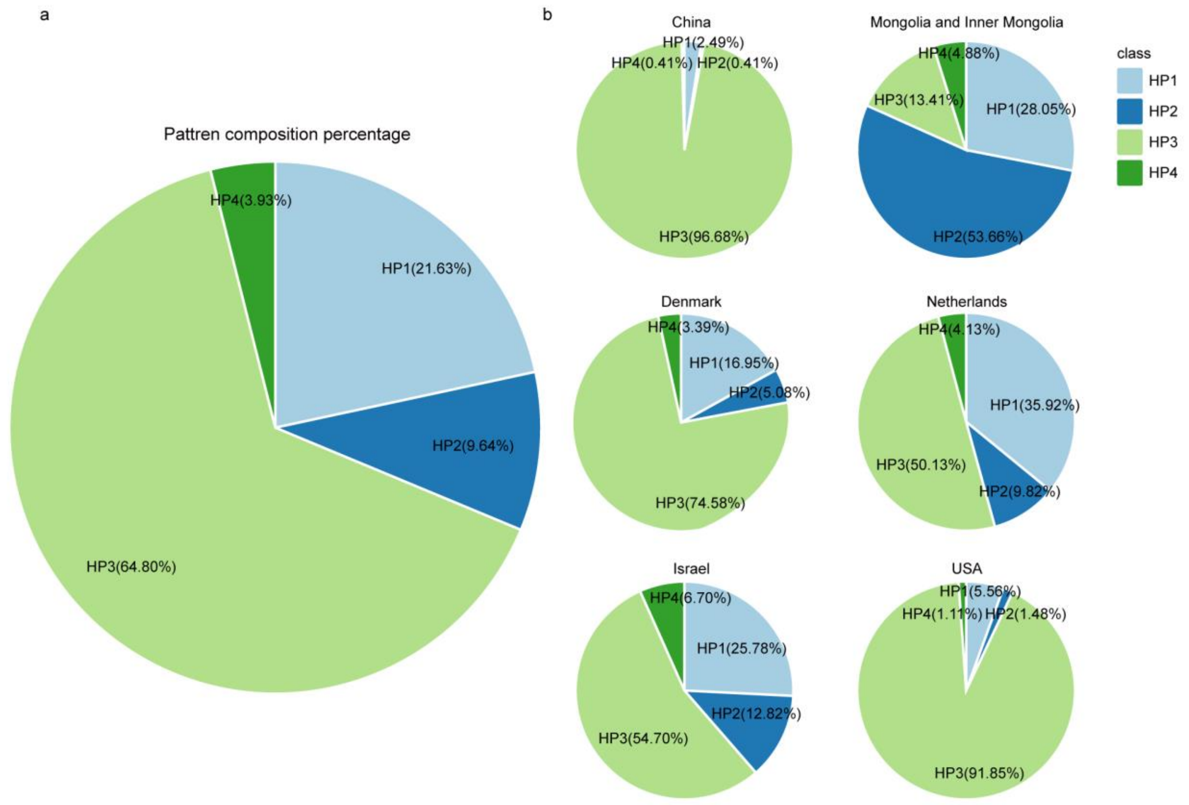


**Figure S1. The proportion of four healthy microbial composition patterns. (a)** An overview of healthy patterns involved in healthy populations. The total number of healthy samples is 1,909. Among them, 413 samples make up the first pattern (HP1), 184 samples constitute the second pattern (HP2), 1,237 samples constitute the third pattern (HP3), and 75 are left for the fourth pattern (HP4). **(b)** Healthy pattern proportions across different regional cohorts in the dataset. Regional cohorts with more than 50 healthy samples after hiPCA filtering were considered. Accordingly, six cohorts, namely China (241 samples), Denmark (59 samples), Israel (702 samples), Mongolian and Inner Mongolian (82 samples), Netherlands (387 samples), and USA (270 samples) were displayed under this perspective.

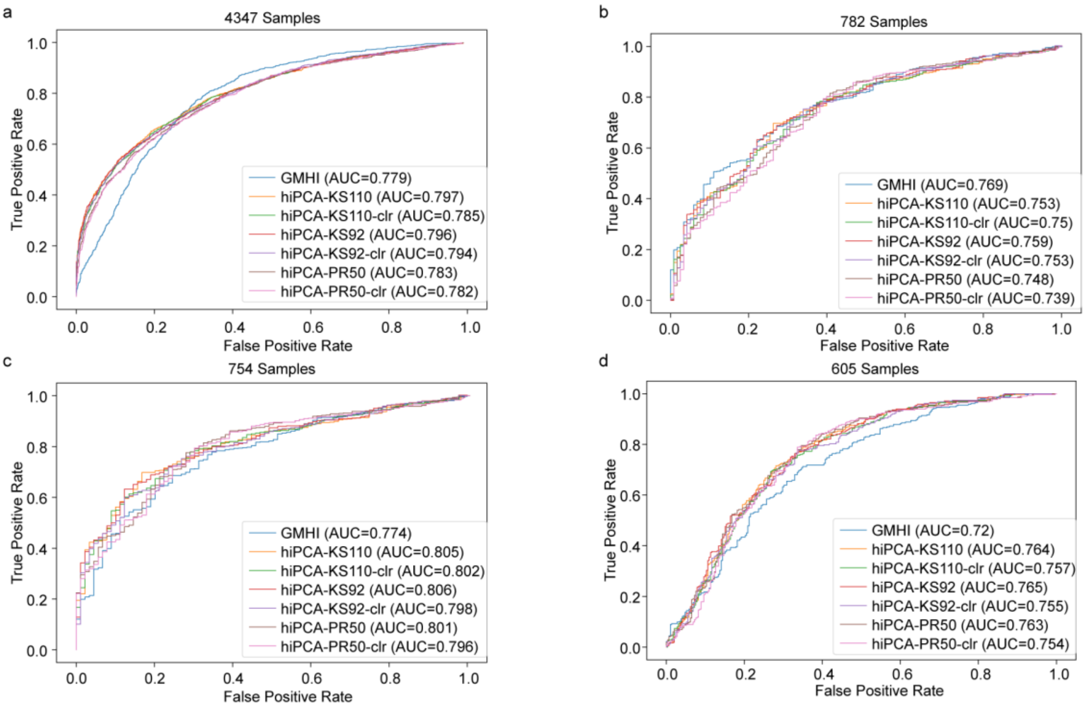


**Figure S2. AUC plots for (a)** all 4347 samples in training data **(b)** all 782 samples in validation data **(c)** all 754 samples in validation data (excluding 28 'healthy' questionable samples from the Parkinson cohort) **(d)** all 605 samples in testing data. hiPCA-KS110 stands for the hiPCA modeling with 110 features, while the 'clr' stands for the Centered Log-Ratio transformation.

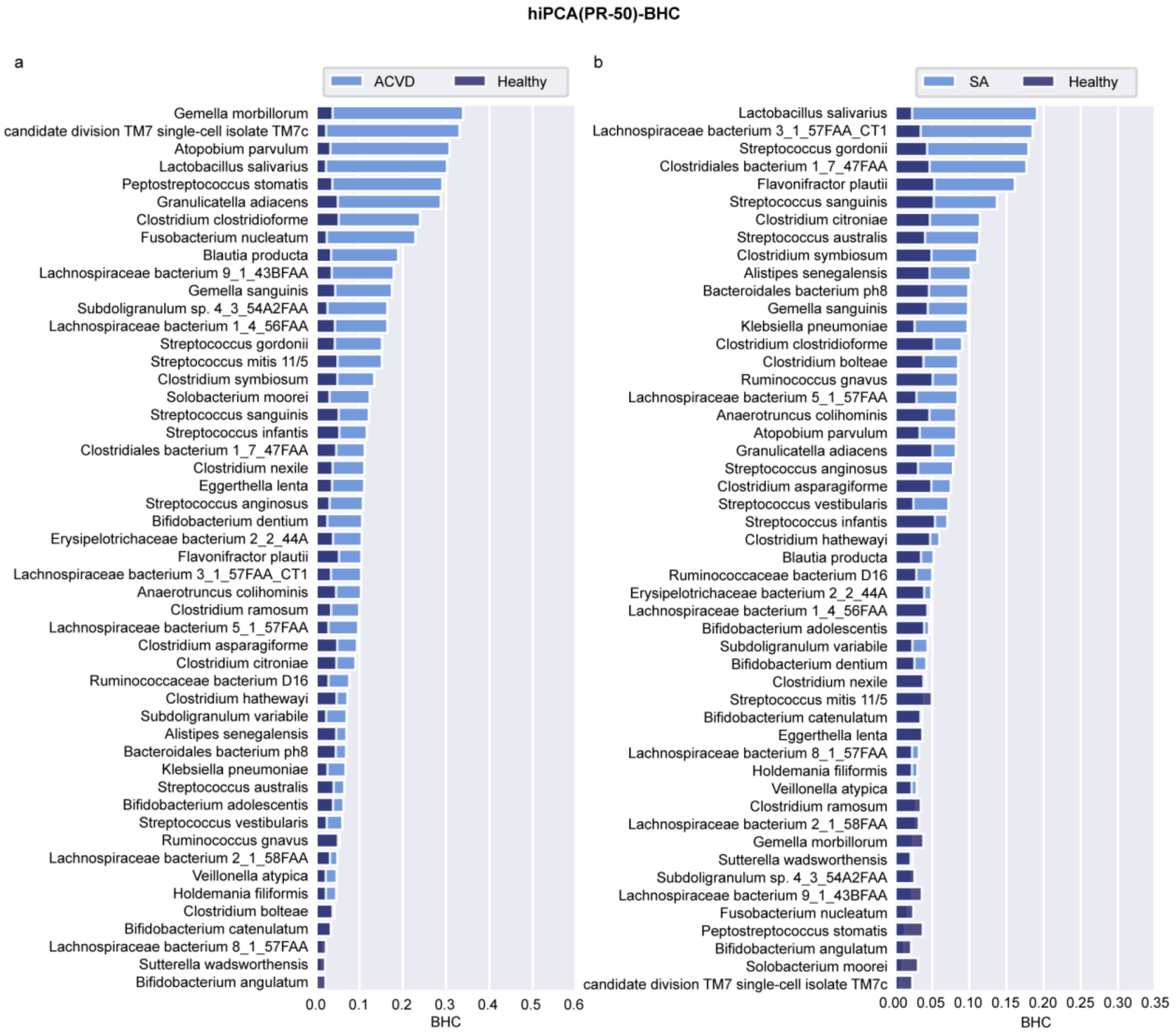


**Figure S3. BHC bar plot for the healthy and two disease subgroups under PR-50 features.** The background dark blue bar denotes the population-averaged BHC in the healthy subgroup, and the light blue bar represents the population-averaged BHC in the disease subgroup. **(a)** ACVD **(b)** SA.

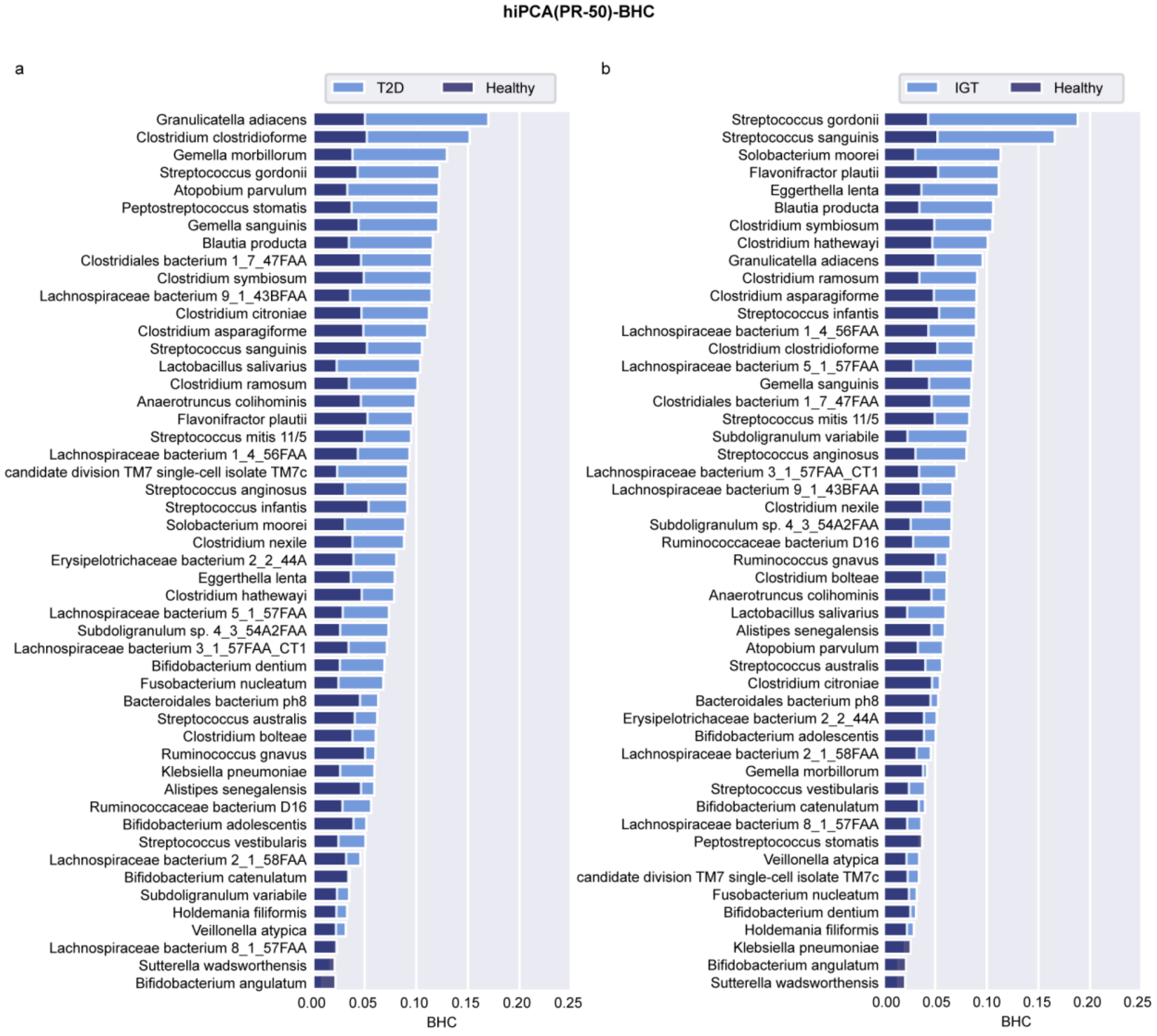


**Figure S4. BHC bar plot for the healthy and two disease subgroups under PR-50 features. (a)** T2D **(b)** IGT.

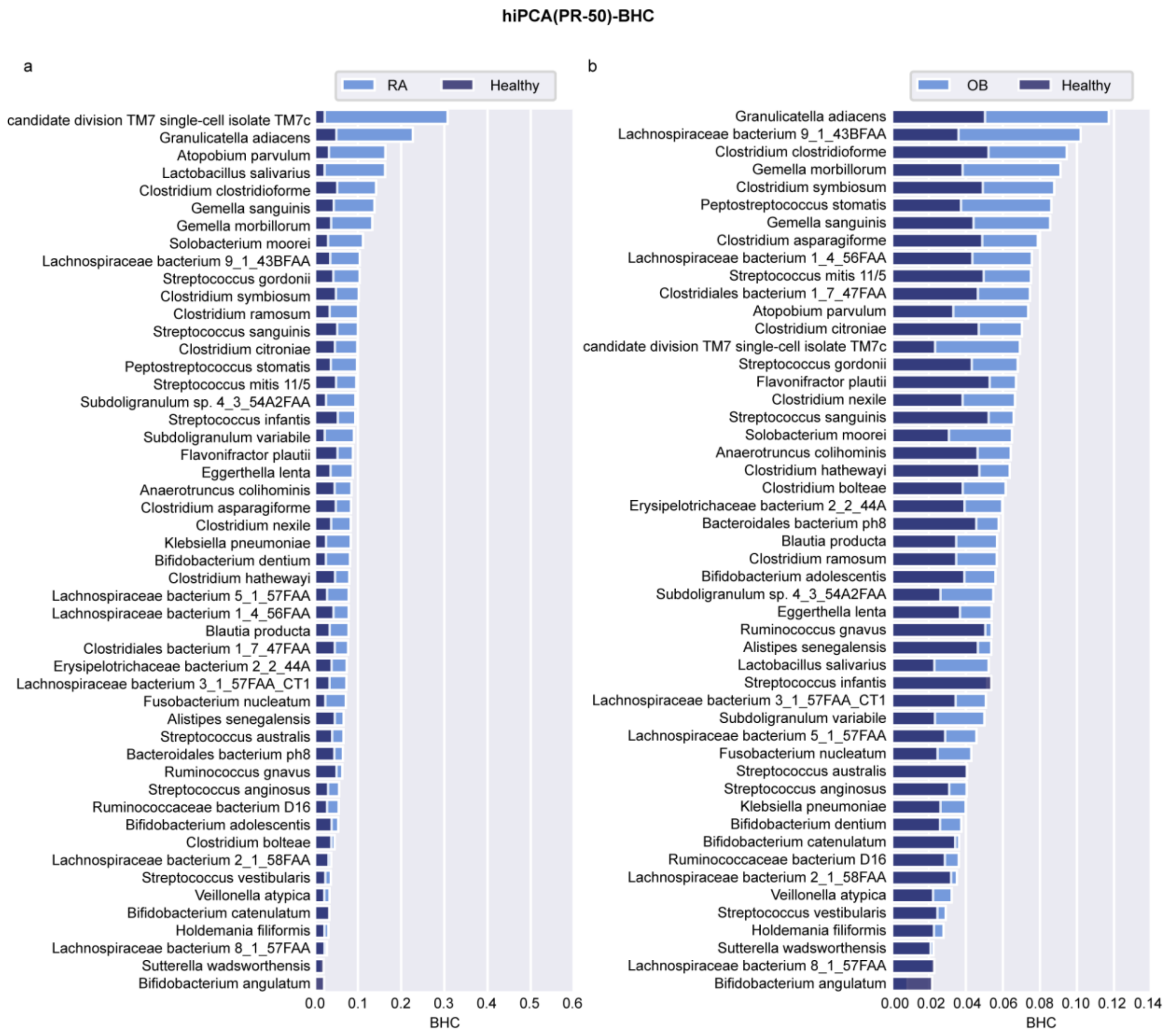


**Figure S5. BHC bar plot for the healthy and two disease subgroups under PR-50 features. (a)** RA **(b)** OB.

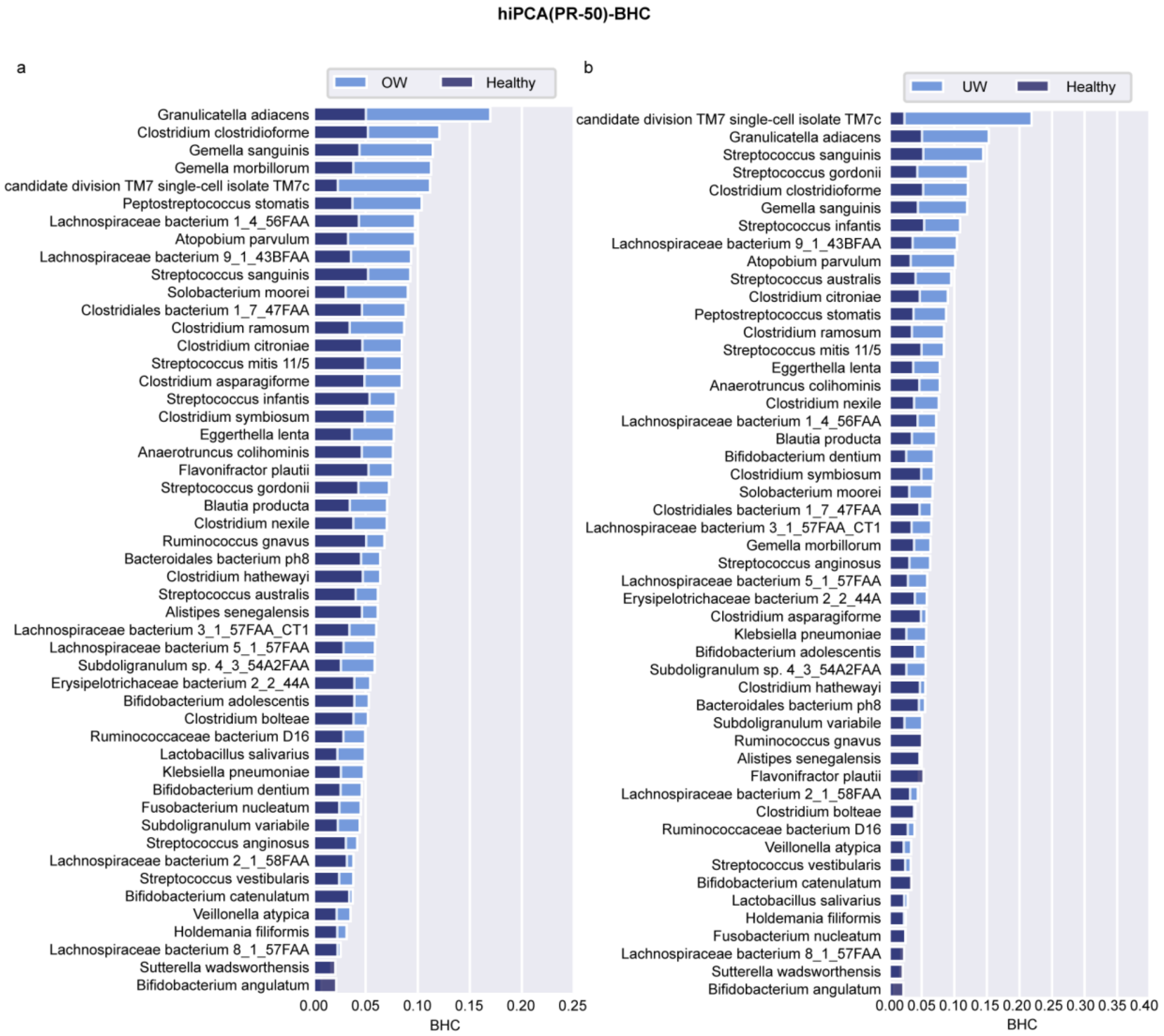


**Figure S6. BHC bar plot for the healthy and two disease subgroups under PR-50 features. (a)** OW **(b)** UW.

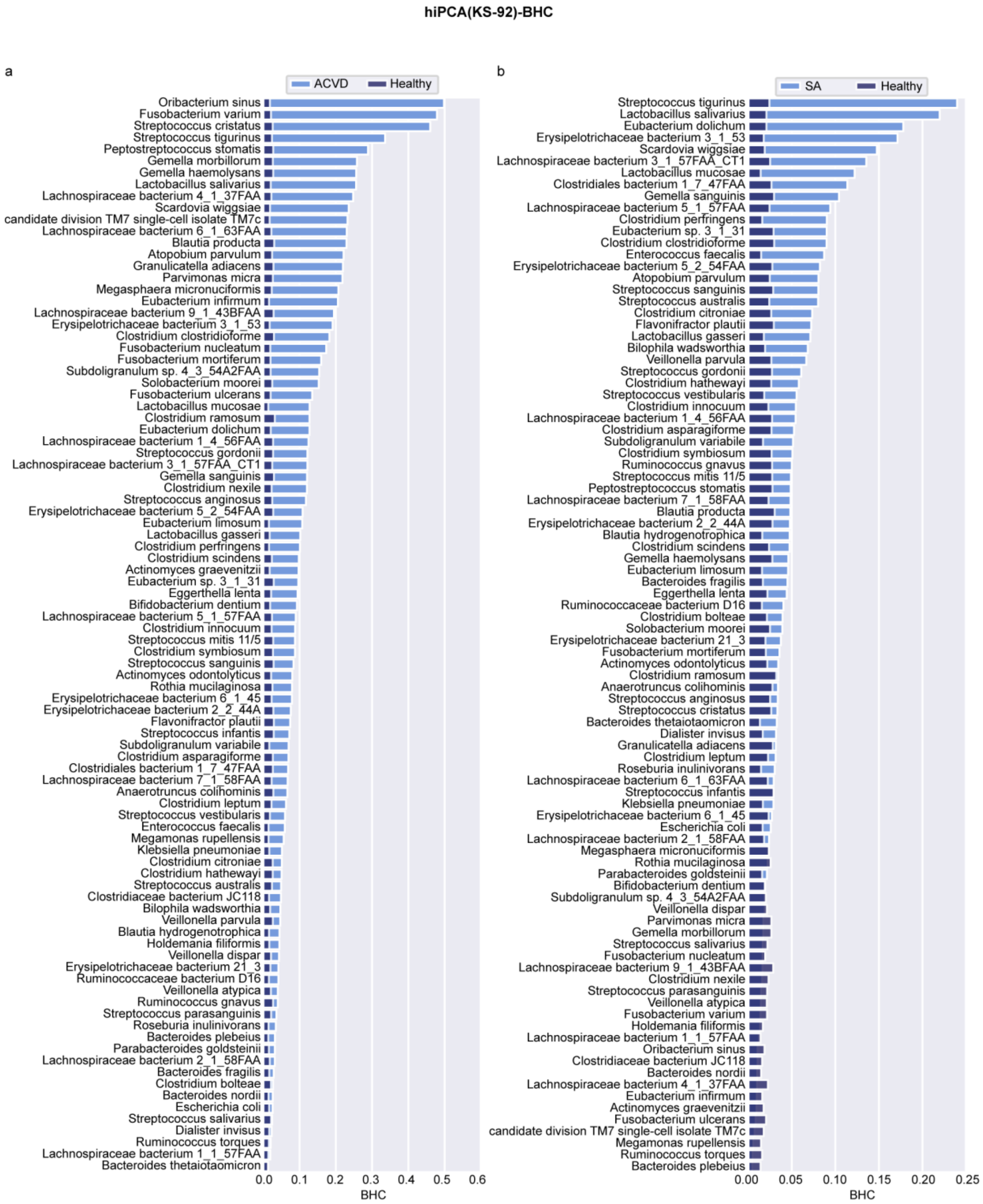


**Figure S7. BHC bar plot for the healthy and two disease groups under KS 92 features. (a)** ACVD, **(b)** SA.

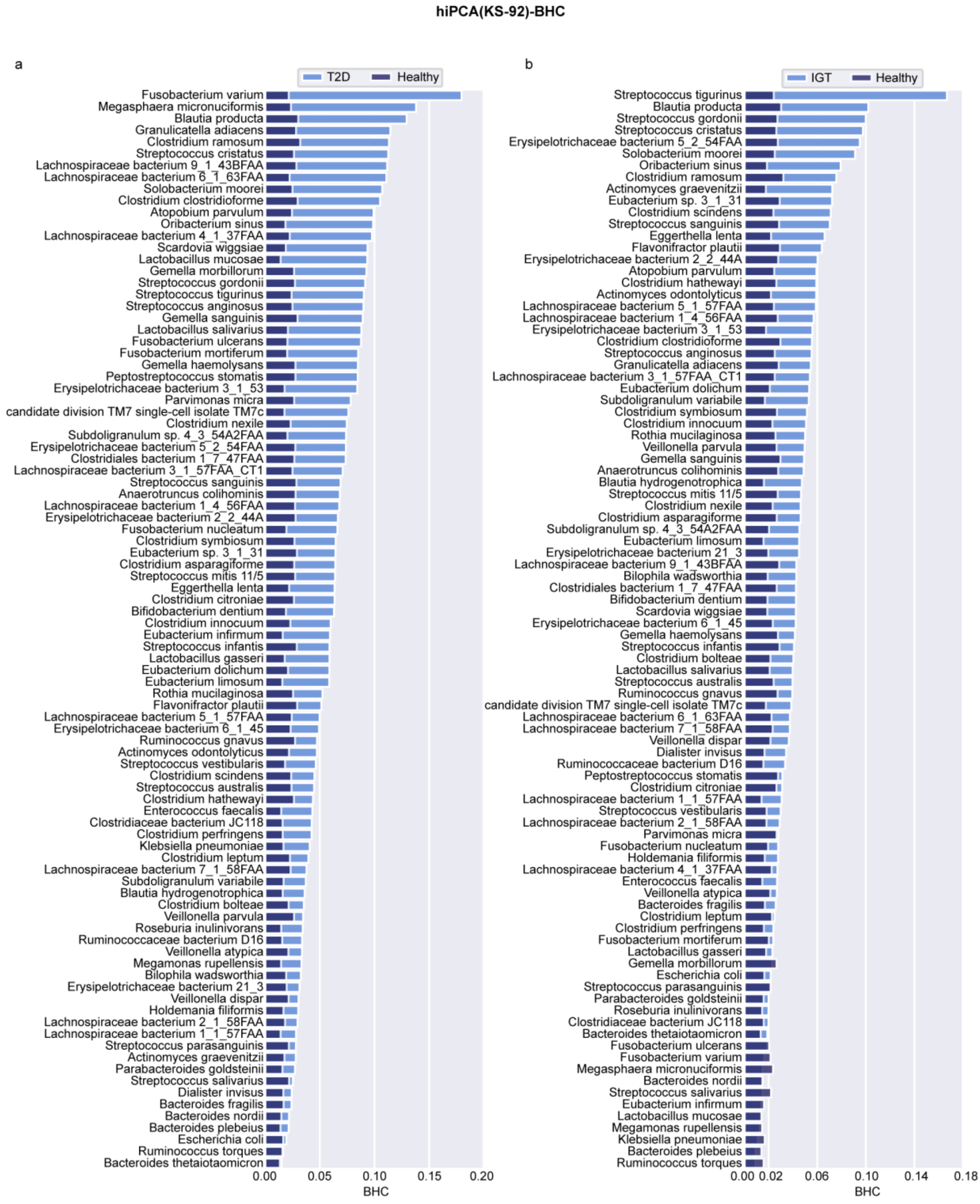


**Figure S8. BHC bar plot for the healthy and two disease groups under KS-92 features. (a)** T2D **(b)** IGT.

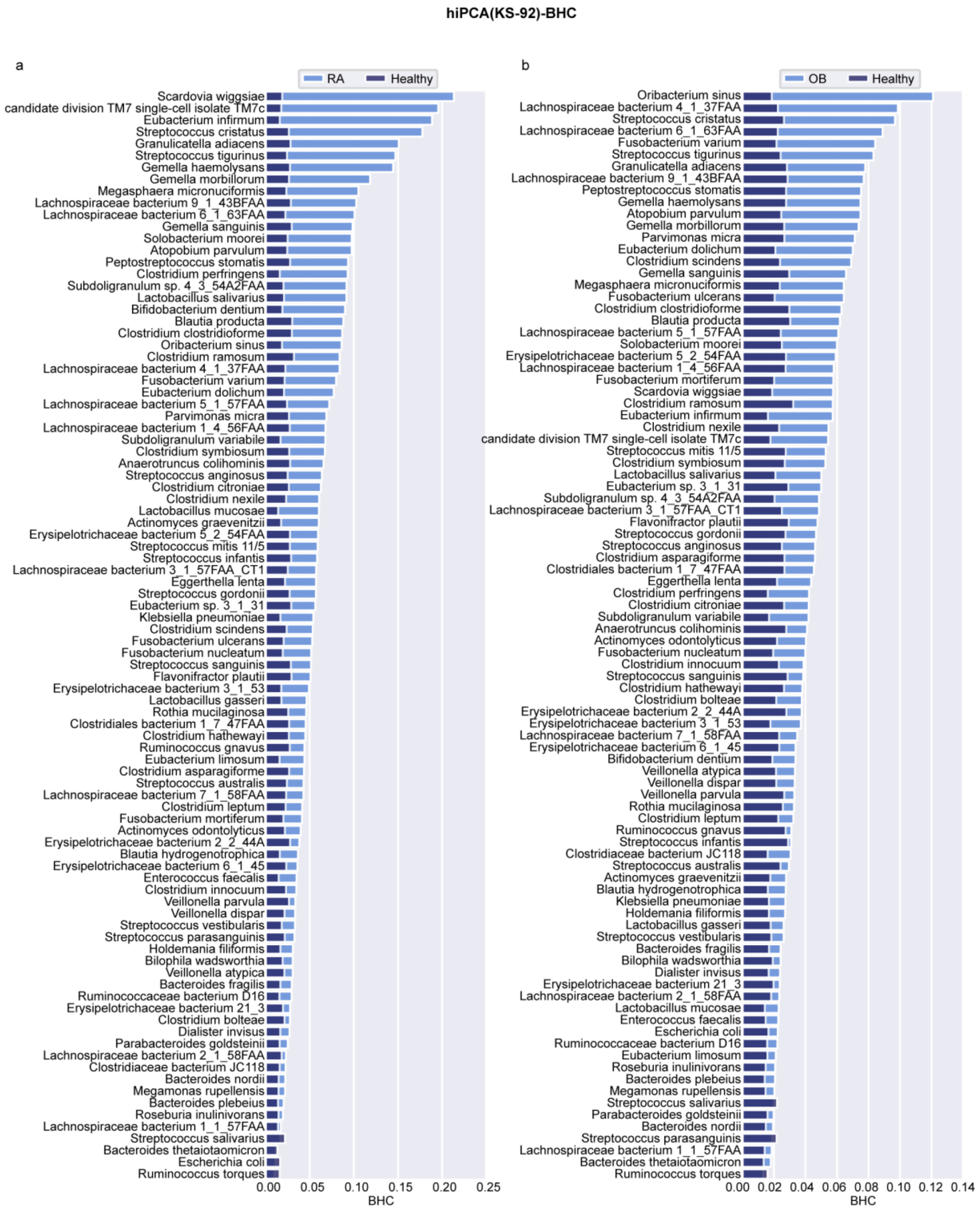


**Figure S9. BHC bar plot for the healthy and two disease groups under KS-92 features. (a)** RA **(b)** OB.

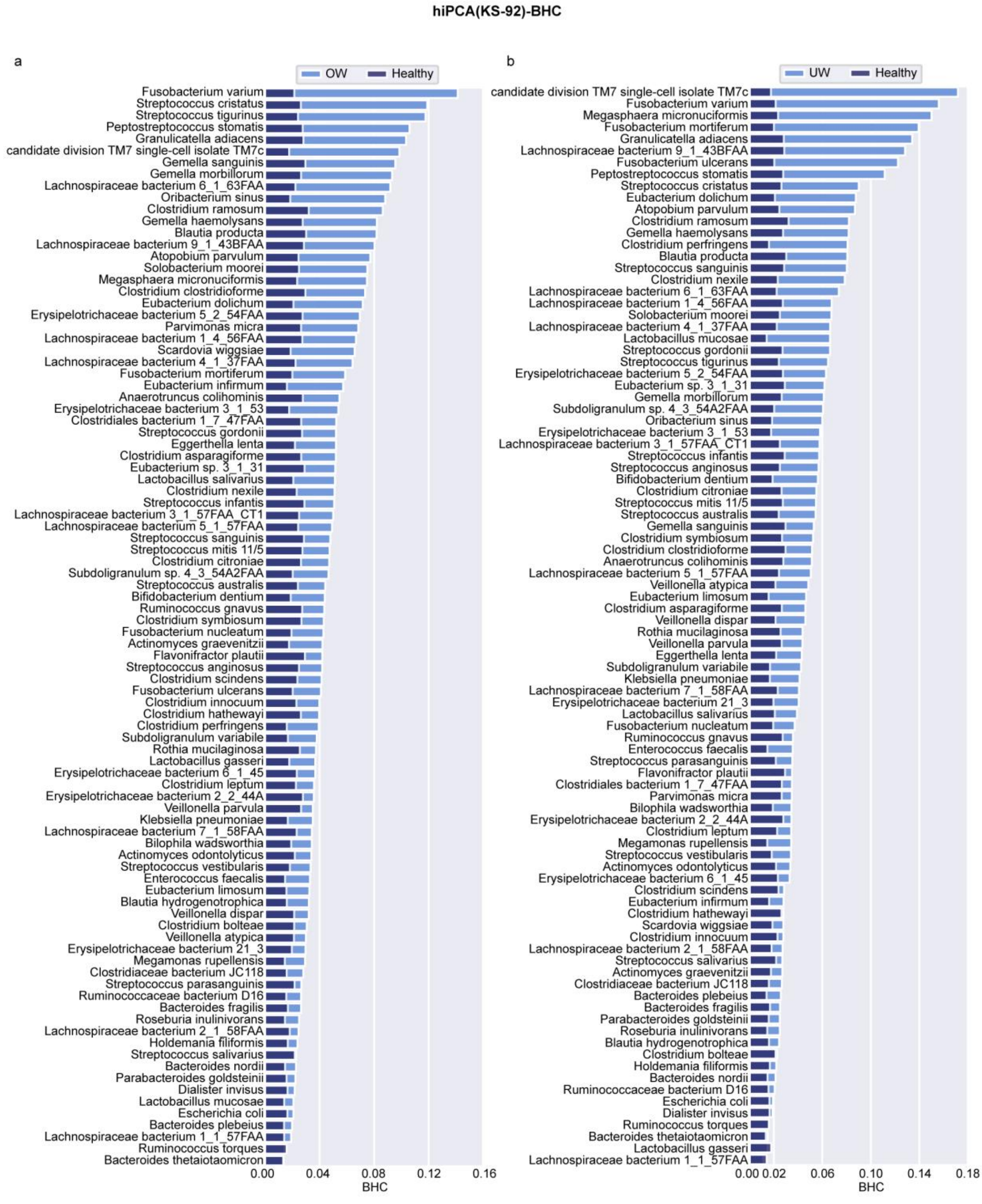


**Figure S10. BHC bar plot for the healthy and two disease groups under KS 92 features. (a)** OW **(b)** UW.

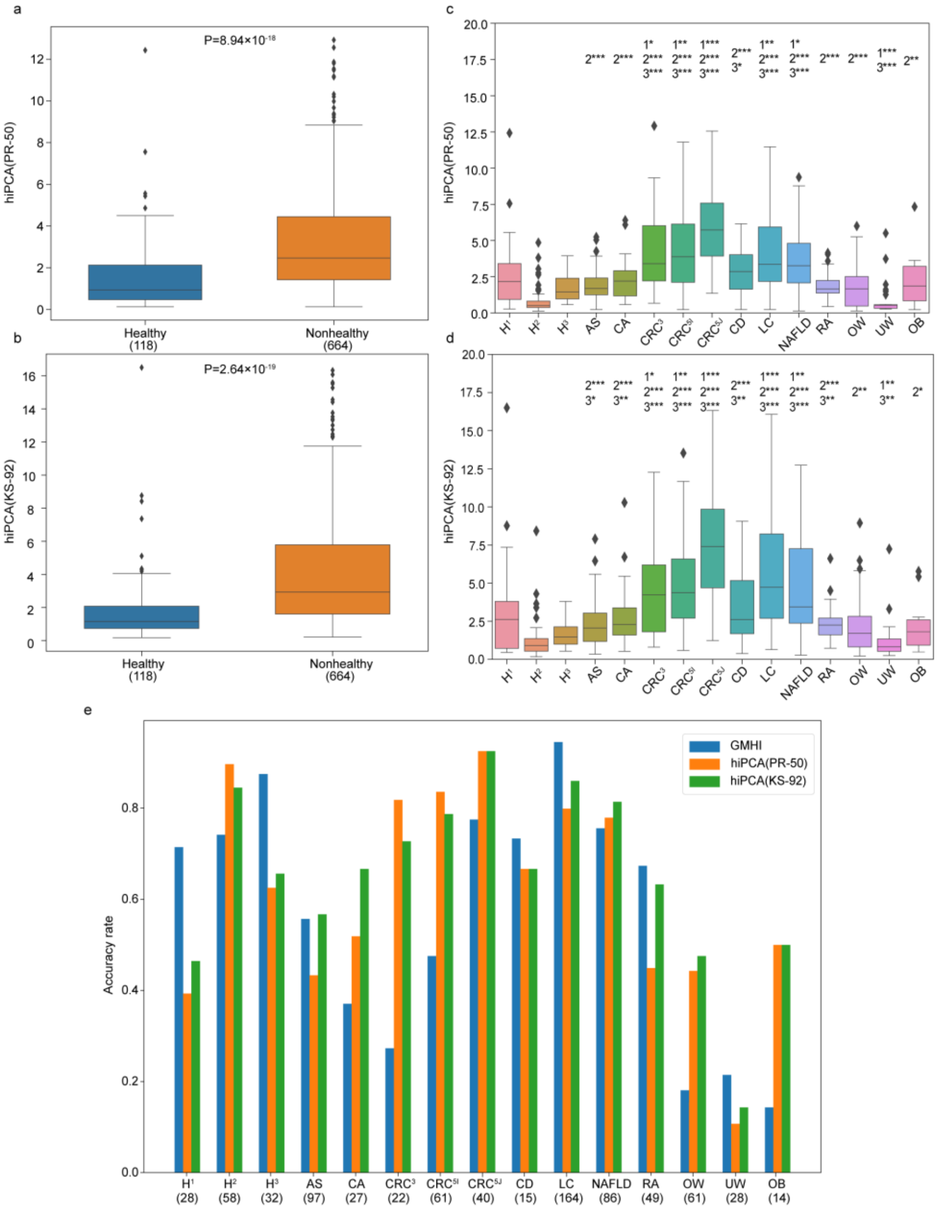


**Figure S11. The hiPCA demonstrates strong reproducibility on the additional validation data.** **(a-b)** Distribution of hiPCA (PR-50 and KS-92) in healthy cohorts and nonhealthy cohorts. **(c-d)** Distributions of hiPCA (PR-50 and KS-92) in three healthy sub-cohorts and 12 nonhealthy sub-cohorts. **(e)** The hiPCA and GMHI accuracy rates over different cohorts. All *P*-values shown above the box plots are found using the two-sided Mann-Whitney U test: *, $P \leq 0.05$; **, $P \leq 0.01$; ***, $P \leq 0.001$; ns, not significant. The sample size of each cohort is shown within parentheses. Among all healthy cohorts, only the H2 cohort explicitly claimed healthy.

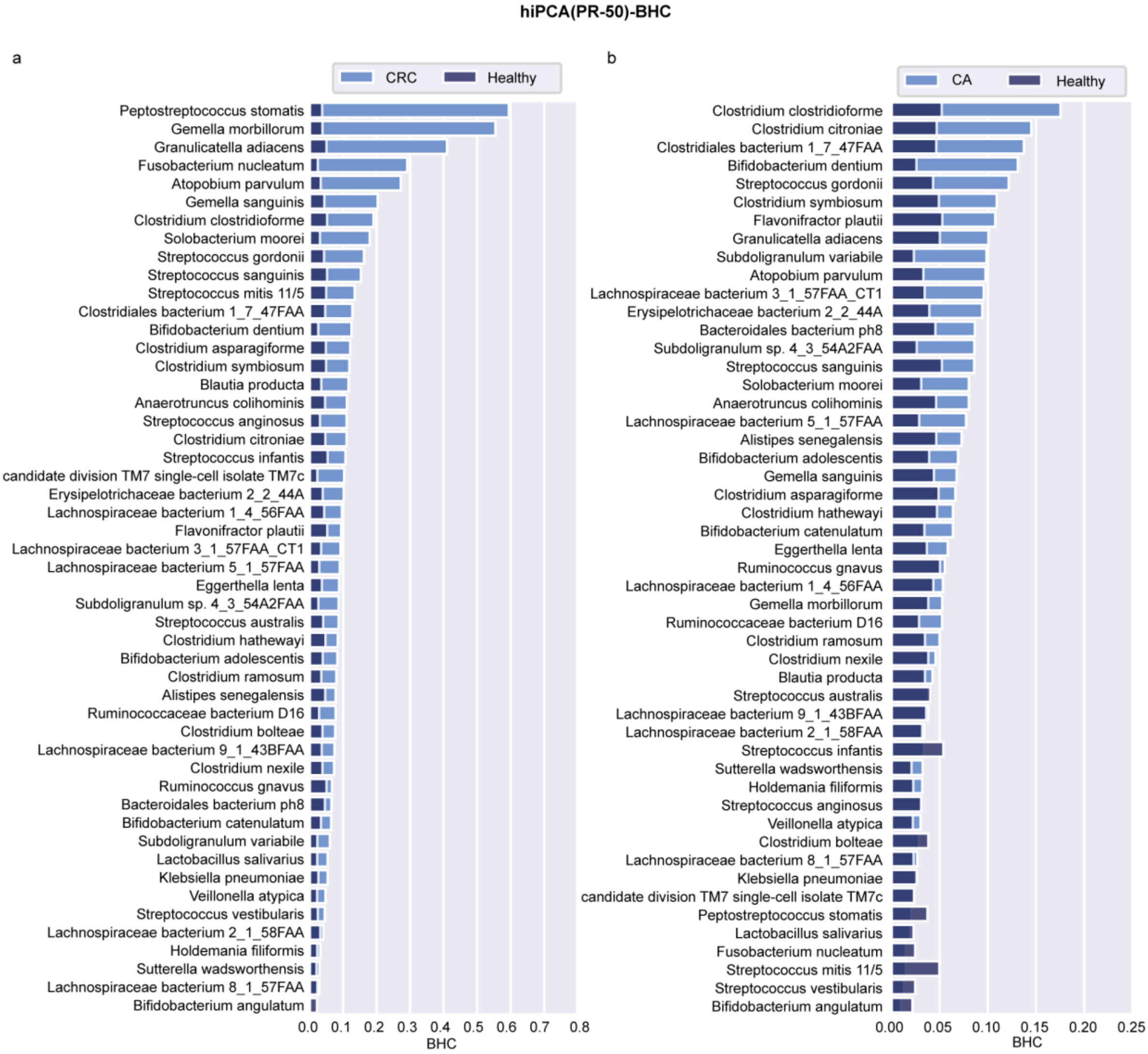


**Figure S12. BHC bar plots for the CRC/CA sub-groups using PR-50 features. (a)** CRC **(b)** CA.

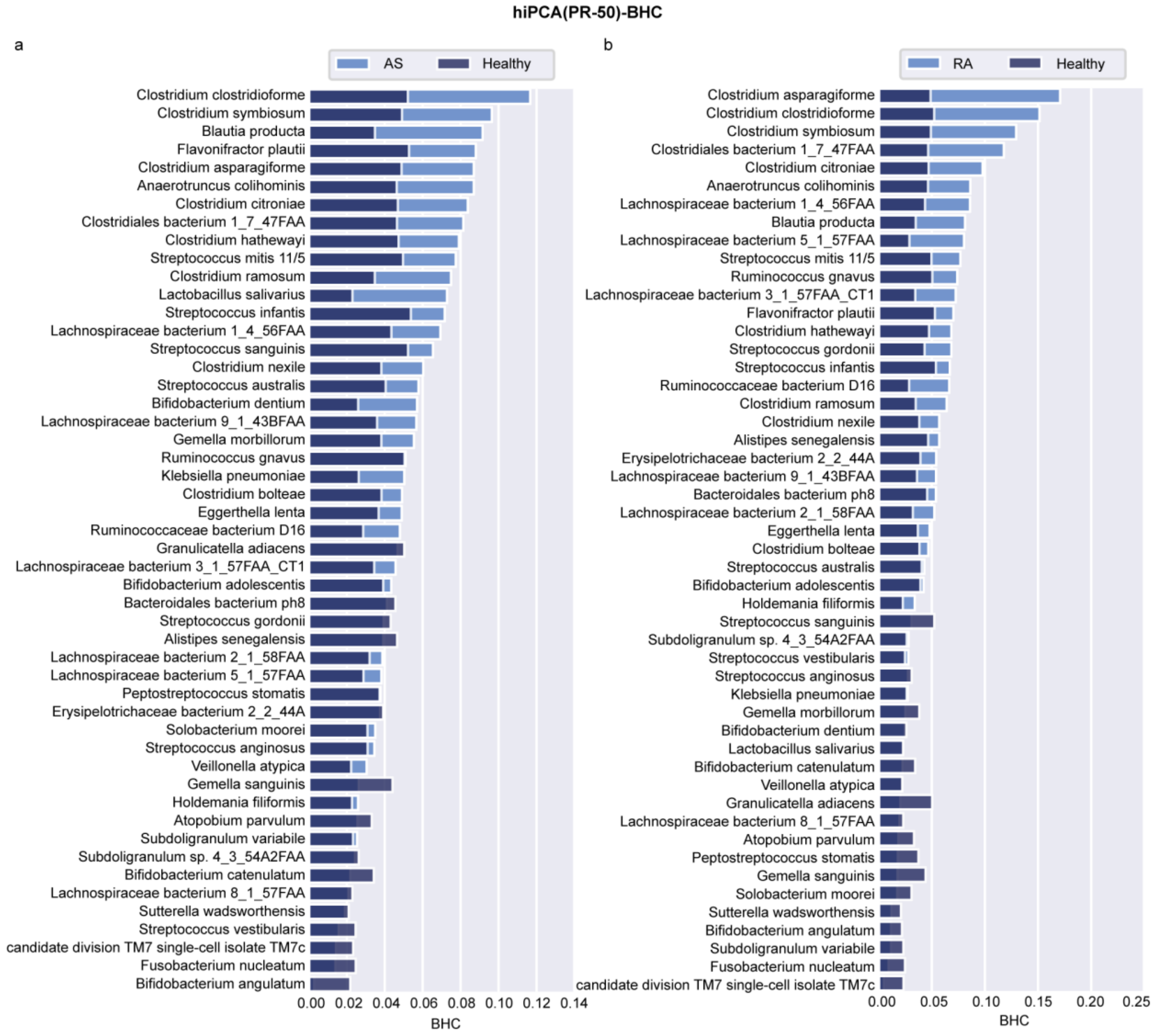


**Figure S13. BHC bar plots for the AS/RA sub-groups using PR-50 features. (a)** AS **(b)** RA.

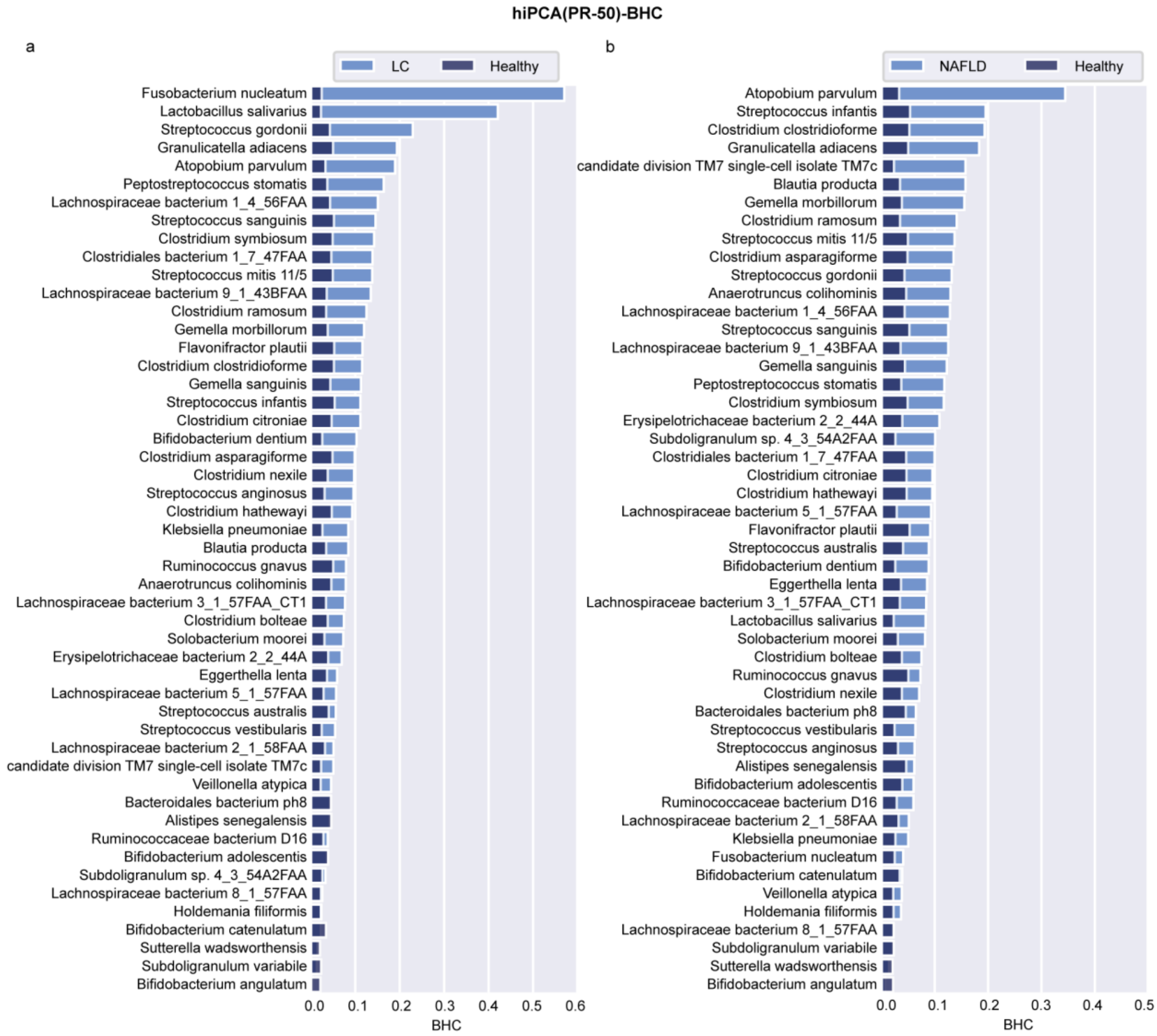


**Figure S14. BHC bar plots for the LC/NAFLD sub-groups using PR-50 features. (a)** LC **(b)** NAFLD.

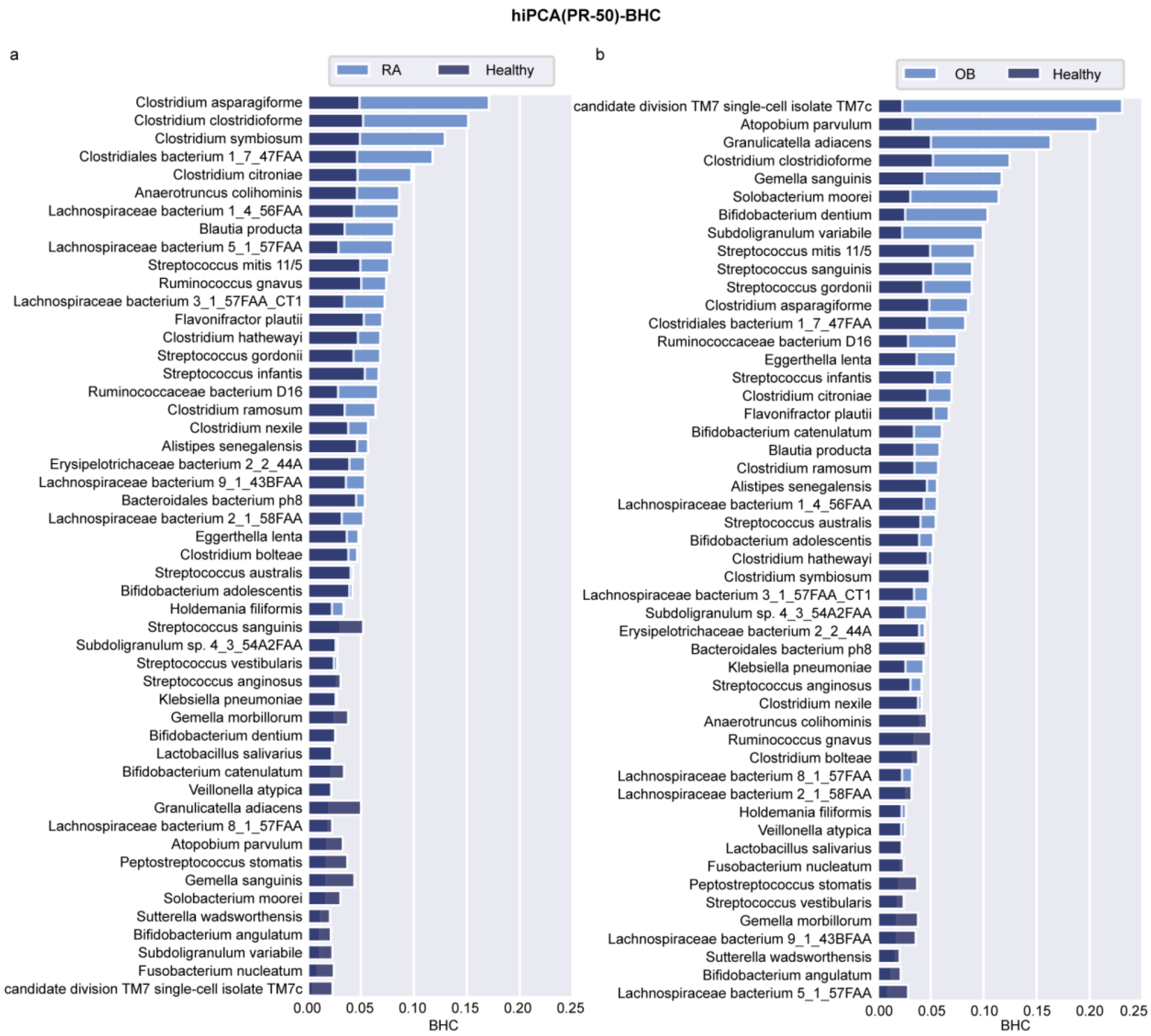


**Figure S15. BHC bar plots for the RA/OB case groups using PR-50 features**. **(a)** RA **(b)** OB.

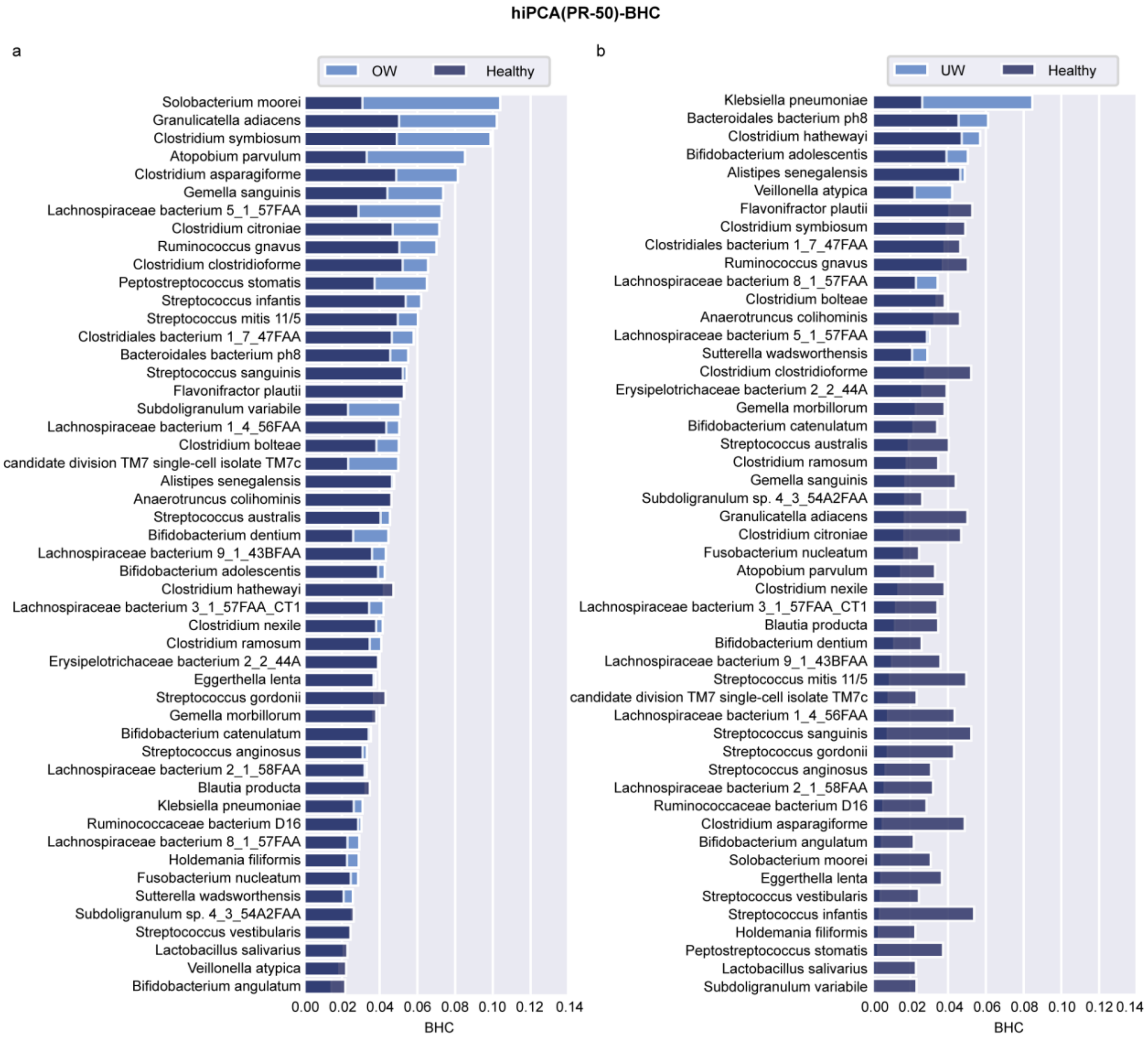


**Figure S16. BHC bar plots for the OW/UW case groups using PR-50 features**. **(a)** OW **(b)** UW.

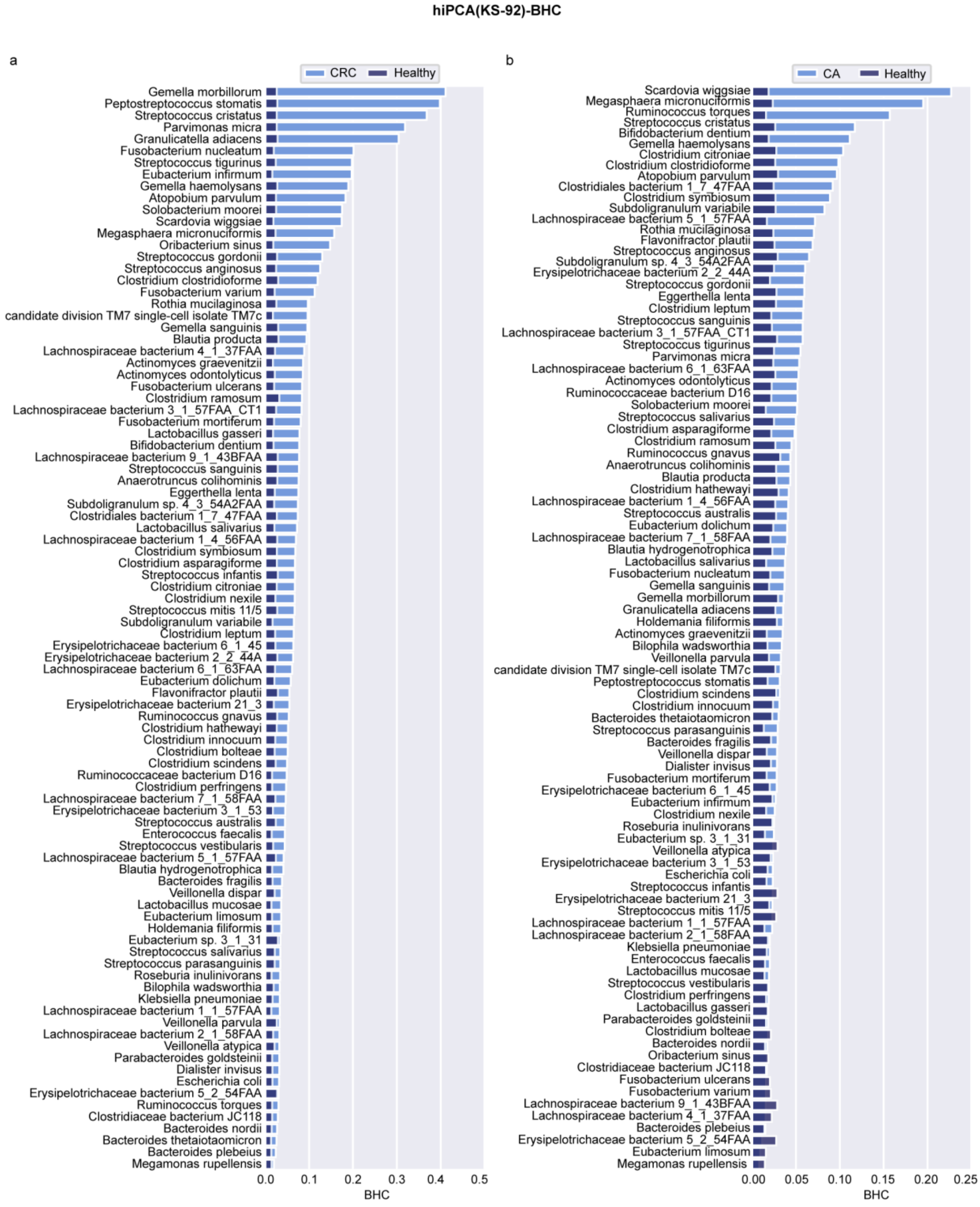


**Figure S17. BHC bar plots for the CRC and CA case groups using KS-92 features. (a)** CRC **(b)** CA.

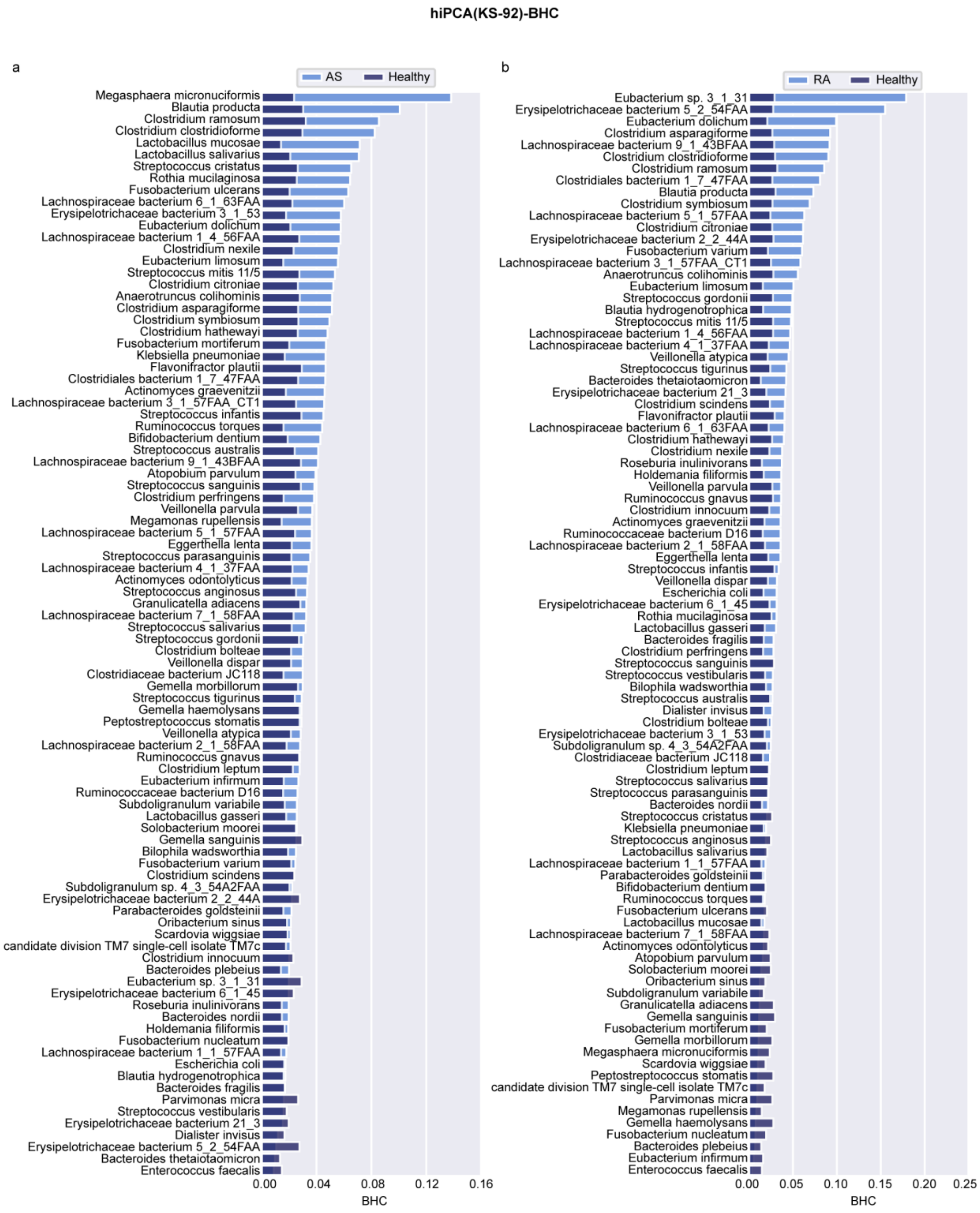


67

68 **Figure S18. BHC bar plots for the AS and RA sub-groups using KS-92 features. (a)** AS **(b)** RA.

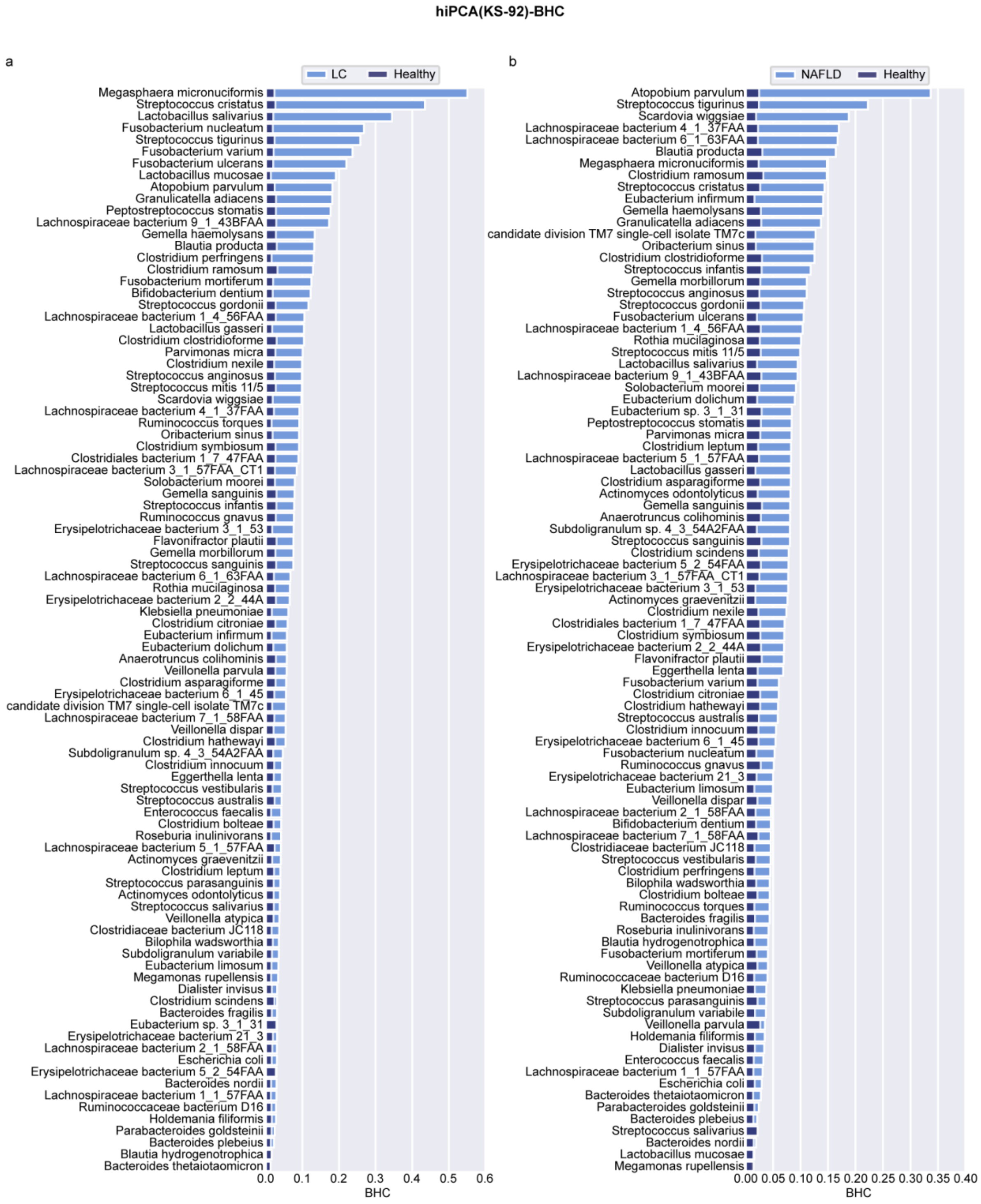


**Figure S19. BHC bar plots for the LC and NAFLD subgroups using KS-92 features. (a)** LC **(b)** NAFLD.

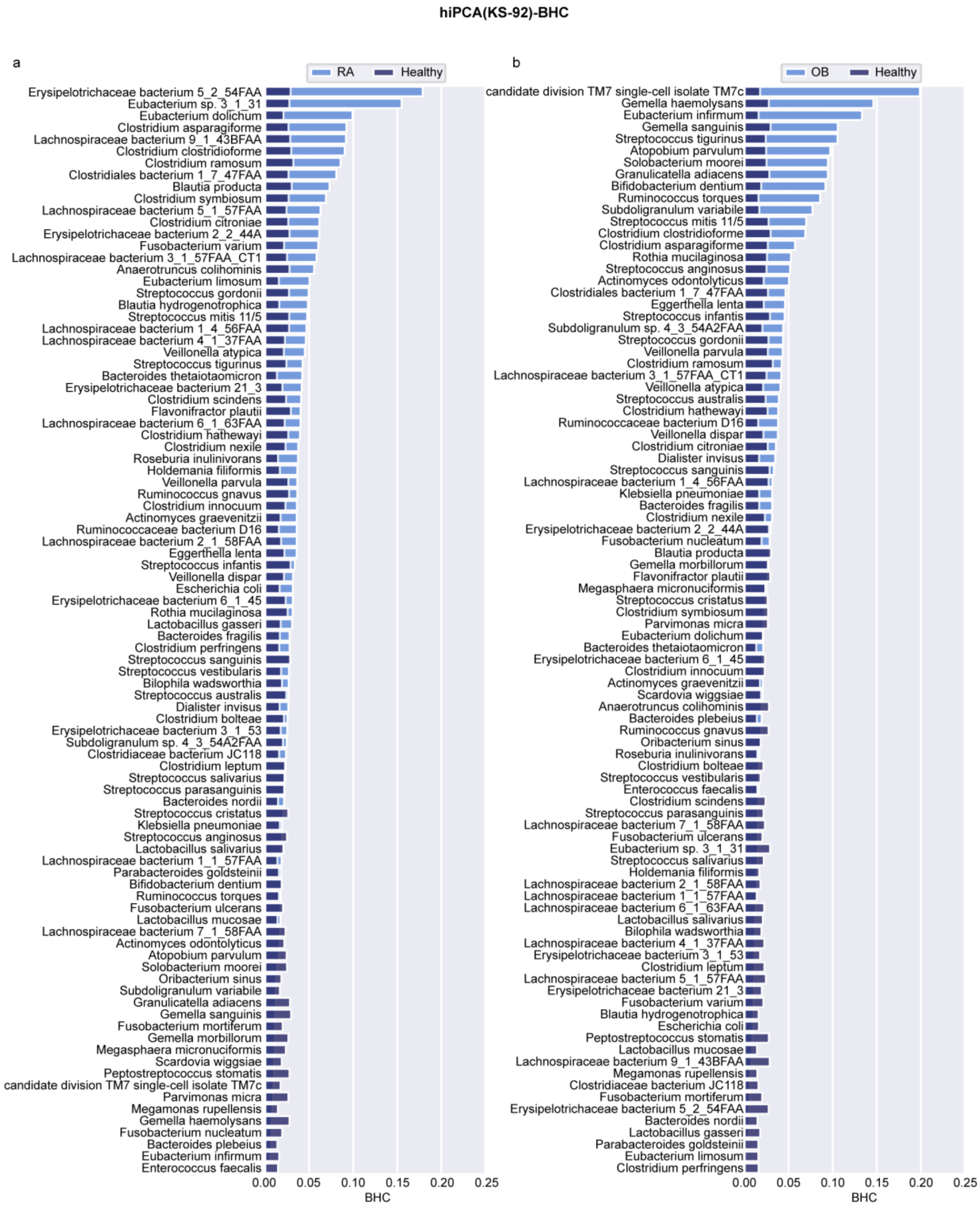


**Figure S20. BHC bar plots for the RA and OB case groups using KS-92 features. (a)** RA **(b)** OB.

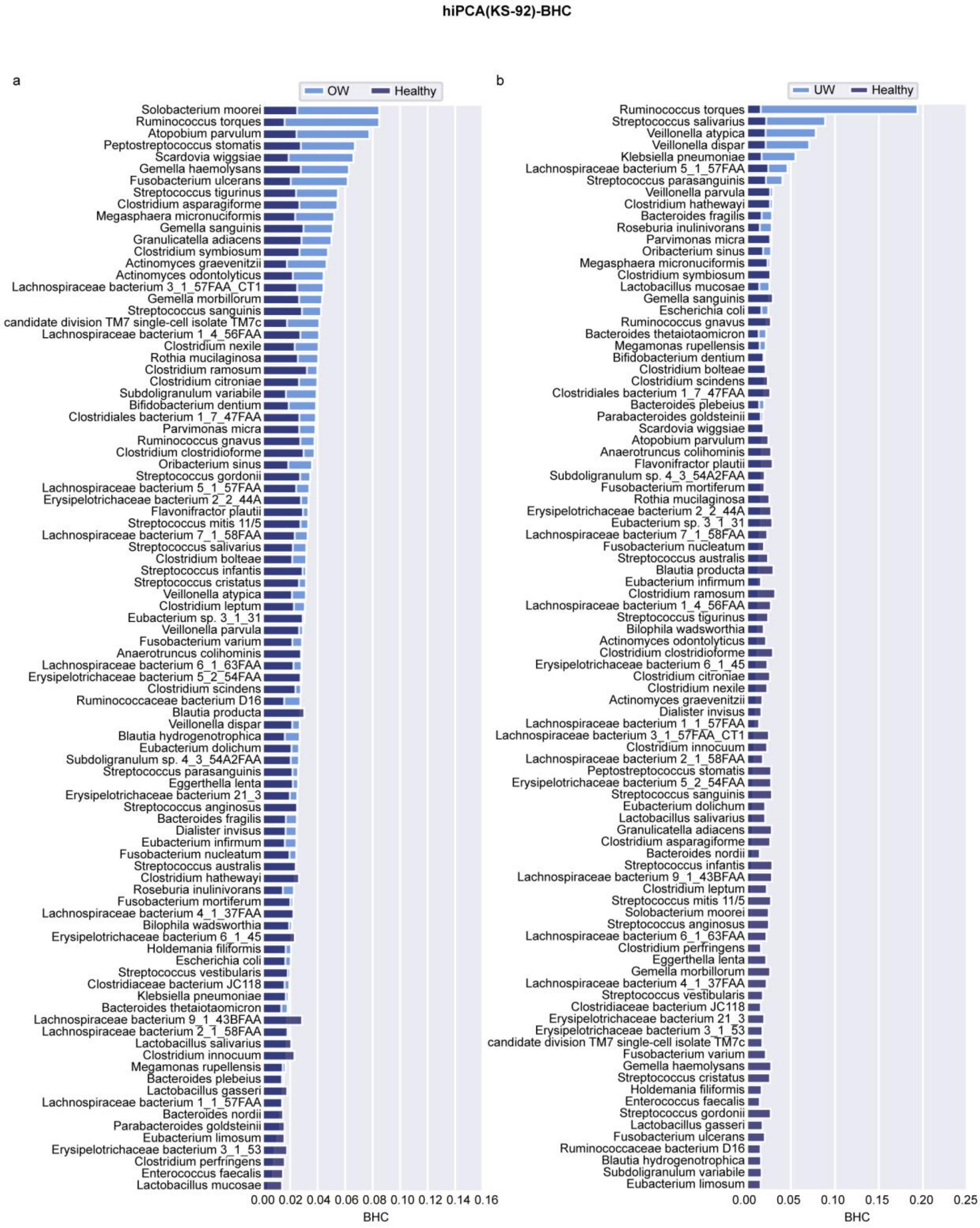


**Figure S21. BHC bar plots for OW and UW groups using KS-92 features. (a)** OW **(b)** UW.

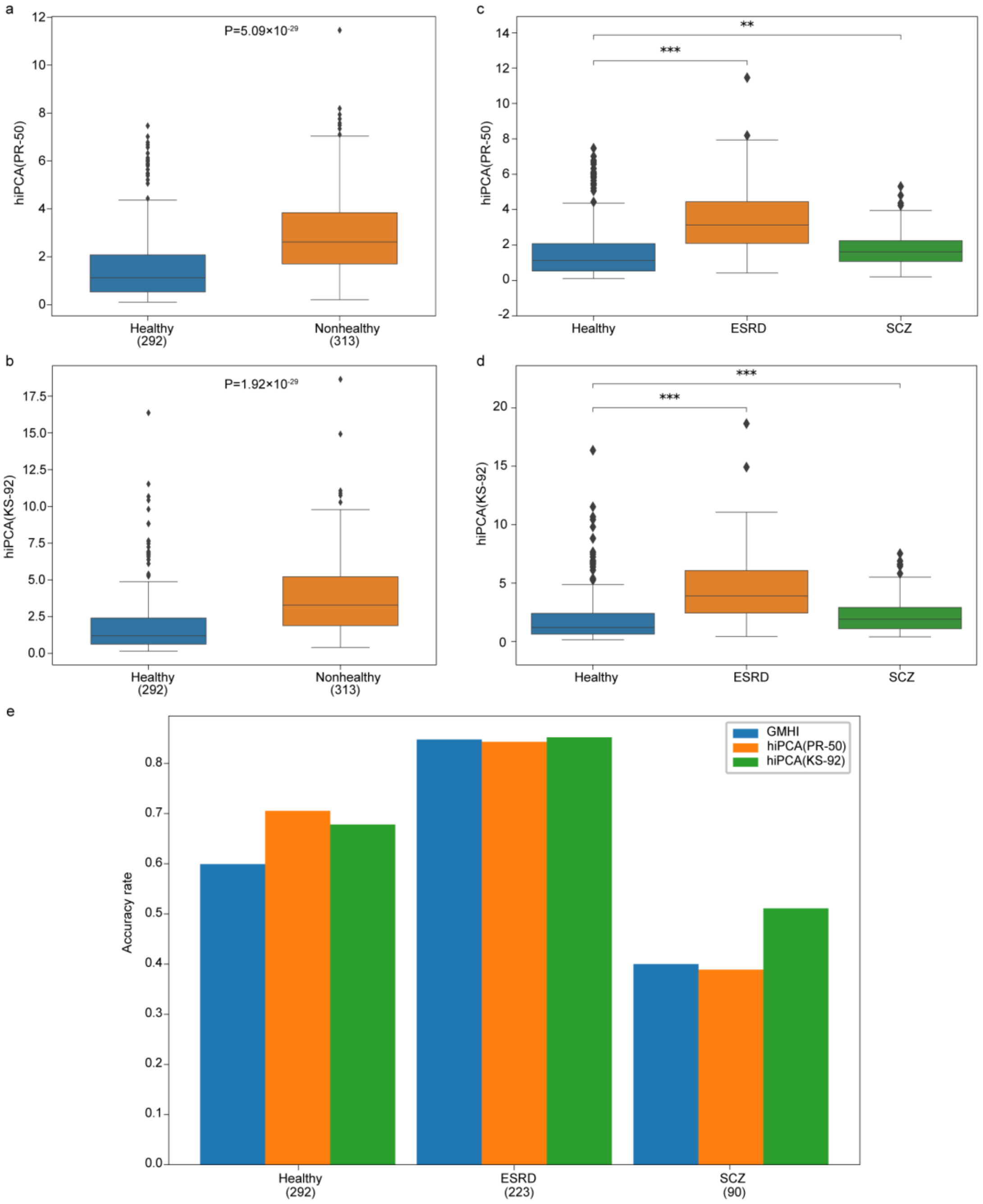


**Figure S22. The hiPCA shows consistent results on the test dataset. (a-b)** Distribution of hiPCA (PR-50 and KS-92) in healthy cohorts and nonhealthy cohorts. **(c-d)** Distributions of hiPCA (PR-50 and KS-92) in the healthy cohort and two nonhealthy sub-cohorts. **(e)** The hiPCA and GMHI accuracy rates over different cohorts. All *P*-values shown above the box plots are found using the two-sided Mann-Whitney U test: *, $P \leq 0.05$; **, $P \leq 0.01$; ***, $P \leq 0.001$; ns, not significant. The sample size of each cohort is shown within parentheses.

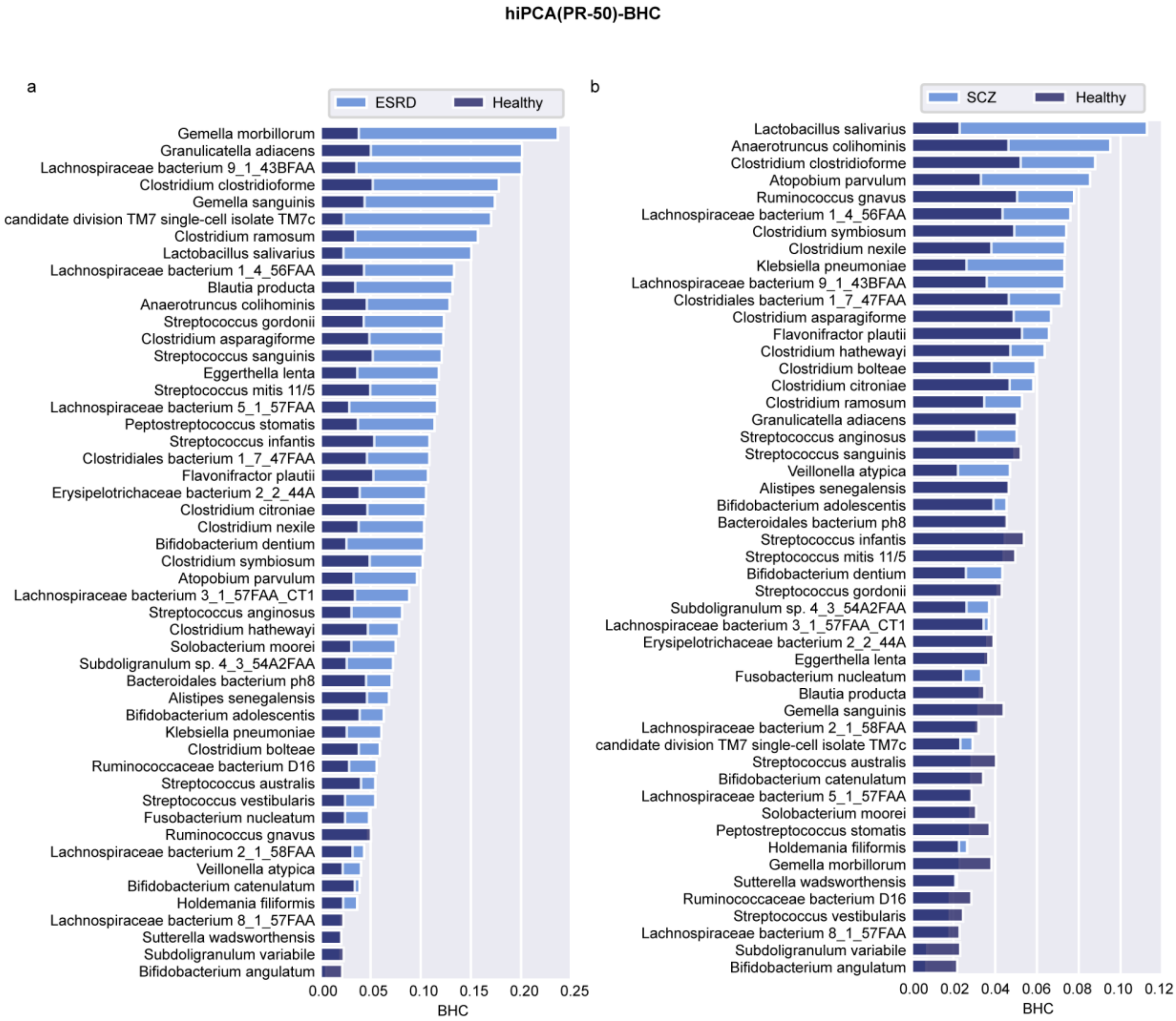


**Figure S23. BHC bar plots for ESRD and SCZ groups using PR-50 features. (a)** ESRD **(b)** SCZ.

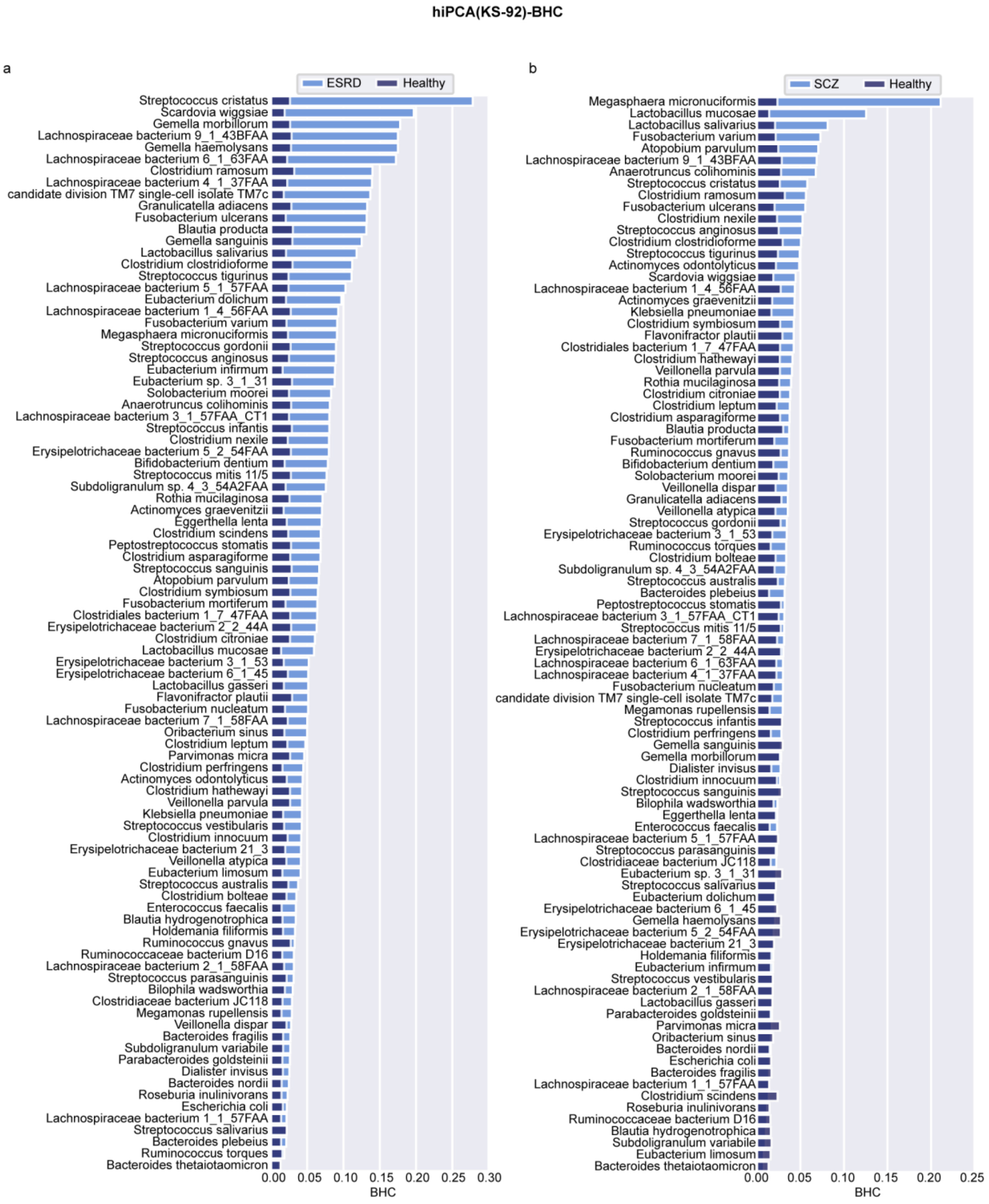


**Figure S24. BHC bar plots for ESRD and SCZ groups using KS-92 features. (a)** ESRD **(b)** SCZ.
