# Supplementary material for: Statistical modeling of gut microbiota for personalized health status monitoring
Source: Microbiome. 2023 Aug 18;11:184. doi: 10.1186/s40168-023-01614-x (PMC10436630; doi:10.1186/s40168-023-01614-x)
Supplement: Supplementary file 5 — Additionalfile 4. [file 40168_2023_1614_MOESM4_ESM.pdf]

# Supplementary Note 1. Additional analysis of four healthy patterns

Relations and differences with enterotypes. It should be emphasized that our definition of the healthy pattern was different from the previous concept and research on enterotypes, where the driver taxon analysis typically comes out with a total of three types [1, 2]: ET-B (*Bacteroides*-driven), ET-P (*Prevotella*-driven), and ET-F (*Ruminococcus*). It was argued that enterotypes could be variated in geographical regions and correlate with diet/lifestyle/health status, which in term could become an ambiguous indicator for describing the consistency of community taxa [3]. Different studies may also come out with partially inconsistent conclusions. For example, a recent inter-continental comparison study marked *Prevotella*, *Bacteroides*, and *Bifidobacterium* as the key enterotypes [4]. In fact, the hiPCA will boost the separation of healthy patterns by first eliminating those potentially unhealthy collections, which in terms could leverage a more distinct stratification of healthy gut community structures for useful clinical reference in practice. To explore the discriminative ability of four patterns, we further built the random forest classifier by using 66 species from six main genera as features and health patterns as labels. As shown in Table S4, the AUC can be over 99% which implies that there exist significant patterns in the population, and our division is reasonable in pattern recognition. Interestingly, we found *Bifidobacterium* contributed primarily to classification performance, and using the species features was found to perform over 99% of AUC. As a comparison with enterotype, we first divided enterotypes for 1909 health samples using an enterotype classification model trained with metaHIT and HMP [2]. A total of 1532 samples looked similar to the reference dataset and were labeled with ET-P, ET-B, or ET-F. Then the labeled enterotypes were reused as labels for random forest analysis. It was found that the RF could obtain the AUC over 98% with genus *Bacteroides* and *Prevotella*. After further investigating fine-grained species level, RF could

achieve AUC over 90% on enterotype classification using *Prevotella copri* and three *Bacteroides species* (*B. uniformis*, *B. vulgatus*, and *B. stercoris*). We could conclude that the discrimination of our health patterns was mostly driven by *Bifidobacterium* while the previous enterotypes were mainly discriminated by *Bacteroides* and *Prevotella*.

Regional comparison. To investigate the interregional differences in healthy patterns, regional factor was considered, including China, Denmark, Israel, Mongolian and Inner Mongolian, Netherlands, and USA (Figure S1). It was revealed that for USA and China cohorts, both were dominated by HP3 that exhibited the significant microbial richness of *Bacteriodes*, we speculate that both cohorts should be characterized by the western dietary patterns. The Mongolian and Inner Mongolian cohort was dominated by HP2, and the enriched species were *Faecalibacterium prausnitzii*, and *Prevotella copri*, which was consistent with the previous related study [5]. For Israel and Netherlands, the HP3 comprised nearly 50% in total, and the other patterns amounted to the rest half. From the remaining pie regions, HP1 occupied more prevalence over HP2. Despite the lack of background details and the varying degrees of adherence to the diet pattern among the population, the Israel is a Mediterranean country, and the traditional Israeli diet is considered to closely fall within the Mediterranean diet (MedDiet) with various adherence degrees. A recent study showed that the MedDiet intervention in overweight and obese subjects was linked with the increased abundance of fiber-degrading *Faecalibacterium prausnitzii* [6]. In another study [7], it was discovered a stronger protective association of MedDiet to mitigate cardiometabolic disease risk with a lower abundance of *Prevotella copri*. The Netherlands cohort showed putative signatures of adherence to the Israel, but with more HP1 dominance, implying the potentially great similarity in the dietary pattern.

Relations between HPs and disease. To consider the associations between healthy patterns and

disease status, we used the RF classifier with two *Bifidobaterium* species to discriminate the HPs in the disease population (See Table S5 for details). Undoubtedly, most of the disease patients were classified into HP3, followed by HP1. Patients were hardly classified into HP2 and HP4. Among all the disease patients classified into HP1, the morbidity of CA, IGT, and UC were the highest three ones, while ACVD, SA, and CD were the lowest. The proportion of all patients classified into HP3 in the total number of patients with their disease is higher than the proportion of healthy people classified into HP3, and the proportion of ACVD, CD, and RA is even more than 90%. Among patients classified as HP4, the proportion of SA also increased.

In human studies, it was found that the relative abundance of butyric acid producing bacteria *Faecalibacterium* and *Clostridium* in the IGT patients' gut would significantly decrease [8]. In our analysis, HP2 with the lowest probability of IGT development has the highest relative abundance of *Faecalibacterium* among the four health patterns, while HP3 with the closest association with IGT has the lowest relative abundance of *Clostridium*, which is consistent with the previous research. Moreover, the occurrence of IGT is considered to be related to biotin metabolism enrichment. Similarly, our metabolic subsystem statistics also showed the same result that the biotin degradation ability of HP3 was stronger than that of HP1 and HP4 (Fig. 4) [8]. On the other way, IGT is considered as the early stage of T2D. In the long term, supplementing the beneficial substrate to produce butyrate by the gut microbiota of patients with pre-T2D may be a targeted therapy to prevent or delay the progression of T2D. Previous research used first-degree relatives as comparators for RA patients and showed an abundance of *Eggerthella lenta* in patients as compared with relatives [9]. In our study, we found the similar result that the abundance of *Eggerthella lenta* in HP3 is the highest of four health patterns, but almost absent in HP2 and HP4. The obesity can alter the composition and

structure of the gut microbiota was demonstrated by previous study. The diversity of microbes in the gut of most obese patients is considered lower than the healthy people [10]. HP3 had the lowest microbial diversity among the four health patterns, and the proportion of obese patients classified as HP3 was also the highest.

## Supplementary Note 2. The impact of different transformation methods on sparse data processing on model results

To evaluate the impact of different transformation methods on model results for sparse data, we applied four types of transformations - logarithmic and square root transformation, logarithmic transformation, centered log ratio transformation, and minimum abundance threshold transformation - to our discovery and validation datasets. Specifically, we performed a total of 12 transformations, including (1) only logarithmic and square root transformations; logarithmic and square root transformation applied after two different minimum abundance threshold transformations, (2) only logarithmic transformation; logarithmic transformation applied after two minimum abundance threshold transformations, (3) only centered log ratio transformation; centered log ratio transformation applied after two minimum abundance threshold transformations, and (4) only untransformed data; untransformed data after two minimum abundance threshold transformations.

Based on our observations, we found that the balance accuracy of the models for the first three transformation types differed slightly, but all performed better than the untransformed data. In terms of individual data accuracy, the models using data transformed by the first transformation type showed the most balanced and consistently high accuracy. Furthermore, we observed that there was no significant difference in model results between data transformed by the minimum abundance

threshold transformation and untransformed data, indicating that the minimum abundance threshold transformation may have little impact on our model results. All results are shown in **Table S6-17**.

## Supplementary Note 3. Achieving a balance between predictive performance and model simplicity by selecting 92 features through the KS test

KS test was used to select features from the gut microbiota data. Then, the KS statistic for each feature between the healthy and disease groups, and ranked the features based on the p-values were calculated. Subsequently, we selected the top 92 features with balanced performance. To ensure consistency with the previous statement, we assessed the performance of hiPCA with 110 features and 92 features, while considering different transformations such as CLR, and evaluated the results based on AUC values, as shown in Figure S2. Our findings indicate that the performance of our algorithm is comparable using either set of features, as there was only a slight difference in the AUC values. According to Occam's Razor "Entities should not be multiplied unnecessarily", we believe that selecting the top 92 features using the KS test strikes a good balance between dimensionality reduction and prediction performance, and provides a more parsimonious model with comparable accuracy to using 110 features.

## Supplementary Note 4. Supplementary analysis of individualized inference revealing disease-specific microbial responses

As previously mentioned, the following both PR-50 and KS-92 contribution plots will be used for a comprehensive analysis of the remaining eight diseases in the discovery cohort. The overlay bar

graph over microbial features regarding health and eight diseases are shown in Supplementary Figure S3-10.

Atherosclerotic Cardiovascular Disease (ACVD) and Symptomatic Atherosclerosis (SA). Notably, the top species with large contributions driven by PR-50 to the hiPCA index in ACVD were *Atopobium parvulum*, *Gemella morbillorum*, *Lactobacillus salivarius*, *Fusobacterium nucleatum*, *Peptostreptococcus stomatis*, *Solobacterium moorei*. The KS-92 driven model further added *Oribacterium sinus*, *Fusobacterium varium*, *Scardovia wiggsiae*, *Streptococcus cristatus*, and *Streptococcus tigurinus*. Our unsupervised diagnosis results were mostly consistent with [11], where they used classifiers and found that most of the microorganisms presented in the oral cavity or oropharyngeal area but were reported higher in patients with ACVD. Another species *candidate division TM7 single-cell isolate TM7c* also showed a large contribution, which belonged to *Saccharibacteria* (TM7) bacteria and also highly related to the oral cavity [12, 13]. There was substantial evidence that the oral cavity bacteria could affect the development of ACVD [14]. Apart from the oral cavity, the gut microbiota could also influence the development of ACVD, including *Subdoligranulum sp.*, *Blautia producta*, *Granulicatella adiacens*, *Lachnospiraceae bacterium*, *Clostridium spp.*, and *Streptococcus spp.* Among them, *Subdoligranulum sp.* was thought to be associated with *Akkermansia muciniphila* and showed positive correlations with healthy status indicators like HDLC and microbial richness [15]. *G. adiacens* was reported to cause bacteremia and endocarditis [16]. *O. sinus* was positively correlated with blood pressure in ACVD study [11]. *F. varium* repressed the Reg3 antimicrobial family, which were important players in maintaining barrier integrity [17]. *Lachnospiraceae bacterium* was related to the ACVD-depleted butyrate-producing bacteria [11]. Lastly, for the variety of *Streptococcus spp.*, our result was in line with [18], where they

also reported a high level abundance in ACVD cases. In general, our findings indicated hiPCA was a promising inferential engine for ACVD diagnosis. For the SA cohort, the hiPCA cannot easily detect case samples. However, our diagnostic plot still revealed several key biomarkers, some top ranked species were shared with ACVD, such as *L. salivarius, S. tigurinus,* and *S. wiggsiae*. To this view, we could speculate that both oral and gut microbiota contribute heavily to the health index of atherosclerosis, which acted as a strong quantitative evidence to the previous hypothesis that both the oral and gut microbiota contribute to atherosclerosis in humans [19].

Rheumatoid arthritis (RA). RA is a complex, polygenic, and autoimmune disorder [20]. From the combined analysis of contribution plots PR-50 and KS-92, we could find that oral and gut microbial like *Atopobium parvulum, Eubacterium infirmum, Granulicatella adiacens, Lactobacillus salivarius, Scardovia wiggsiae, Streptococcus cristatus* showed top contributions to the PCA healthy indexes in RA populations, and the other microbial like *Bifidobacterium dentium, Clostridium spp.* and *Lachnospiraceae bacterium* added as high contributors, manifesting the oral-gut microbiome axis was highly associated with the etiology of RA [21-24]. Early review summarized patients with RA were enriched with *Atopobium, and Lactobacillus salivarius,* and decreased with *Streptococcus* [25]. Intriguingly, the *Subdoligranulum* sp and *Subdoligranulum variabile* invariably show the difference between RA and healthy controls, which conforms to another comparative study of immune-mediated inflammatory diseases [26]. However, our plot has not included *Porphyromonas gingivalis*, which is regarded as a keystone pathogen and has a relatively clear mechanism in RA development [21]. In general, our hiPCA findings were largely in line with the previous study, and more importantly, we further revealed a comprehensive set of pathogenic microbiomes among the RA population compared to healthy ones. Our results further demonstrated from the health inference that oral and gut

microbiomes were perturbed and contributed to the progression in RA [27].

Type 2 diabetes (T2D) and impaired glucose intolerance (IGT). T2D is a metabolic disorder, and IGT cases are usually clinically regarded as prediabetes. Substantial studies have associated microbiomes with the pathophysiology of T2D. According to PR-50 and KS-92, almost all species were found in significantly different abundance levels in patients with T2D compared with healthy subjects, such result is consistent with a previous study that T2D is not linked to any one specific species or genus [28]. To name a few, those leading contributors were *Fusobacterium varium*, *Megasphaera micronuciformis*, *Blautia producta*, *Lactobacillus salivarius*, *Solobacterium moorei*, *Clostridium ramosum*, *Gemella morbillorum*, *Clostridium ramosum*, *Peptostreptococcus stomatis*, *Lachnospiraceae bacterium*, *Clostridium clostridioforme*, *Subdoligranulum sp*, *Streptococcus gordonii*, etc. Among them, we could judge that the *Blautia* and *Lactobacillus* species were widely reported to be associated with T2D [29]. The study by [30] claimed that *S. moorei* was prone to be found in the compromised immunity across all genders and age groups. T2D subjects upon metformin were found to have higher levels of SCFA-producing bacteria *Megasphaera* than nondiabetic individuals [31]. In a recent study [32], patients with T2D were reported to have a varied abundance of *Lachnospiraceae* and a decreased abundance of *Subdoligranulum*. *Clostridium* species, like *C. ramosum*, *C. hathewayi*, and *C. clostridioforme*, may all cause bacteraemia and intra-abdominal infections [33, 34]. However, the key mechanistic resolution still remains unclear whether these bacteria synergistically or asynchronously play a causal role in T2D [35]. Lastly, by inspecting the IGT contribution plots, we can find that IGT shared most of the high contributors with T2D, like *S. moorei*, *B. producta*, several *Streptococcus* species, *Clostridium* species, and *Lachnospiraceae* species, implying the consistent etiology progress and mechanisms. Concomitantly, the KS-92 chart further

pointed out that the TOP 2 species *F. varium* and *M. micronuciformis* in T2D made relatively smaller contributions in IGT. We could also find here that the *K. pneumoniae* was not an active contributor in IGT as in T2D, manifesting that the increased dysregulated immune responses and insulin resistance from IGT to T2D could also result in the increased level of susceptibility to infections [36].

Obesity (OB), Overweight (OW), and underweight (UW). Overweight is defined by BMI greater than or equal to 25, while obesity is greater than 30. As a chronic disease, obesity is associated with various diseases, including cardiovascular diseases, and type 2 diabetes mellitus (T2DM). The human gut microbiota is always regarded to play a critical role right through the adiposity and glucose metabolism. With the contribution analysis, one can infer that there were almost identical contribution spectrums among obesity and overweight populations. The top health contribution panel was implicated by multiple *Lachnospiraceae bacterium*, *Gemella morbillorum*, *Peptostreptococcus stomatis*, *Granulicatella adiacens*, *Oribacterium sinus*, *Megasphaera micronuciformis*, *Streptococcus cristatus*, *Streptococcus tigurinus*, *Solobacterium moorei*, and *Atopobium parvulum*. Despite as the main producers of SCFAs, the *Lachnospiraceae* family was highly associated with dietary pattern [37] and obesity [38-40], also closely related to the intergenerational transmission of obesity or overweight (OBOW) [41]. The *Clostridium* genus like *C. ramosum* and *C. clostridioforme* also leveraged a large contribution to the health index. Previous investigations showed that *C. ramosum* could promote high-fat diet-induced obesity in gnotobiotic mice models [42]. In a Danish cohort study, *C. clostridioforme* was associated with the low community diversity that is more frequently obese [43], and then found in a randomized controlled trial in a Canadian children study with OBOW subjects to decrease significantly after the prebiotic intervention [44]. Apart from that, analysis of the hiPCA contribution chart further indicated that the oral bacteria contribute a large proportion to the

development of obesity. For example, *Megasphaera* was among the key oral bacterial taxa associated with OBOW and *Oribacterium* was another key oral microbe that may be co-occurred with tryptophan biosynthesis [45]. In a MedDiet Intervention study in OWOB subjects, *S. cristatus* was observed with increased levels and was regarded to inhibit virulence gene expression in periodontopathogenic bacteria *P. gingivalis* [46]. Generally, to explain the possible mechanism of oral bacteria to obesity, early researchers made three hypotheses, namely through increasing metabolic efficiency, increasing appetite, and redirecting energy metabolism [47]. Both oral and gut bacteria contributed to obesity, but the cross talks between oral microbiota with gut microbiota in obesity development still need further endeavors [48, 49]. Lastly, we consider underweight, which is defined by BMI less than the cut-off point of 18.5. It is usually related to malnutrition caused by food shortage or disease. Compared with obesity, the relationship between underweight and gut microbiota is underexplored [50]. Despite the appeared dissimilarity in definition and etiology, this study observed great overlap in the considered microbiota contribution spectrum among the underweight and overweight/obesity groups. Interestingly, our result may conform to a recent study in which they found no statistically significant differences among the underweight, normal weight, and overweight individuals at the genus level [50].

## Supplementary Note 5. hiPCA is largely reproducible on the additional validation data

To evaluate the generalization capability of hiPCA, the independent validation data consisting of 782 samples (118 healthy individuals and 664 unhealthy individuals) will be tested, which include ankylosing spondylitis (AS), colorectal adenoma (CA), colorectal cancer (CRC), CD, liver cirrhosis

(LC), non-alcoholic fatty liver disease (NAFLD), RA, UW, OW, and OB.

As shown in Figure S11. Our hiPCA with KS-92 signal panel has a balanced accuracy of 70.26%, and the detection accuracy for the healthy and unhealthy group were 70.34% and 70.18%, respectively. Notably, the results achieved almost identical performance as on the discovery data (71.40% in healthy group and 71.24% in unhealthy group). To make a close investigation, we separately considered all sub-cohorts according to the published studies. The accuracies by hiPCA (KS-92) on three healthy cohorts were 46.43% (H1), 84.48% (H2), and 65.63% (H3), which implied large variations among healthy group background in three studies. As the matter of fact, through inspecting the original studies, we found that only the H2 cohort declared that the control individuals were not suffering from any diseases. H1 and H3 only ensured that the controls were not Parkinson's disease patients (H1) or not colorectal cancer patients (H3), and they could not exclude other health problems in the control group. In the meantime, we also found that the average ages in these healthy sub-groups were 65.6 (H1), 31.9 (H2), and 57.3 (H3) respectively, which further demonstrated potential accelerated age-related health loss in the elderly. In this regard, the hiPCA could also serve as an additional indicator for the healthy people's initial assessment during recruitment. For those unhealthy phenotype sub-cohorts, we had the following: 85.98% (141 of 164) for LC, 81.4% (70 of 86) for NAFLD, 66.67% (10 of 15) for CD, 66.67% (18 of 27) for CA, 63.27% (31 of 49) for RA, 56.7% (55 of 97) for AS, 50% (7 of 14) for OB, 47.54% (29 of 61) for OW, 14.29% (4 of 28) for UW, and 92.5% (37 of 40), 78.69% (48 of 61), and 72.73% (16 of 22) for three different cohorts of CRC. As can be seen, our hiPCA provided well adverse health predictions (>75%) for LC, CRC, and NAFLD, where GMHI could only perform well on LC and NAFLD. Comparing with GMHI, we could conclude hiPCA is a more accurate and robust index for nonhealthy prediction (70.18% vs. 62.2%), and that

KS-92 is a more robust, generalized microbiome signal panel that can perform well even across different diseases.

Like the discovery set, inferential analysis was made by bacteria-to-health-index contribution analysis, and the plots were shown in Figure S12-21. CRC and CA: Our results showed that the validation set highly reproduced the results of the discovery set, with only a slight difference in the ranking of contributions of some species. By exploring the hiPCA performance for CRC diagnosis, those marker species contribute most on average to the health index in PR-50, which could be listed as *Peptostreptococcus stomatis*, *Gemella morbillorum*, *Granulicatella adiacens, Fusobacterium nucleatum*, *Atopobium parvulum, Gemella sanguinis,* and *Clostridium clostridioforme*. KS-92 shared most contributors, but also further supplements with *Streptococcus cristatus*, *Parvimonas micra, Streptococcus tigurinus,* and *Eubacterium infirmum*. Among them, *Peptostreptococcus stomatis, Fusobacterium nucleatum,* and *Parvimonas micra* have been well described before. Apart from that, other species, such as *B. dentium*, *S. bovis*, *S. gallolyticus*, *S. Tigurinus*, and *Gemella morbillorum* also need to be analyzed further. Previous studies showed that *Bifidobacterium* are enriched in people without CA, but our results suggests that *B. dentium* has a great contribution to CA, which is contrary to the current research. *B. dentium* is capable of enhancing the intestinal mucus layer and goblet cell function via upregulation of gene expression and autophagy signaling pathways, with a net increase in mucin production, and then provide a protective effect in the colitis model [51, 52]. However, a study reported that the abundance of *Bifidobacterium* was increased in the benign group [53]. The effect and mechanism of *B. dentium* on CA require further discussion. A variety of microbiota in *Streptococcus* are thought to be associated with CRC and CA, including *S. bovis* and *S. gallolyticus* [54]. However, this association is still under controversy. Some data do not support the idea that *S. bovis* colonizes

CRC and CA [55]. *S. gallolyticus* (previously known as S. bovis biotype I) is thought to be more closely related to CRC and CA [56]. *S. Tigurinus* is suspected of causing bacteremia due to its translocation. This species has recently been reported to be extremely pathogenic [57]. In another study for predicting colon cancer, *Gemella morbillorum* was used as a biomarker of fecal bacteria, and the predictive model with good sensitivity and specificity was established as a new diagnostic tool for CRC [58]. *Gemella morbillorum* was shown to be enriched in the gut of patients with CRC by metagenomics [59], and positively correlated with the enrichment of amino acids, cadaverine, and creatine in CRC [60].

AS and RA: Through the analysis of contribution plots PR-50 and KS-92, we can clearly find that AS and RA share many high-contribution species, although these high-contribution species have differences in disease contribution. For AS, the contribution plot PR-50 reveals highly contributing species, including *Clostridium clostridioforme, Clostridium symbiosum, Blautia producta, Flavonifractor plautii*, *Clostridium asparagiforme*, *Anaerotruncus colihominis*, *Clostridium citroniae, Clostridiales bacterium 1_7_47FAA*, *Clostridium hathewayi*, *Streptococcus mitis 11/5*, *Clostridium ramosum*, *Lactobacillus salivarius*, *Lachnospiraceae bacterium 1_4_56FAA*, *Bifidobacterium dentium*, *Clostridium bolteae.* The contribution plot KS-92 further reveals high-contribution species such as *Megasphaera micronuciformis*, *Lactobacillus mucosae*, *Streptococcus cristatus*, *Rothia mucilaginosa, and Fusobacterium ulcerans*. Yin found that *Clostridium 1_7_47FAA*, *Clostridium bolteae, and Clostridium hatheway* were enriched in patients with AS through statistical analysis [61]. In previous studies, *Streptococcus* and *Lactobacillus* were confirmed to be enriched in patients with AS [62]. *Bifidobacterium* is usually used in probiotics, and accumulated in patients with AS [63]. For the RA cohort, the performance of the diagnostic plot is affected due to the impact of the batch effect,

resulting in a slight difference in high-contribution species between the RA of the validation set and the RA of the discovery set. However, through the contribution plot, we can still find many common high-contribution species, including *Clostridium asparagiforme*, *Clostridium ramosum*, *Clostridium clostridioform*, *Clostridium citroniae*, *Clostridium sympiosum*, *Clostridiales bacterium 1_7_47FAA*, and *Lachnospiraceae bacterium*. *Clostridium asparagiforme* and *Lachnospiraceae bacterium* were enriched in patients with RA compared to healthy control groups [64]. This is consistent with the above analysis results.

CD: In the analysis of validation set, we only discuss CD, nor UC. Although the exact cause is unknown, CD appears to be due to a combination of environmental factors and genetic predisposition. In this paper, the most genus in the discovery set and the validation set are similar. However, compared with the other diseases we analyzed, the contribution degree of bacteria in the discovery and validation sets to CD was different. For KS-92, eight of the 12 species with the highest contribution were consistent. The difference in contribution between the discovery set and validation set is mainly reflected in *Fusobacterium ulcerans*, *Lachnospiraceae bacterium 6_1_63FAA*, *Fusobacterium mortiferum*, and *Clostridium scindens* of KS-92, *Granulicatella adiacens* of PR-50. This difference may be due to batch effects and difficulty in diagnosing CD. The diagnosis of CD can sometimes be challenging and often requires extensive testing to help doctors make the diagnosis. CD may not be diagnosed with complete certainty, even after a battery of tests. In the validation set, the degree of contribution of most microbiota was similar in KS-92 and PR-50. High contribution candidates in both PR-50 and KS-92 include *Peptostreptococcus stomatis*, *Fusobacterium nucleatum*, and *Clostridium clostridioforme*, while the degree of contribution of *Flavonifractor plautii*, *Gemella morbillorum* is different between PR-50 and KS-92. There are some species have contributed greatly

to the diagnosis of diseases, but have not been well described in the previous content. Fusobacterium species have been implicated recently in a broad spectrum of human pathologies, including Crohn's disease, ulcerative colitis, and colorectal cancer [65, 66]. *F. nucleatum* targeted caspase activation and recruitment domain 3 (CARD3) to activate the endoplasmic reticulum stress pathway and promote *F. nucleatum*-mediated mucosal barrier damage *in vivo* and *in vitro* during CD development [67]. Moreover, previous studies demonstrated that highly invasive *F. nucleatum* isolates derived from the inflamed guts of patients with CD evoked significantly greater MUC2 and tumor necrosis factor alpha (TNF-α) gene expression than minimally invasive strains isolated from the noninflamed gut in human colonic epithelial cells and in a rat ligated colonic loop model of infection [68]. In addition, *F. nucleatum* accelerates the progression of colitis-associated colorectal cancer by promoting epithelial-mesenchymal transition [69]. For *Clostridium*, *C. clostridioforme*, *C. scindens*, *C. ramosum, C. symbiosum, C. citroniae,* and *C. asparagiforme* high contribution candidates in CD. Most of them are commonly not dominant in the fecal microflora of healthy subjects [70]. *C. scindens* has been previously associated with a case of an adolescent female with CD [71]. AS and CD are likely caused by subclinical bowel infections with *Klebsiella* [72, 73]. This trend is also predicted in the results of our validation set.

LC and NAFLD: NAFLD is a class of metabolic disorders that can progress from fatty liver to steatohepatitis, further leading to cirrhosis [74]. According to the contribution plot PR-50, the main contributing species of LC is *Fusobacterium nucleatum, Lactobacillus salivarius, Streptococcus gordonii, Granulicatella adiacens, Atopobium parvulum, Peptostreptococcus stomatis, Lachnospiraceae bacterium 1_4_56FAA, Streptococcus sanguinis, Clostridium symbiosum, Clostridiales bacterium 1_7_47FAA, Streptococcus mitis 11/5, Lachnospiraceae bacterium*

*9_1_43BFAA*, *Clostridium ramosum*. The contribution plot KS-92 further reveals the high-contributing species of LC, *Megasphaera micronuciformis*, *Streptococcus cristatus*, *Streptococcus tigurinus*, *Fusobacterium varium* were added to high-contribution species. Previous studies have identified increased abundances of the oral microbe *Fusobacterium* in the setting of LC [75]. Our diagnosis is consistent with previous studies, where they used the Wilcoxon rank-sum test in the healthy control group and the cirrhosis group and found that *Fusobacteria*, *Streptococcus*, and *Clostridium* were enriched in the cirrhosis group [76]. *Streptococcus* are taxa that are part of the oral bacterial ecosystem, while *Streptococcus* appeared in previous studies to distinguish between patients with LC and healthy individuals, which may mean that the development of LC may have occurred from the oral cavity, consistent with previous views [76-78]. For the NAFLD cohort, high-contribution species mainly include *Atopobium parvulum*, *Streptococcus tigurinus*, *Scardovia wiggsiae*, *Lachnospiraceae bacterium 4_1_37FAA*, *Lachnospiraceae bacterium 6_1_63FAA*, *Blautia producta*, *Megasphaera micronuciformis*, *Clostridium ramosum, and Streptococcus cristatus*. We can clearly find that the high-contribution species of NAFLD and LC are mostly the same, although their variation in NAFLD is not as significant as in LC, which implies the close relationship between NAFLD and LC [79]. Our results are consistent with previous studies that *Clostridium bolteae*, *Clostridium nexills*, *Ruminococcus gnavusb, and Atopobium parvulum* are associated with blood biomarkers of liver disease [80]. *Streptococcus* is a relatively important contributor genus in both NAFLD and LC, and the variation of *M. micronuciformis* in LC is much higher than in NAFLD. This is consistent with previous studies, which found that *Megasphaera* was only enriched in people with NAFLD-cirrhosis, and *Streptococcus* was enriched in NAFLD-cirrhosis and NAFLD, which reflected the differences between LC and NAFLD [81].

OB/OW/UW: The contribution of different strains to OB, OW, and UW was highly similar in the discovery set, but this rule seemed to be weakened in the validation set. Most of the bacteria with a high contribution in OB and OW are still the same, including *Gemella haemolysans, Solobacterium moorei, Atopobium parvulum, Clostridium asparagus, Gamella Sanguinis, Granulicatella Adiacens*. However, only a few bacteria of OW and UW had high contribution and were similar, namely *Ruminococcus torques* and *Megasphaera micronuciformis*. *C. ramosum, C. clostridioforme, Megasphaera, Oribacterium, S. cristatus*, and *P. gingivalis* have been described in detail above, so we will not repeat them in the validation set. *R. torques* appeared in three disease states simultaneously with a high contribution. It is the most abundant intestinal bacteria in obese people, and has been investigated to be linked to obesity [82]. A meta-transcriptomic analysis revealed *R. torques* as a bacterium known to reduce intestinal barrier integrity [83]. In three studies of intervention improvement in experimental animals fed a high-fat diet, the results showed that the relative abundance of *R. torques* in the intestines of the animals was reduced, and the inflammation of the animals was improved [84-86]. However, the relationship between *R. torques* and UW has not been proved well. *Veillonella* are a group of bacteria that can't ferment in the gut, and they cannot use carbohydrates or amino acids. In our validation set, it has a high contribution to UW, but there are few studies on *Veillonella* and UW at present.

## Supplementary Note 6. The results further demonstrate strong reproducibility of hiPCA on the test data.

In order to further evaluate the generalization ability of hiPCA, a separate test dataset consisting of 605 samples (292 healthy individuals and 313 unhealthy individuals) was used, which included

End-stage renal disease (ESRD) and Schizophrenia (SCZ). As shown in Figure S22, our KS-92 signal panel hiPCA achieved a balanced accuracy of 71.60%, with detection accuracies of 67.81% and 75.40% for the healthy and unhealthy groups, respectively. Further analysis of the unhealthy phenotype subgroups revealed that ESRD had a good adverse health prediction (85.20% in 190 out of 223 individuals), while SCZ had a detection rate of 51.11% (46 out of 90 individuals). Overall, it can be concluded that hiPCA demonstrates strong reproducibility on the test data and provides good predictions for adverse health outcomes, particularly for ESRD.

Like the discovery and validation set, inferential analysis was made by bacteria-to-health-index contribution analysis, and the plots were shown in Figure S23-24.

ESRD: In test cohort, *Gemella morbillorum*, *Granulicatella adiacens*, *Lachnospiraceae bacterium 9_1_43BFAA*, *Clostridium clostridioforme*, *Gemella sanguinis*, *Clostridium ramosum*, and *Lactobacillus salivarius* were the highest contribution biomarkers in ESRD with PR-50 features. KS-92 shared most contributors but was further supplemented with *Streptococcus cristatus* and *Scardovia wiggsiae*. Gut microbiota can be a potential therapeutic target for kidney diseases, such as chronic kidney disease (CKD), ESRD, peritoneal dialysis, hemodialysis and renal transplantation. Among them, the diversity of *Scardovia wiggsiae* has been reported to be associated with indoxyl sulfate, which is an inflammatory biomarker of ESRD [87]. A previous study found that the absolute quantity of total bacteria and *Clostridium* were signifcantly reduced in ESRD patients [88]. In our study, similarly, *Clostridium ramosum* and *Clostridium clostridioforme* were the important contributors in ESRD. However, there are still many strains that have not been reported, and it seems that we can refer to the degree of contribution to explore more potential diagnostic or therapeutic targets.

SCZ: In the test cohort, *Lactobacillus salivarius*, *Anaerotruncus colihominis*, *Clostridium clostridioforme*, *Atopobium parvulum*, *Ruminococcus gnavus*, *Lachnospiraceae bacterium 1_4_56FAA*, and *Clostridium symbiosum* are the highest contribution biomarkers in SCZ with PR-50 features. KS-92 indicated that *Megasphaera micronuciformis*, *Lactobacillus mucosae*, *Lactobacillus salivaryus*, *Fusobacterium varium*, *Atopobium parvulum*, *Lachnospiraceae bacterium 9_1_43BFAA* and *Anaerotruncus colihominis* were the top seven contributing species to SCZ. Among them, *Lactobacillus salivarius*, *Anaerotruncus colihominis*, and *Atopobium parvulum* were the main contributors shared by PR-50 and KS-92. Previous studies have reported that *Lachnospiraceae* and *Ruminococcaceae* were associated with SCZ [89], which is similar to our results. Gut microbiota disturbances in patients with depression, bipolar disorder, schizophrenia, and anxiety all tend to one pattern [90]. *Lactobacillus salivarius* as a probiotic seemed to have positive effects on the mild chronic inflammation processes in major depressive disorder (MDD) [91]. In future work, we can mine more information on other potential target strains and their intervention effects.

# References


1. Arumugam M, Raes J, Pelletier E, Le Paslier D, Yamada T, Mende DR, Fernandes GR, Tap J, Bruls T, Batto J-M: **Enterotypes of the human gut microbiome**. *nature* 2011, **473**(7346):174-180.
2. Costea PI, Hildebrand F, Arumugam M, Bäckhed F, Blaser MJ, Bushman FD, De Vos WM, Ehrlich SD, Fraser CM, Hattori M: **Enterotypes in the landscape of gut microbial community composition**. *Nature microbiology* 2018, **3**(1):8-16.
3. Gorvitovskaia A, Holmes SP, Huse SM: **Interpreting Prevotella and Bacteroides as biomarkers of diet and lifestyle**. *Microbiome* 2016, **4**(1):1-12.
4. Mobeen F, Sharma V, Tulika P: **Enterotype variations of the healthy human gut microbiome in different geographical regions**. *Bioinformation* 2018, **14**(9):560.
5. Liu W, Zhang J, Wu C, Cai S, Huang W, Chen J, Xi X, Liang Z, Hou Q, Zhou B: **Unique features of ethnic Mongolian gut microbiome revealed by metagenomic analysis**. *Scientific reports* 2016, **6**(1):1-13.
6. Meslier V, Laiola M, Roager HM, De Filippis F, Roume H, Quinquis B, Giacco R, Mennella I, Ferracane R, Pons N: **Mediterranean diet intervention in overweight and obese subjects lowers plasma cholesterol and causes changes in the gut microbiome and metabolome independently of energy intake**. *Gut* 2020, **69**(7):1258-1268.
7. Wang DD, Nguyen LH, Li Y, Yan Y, Ma W, Rinott E, Ivey KL, Shai I, Willett WC, Hu FB: **The gut microbiome modulates the protective association between a Mediterranean diet and cardiometabolic disease risk**. *Nature medicine* 2021, **27**(2):333-343.
8. Wu H, Tremaroli V, Schmidt C, Lundqvist A, Olsson LM, Kramer M, Gummesson A, Perkins R, Bergstrom G, Backhed F: **The Gut Microbiota in Prediabetes and Diabetes: A Population-Based Cross-Sectional Study**. *Cell Metabolism* 2020, **32**(3):379-390.e373.
9. Bodkhe R, Balakrishnan B, Taneja V: **The role of microbiome in rheumatoid arthritis treatment**. *Therapeutic advances in musculoskeletal disease* 2019, **11**:1759720x19844632.
10. Thingholm LB, Rühlemann MC, Koch M, Fuqua B, Laucke G, Boehm R, Bang C, Franzosa EA, Hübenthal M, Rahnavard A *et al*: **Obese Individuals with and without Type 2 Diabetes Show Different Gut Microbial Functional Capacity and Composition**. *Cell host & microbe* 2019, **26**(2):252-264.e210.
11. Jie Z, Xia H, Zhong S-L, Feng Q, Li S, Liang S, Zhong H, Liu Z, Gao Y, Zhao H: **The gut microbiome in atherosclerotic cardiovascular disease**. *Nature communications* 2017, **8**(1):1-12.
12. Baker JL, Morton JT, Dinis M, Alvarez R, Tran NC, Knight R, Edlund A: **Deep metagenomics examines the oral microbiome during dental caries, revealing novel taxa and co-occurrences with host molecules**. *Genome research* 2021, **31**(1):64-74.

13. Murugkar PP, Collins AJ, Chen T, Dewhirst FE: **Isolation and cultivation of candidate phyla radiation Saccharibacteria (TM7) bacteria in coculture with bacterial hosts**. *Journal of oral microbiology* 2020, **12**(1):1814666.

14. Jonsson AL, Bäckhed F: **Role of gut microbiota in atherosclerosis**. *Nature Reviews Cardiology* 2017, **14**(2):79-87.

15. Van Hul M, Le Roy T, Prifti E, Dao MC, Paquot A, Zucker J-D, Delzenne NM, Muccioli GG, Clément K, Cani PD: **From correlation to causality: the case of Subdoligranulum**. *Gut microbes* 2020, **12**(1):1849998.

16. Christensen JJ, Facklam RR: **Granulicatella and Abiotrophia species from human clinical specimens**. *Journal of Clinical Microbiology* 2001, **39**(10):3520-3523.

17. Geva-Zatorsky N, Sefik E, Kua L, Pasman L, Tan TG, Ortiz-Lopez A, Yanortsang TB, Yang L, Jupp R, Mathis D: **Mining the human gut microbiota for immunomodulatory organisms**. *Cell* 2017, **168**(5):928-943. e911.

18. Wang J, Li W, Wang C, Wang L, He T, Hu H, Song J, Cui C, Qiao J, Qing L *et al*: **Enterotype Bacteroides Is Associated with a High Risk in Patients with Diabetes: A Pilot Study**. *Journal of diabetes research* 2020, **2020**:6047145.

19. Koren O, Spor A, Felin J, Fåk F, Stombaugh J, Tremaroli V, Behre CJ, Knight R, Fagerberg B, Ley RE: **Human oral, gut, and plaque microbiota in patients with atherosclerosis**. *Proceedings of the National Academy of Sciences* 2011, **108**(Supplement 1):4592-4598.

20. Scher JU, Abramson SB: **The microbiome and rheumatoid arthritis**. *Nature reviews rheumatology* 2011, **7**(10):569-578.

21. du Teil Espina M, Gabarrini G, Harmsen HJ, Westra J, van Winkelhoff AJ, van Dijl JM: **Talk to your gut: the oral-gut microbiome axis and its immunomodulatory role in the etiology of rheumatoid arthritis**. *FEMS microbiology reviews* 2019, **43**(1):1-18.

22. Wang J, Jia H: **Metagenome-wide association studies: fine-mining the microbiome**. *Nature Reviews Microbiology* 2016, **14**(8):508-522.

23. Blod C, Schlichting N, Schülin S, Suttkus A, Peukert N, Stingu CS, Hirsch C, Elger W, Lacher M, Bühligen U: **The oral microbiome—the relevant reservoir for acute pediatric appendicitis?** *International journal of colorectal disease* 2018, **33**(2):209-218.

24. Liu X, Tong X, Zhu J, Tian L, Jie Z, Zou Y, Lin X, Liang H, Li W, Ju Y: **Metagenome-genome-wide association studies reveal human genetic impact on the oral microbiome**. *Cell discovery* 2021, **7**(1):1-16.

25. Lu M, Xuan S, Wang Z: **Oral microbiota: A new view of body health**. *Food Science and Human Wellness* 2019, **8**(1):8-15.

26. Forbes JD, Chen C-y, Knox NC, Marrie R-A, El-Gabalawy H, de Kievit T, Alfa M, Bernstein CN, Van Domselaar G: **A comparative study of the gut microbiota in immune-mediated inflammatory diseases—does a common dysbiosis exist?** *Microbiome* 2018, **6**(1):1-15.

27. Zhang X, Zhang D, Jia H, Feng Q, Wang D, Liang D, Wu X, Li J, Tang L, Li Y: **The oral and gut microbiomes are perturbed in rheumatoid arthritis and partly normalized after treatment**. *Nature medicine* 2015, **21**(8):895-905.

28. Tierney BT, Tan Y, Kostic AD, Patel CJ: **Gene-level metagenomic architectures across diseases yield high-resolution microbiome diagnostic indicators**. *Nature communications* 2021, **12**(1):1-12.

29. Gurung M, Li Z, You H, Rodrigues R, Jump DB, Morgun A, Shulzhenko N: **Role of gut microbiota in type 2 diabetes pathophysiology**. *EBioMedicine* 2020, **51**:102590.

30. Barrak I, Stájer A, Gajdács M, Urbán E: **Small, but smelly: the importance of Solobacterium moorei in halitosis and other human infections**. *Heliyon* 2020, **6**(10):e05371.

31. Aron-Wisnewsky J, Warmbrunn MV, Nieuwdorp M, Clément K: **Metabolism and metabolic disorders and the microbiome: The intestinal microbiota associated with obesity, lipid metabolism, and metabolic health—Pathophysiology and therapeutic strategies**. *Gastroenterology* 2021, **160**(2):573-599.

32. Alvarez-Silva C, Kashani A, Hansen TH, Pinna NK, Anjana RM, Dutta A, Saxena S, Støy J, Kampmann U, Nielsen T: **Trans-ethnic gut microbiota signatures of type 2 diabetes in Denmark and India**. *Genome medicine* 2021, **13**(1):1-13.

33. Qin J, Li Y, Cai Z, Li S, Zhu J, Zhang F, Liang S, Zhang W, Guan Y, Shen D: **A metagenome-wide association study of gut microbiota in type 2 diabetes**. *Nature* 2012, **490**(7418):55-60.

34. Karlsson FH, Tremaroli V, Nookaew I, Bergström G, Behre CJ, Fagerberg B, Nielsen J, Bäckhed F: **Gut metagenome in European women with normal, impaired and diabetic glucose control**. *Nature* 2013, **498**(7452):99-103.

35. Upadhyaya S, Banerjee G: **Type 2 diabetes and gut microbiome: at the intersection of known and unknown**. *Gut microbes* 2015, **6**(2):85-92.

36. Jin L, Liu Y, Jing C, Wang R, Wang Q, Wang H: **Neutrophil extracellular traps (NETs)-mediated killing of carbapenem-resistant hypervirulent Klebsiella pneumoniae (CR-hvKP) are impaired in patients with diabetes mellitus**. *Virulence* 2020, **11**(1):1122-1130.

37. Salonen A, Lahti L, Salojärvi J, Holtrop G, Korpela K, Duncan SH, Farquharson F, Johnstone AM, Lobley GE, Louis P: **Impact of diet and individual variation on intestinal microbiota composition and fermentation products in obese men**. *The ISME journal* 2014, **8**(11):2218-2230.

38. Perry RJ, Peng L, Barry NA, Cline GW, Zhang D, Cardone RL, Petersen KF, Kibbey RG, Goodman AL, Shulman GI: **Acetate mediates a microbiome–brain–β-cell axis to promote metabolic syndrome**. *Nature* 2016, **534**(7606):213-217.

39. Vacca M, Celano G, Calabrese FM, Portincasa P, Gobbetti M, De Angelis M: **The**

**controversial role of human gut lachnospiraceae**. *Microorganisms* 2020, **8**(4):573.

40. Hildebrandt MA, Hoffmann C, Sherrill–Mix SA, Keilbaugh SA, Hamady M, Chen YY, Knight R, Ahima RS, Bushman F, Wu GD: **High-fat diet determines the composition of the murine gut microbiome independently of obesity**. *Gastroenterology* 2009, **137**(5):1716-1724. e1712.

41. Tun HM, Bridgman SL, Chari R, Field CJ, Guttman DS, Becker AB, Mandhane PJ, Turvey SE, Subbarao P, Sears MR: **Roles of birth mode and infant gut microbiota in intergenerational transmission of overweight and obesity from mother to offspring**. *JAMA pediatrics* 2018, **172**(4):368-377.

42. Woting A, Pfeiffer N, Loh G, Klaus S, Blaut M: **Clostridium ramosum promotes high-fat diet-induced obesity in gnotobiotic mouse models**. *MBio* 2014, **5**(5):e01530-01514.

43. Le Chatelier E, Nielsen T, Qin J, Prifti E, Hildebrand F, Falony G, Almeida M, Arumugam M, Batto J-M, Kennedy S: **Richness of human gut microbiome correlates with metabolic markers**. *Nature* 2013, **500**(7464):541-546.

44. Nicolucci AC, Hume MP, Martínez I, Mayengbam S, Walter J, Reimer RA: **Prebiotics reduce body fat and alter intestinal microbiota in children who are overweight or with obesity**. *Gastroenterology* 2017, **153**(3):711-722.

45. Aleti G, Kohn JN, Troyer EA, Weldon K, Huang S, Tripathi A, Dorrestein PC, Swafford AD, Knight R, Hong S: **Salivary bacterial signatures in depression-obesity comorbidity are associated with neurotransmitters and neuroactive dipeptides**. *BMC microbiology* 2022, **22**(1):1-17.

46. Laiola M, De Filippis F, Vitaglione P, Ercolini D: **A mediterranean diet intervention reduces the levels of salivary periodontopathogenic bacteria in overweight and obese subjects**. *Applied and environmental microbiology* 2020, **86**(12):e00777-00720.

47. Goodson J, Groppo D, Halem S, Carpino E: **Is obesity an oral bacterial disease?** *Journal of dental research* 2009, **88**(6):519-523.

48. Benahmed AG, Gasmi A, Doşa A, Chirumbolo S, Mujawdiya PK, Aaseth J, Dadar M, Bjørklund G: **Association between the gut and oral microbiome with obesity**. *Anaerobe* 2021, **70**:102248.

49. Gupta A, Osadchiy V, Mayer EA: **Brain–gut–microbiome interactions in obesity and food addiction**. *Nature Reviews Gastroenterology & Hepatology* 2020, **17**(11):655-672.

50. Wan Y, Yuan J, Li J, Li H, Yin K, Wang F, Li D: **Overweight and underweight status are linked to specific gut microbiota and intestinal tricarboxylic acid cycle intermediates**. *Clinical Nutrition* 2020, **39**(10):3189-3198.

51. Engevik MA, Luk B, Chang-Graham AL, Hall A, Herrmann B, Ruan W, Endres BT, Shi Z, Garey KW, Hyser JM *et al*: **Bifidobacterium dentium Fortifies the Intestinal Mucus Layer via Autophagy and Calcium Signaling Pathways**. *mBio* 2019, **10**(3):e01087-01019.

52. Engevik M, Ruan W, Ihekweazu F, Versalovic J: **MODULATION OF THE MUCUS**

**LAYER BY BIFIDOBACTERIUM DENTIUM PROVIDES PROTECTION IN A MODEL OF COLITIS**. *Inflammatory Bowel Diseases* 2020, **26**:S38-S38.

53. Yu X, Zhang Y, Yu E, Wang N, Shuai Q, Yan F, Jiang L, Wang H, Liu J, Chen Y *et al*: **Changes in gut microbiota of patients with colorectal adenoma and polyps:a case control study**. *Fudan University Journal of Medical Sciences* 2018, **45**(5):658-663.

54. Abdulamir AS, Hafidh RR, Abu Bakar F: **The association of Streptococcus bovis/gallolyticus with colorectal tumors: the nature and the underlying mechanisms of its etiological role**. *Journal of experimental & clinical cancer research : CR* 2011, **30**(1):11.

55. Norfleet RG, Mitchell PD: **Streptococcus bovis does not selectively colonize colorectal cancer and polyps**. *Journal of clinical gastroenterology* 1993, **17**(1):25-28.

56. Boleij A, Tjalsma H: **The itinerary of Streptococcus gallolyticus infection in patients with colonic malignant disease**. *Lancet Infectious Diseases* 2013, **13**(8):719-724.

57. Hirai J, Sakanashi D, Hagihara M, Haranaga S, Uechi K, Kato H, Hamada H, Nishiyama N, Koizumi Y, Suematsu H *et al*: **Bacteremia due to Streptococcus tigurinus: A case report and literature review**. *Journal of Infection and Chemotherapy* 2016, **22**(11):762-766.

58. Yao Y, Ni H, Wang X, Xu Q, Zhang J, Jiang L, Wang B, Song S, Zhu X: **A New Biomarker of Fecal Bacteria for Non-Invasive Diagnosis of Colorectal Cancer**. *Frontiers in cellular and infection microbiology* 2021, **11**:744049.

59. Chang HB, Mishra R, Cen CP, Tang YQ, Ma CC, Wasti S, Wang YY, Ou QY, Chen KN, Zhang JC: **Metagenomic Analyses Expand Bacterial and Functional Profiling Biomarkers for Colorectal Cancer in a Hainan Cohort, China**. *Current Microbiology* 2021, **78**(2):705-712.

60. Avuthu N, Guda C: **Meta-Analysis of Altered Gut Microbiota Reveals Microbial and Metabolic Biomarkers for Colorectal Cancer**. *Microbiology spectrum* 2022, **10**(4):e0001322.

61. Yin J, Sternes PR, Wang M, Song J, Morrison M, Li T, Zhou L, Wu X, He F, Zhu J *et al*: **Shotgun metagenomics reveals an enrichment of potentially cross-reactive bacterial epitopes in ankylosing spondylitis patients, as well as the effects of TNFi therapy upon microbiome composition**. *Annals of the rheumatic diseases* 2020, **79**(1):132-140.

62. Wang Y, Wei J, Zhang W, Doherty M, Zhang Y, Xie H, Li W, Wang N, Lei G, Zeng C: **Gut dysbiosis in rheumatic diseases: A systematic review and meta-analysis of 92 observational studies**. *EBioMedicine* 2022, **80**:104055.

63. Wen C, Zheng Z, Shao T, Liu L, Xie Z, Le Chatelier E, He Z, Zhong W, Fan Y, Zhang L *et al*: **Quantitative metagenomics reveals unique gut microbiome biomarkers in ankylosing spondylitis**. *Genome biology* 2017, **18**(1):142.

64. Zhang X, Zhang D, Jia H, Feng Q, Wang D, Liang D, Wu X, Li J, Tang L, Li Y *et al*: **The oral and gut microbiomes are perturbed in rheumatoid arthritis and partly normalized after treatment**. *Nature medicine* 2015, **21**(8):895-905.

65. Sekizuka T, Ogasawara Y, Ohkusa T, Kuroda M: **Characterization of Fusobacterium varium Fv113-g1 isolated from a patient with ulcerative colitis based on complete genome sequence and transcriptome analysis**. *PloS one* 2017, **12**(12):e0189319.

66. Manson McGuire A, Cochrane K, Griggs AD, Haas BJ, Abeel T, Zeng Q, Nice JB, MacDonald H, Birren BW, Berger BW *et al*: **Evolution of invasion in a diverse set of Fusobacterium species**. *mBio* 2014, **5**(6):e01864.

67. Cao P, Chen Y, Guo X, Chen Y, Su W, Zhan N, Dong W: **Fusobacterium nucleatum Activates Endoplasmic Reticulum Stress to Promote Crohn's Disease Development via the Upregulation of CARD3 Expression**. *Frontiers in pharmacology* 2020, **11**:106.

68. Dharmani P, Strauss J, Ambrose C, Allen-Vercoe E, Chadee K: **Fusobacterium nucleatum Infection of Colonic Cells Stimulates MUC2 Mucin and Tumor Necrosis Factor Alpha**. *Infection and Immunity* 2011, **79**(7):2597-2607.

69. Yu MR, Kim HJ, Park HR: **Fusobacterium nucleatum Accelerates the Progression of Colitis-Associated Colorectal Cancer by Promoting EMT**. *Cancers* 2020, **12**(10):2728.

70. Mangin I, Bonnet R, Seksik P, Rigottier-Gois L, Sutren M, Bouhnik Y, Neut C, Collins MD, Colombel JF, Marteau P *et al*: **Molecular inventory of faecal microflora in patients with Crohn's disease**. *Fems Microbiology Ecology* 2004, **50**(1):25-36.

71. Elsayed S, Zhang KY: **Isolation and 16S ribosomal RNA gene sequence-based identification of Clostridium scindens from an intra-abdominal abscess**. *Anaerobe* 2006, **12**(1):13-16.

72. Rashid T, Ebringer A: **Autoimmunity in Rheumatic Diseases Is Induced by Microbial Infections via Crossreactivity or Molecular Mimicry**. *Autoimmune diseases* 2012, **2012**:539282.

73. Sung YK, Lee JK, Lee KH, Lee KT, Kang CI: **The Clinical Epidemiology and Outcomes of Bacteremic Biliary Tract Infections Caused by Antimicrobial-Resistant Pathogens**. *American Journal of Gastroenterology* 2012, **107**(3):473-483.

74. Zhang X, Coker OO, Chu ES, Fu K, Lau HCH, Wang YX, Chan AWH, Wei H, Yang X, Sung JJY *et al*: **Dietary cholesterol drives fatty liver-associated liver cancer by modulating gut microbiota and metabolites**. *Gut* 2021, **70**(4):761-774.

75. Engevik MA, Danhof HA, Ruan W, Engevik AC, Chang-Graham AL, Engevik KA, Shi Z, Zhao Y, Brand CK, Krystofiak ES *et al*: **Fusobacterium nucleatum Secretes Outer Membrane Vesicles and Promotes Intestinal Inflammation**. *mBio* 2021, **12**(2):e02706-02720.

76. Qin N, Yang F, Li A, Prifti E, Chen Y, Shao L, Guo J, Le Chatelier E, Yao J, Wu L *et al*: **Alterations of the human gut microbiome in liver cirrhosis**. *Nature* 2014, **513**(7516):59-64.

77. Chen Y, Ji F, Guo J, Shi D, Fang D, Li L: **Dysbiosis of small intestinal microbiota in liver cirrhosis and its association with etiology**. *Scientific reports* 2016, **6**:34055.

78. Aron-Wisnewsky J, Vigliotti C, Witjes J, Le P, Holleboom AG, Verheij J, Nieuwdorp M, Clément K: **Gut microbiota and human NAFLD: disentangling microbial signatures from metabolic disorders**. *Nature reviews Gastroenterology & hepatology* 2020, **17**(5):279-297.

79. Tilg H, Adolph TE, Dudek M, Knolle P: **Non-alcoholic fatty liver disease: the interplay between metabolism, microbes and immunity**. *Nature metabolism* 2021, **3**(12):1596-1607.

80. Li RJ, Jie ZY, Feng Q, Fang RL, Li F, Gao Y, Xia HH, Zhong HZ, Tong B, Madsen L *et al*: **Network of Interactions Between Gut Microbiome, Host Biomarkers, and Urine Metabolome in Carotid Atherosclerosis**. *Frontiers in cellular and infection microbiology* 2021, **11**:708088.

81. Caussy C, Tripathi A, Humphrey G, Bassirian S, Singh S, Faulkner C, Bettencourt R, Rizo E, Richards L, Xu ZZ *et al*: **A gut microbiome signature for cirrhosis due to nonalcoholic fatty liver disease**. *Nat Commun* 2019, **10**(1):1406.

82. Xie YH, Zhang CY, Wang ZH, Wei C, Liao NJ, Wen X, Niu CW, Yi L, Wang ZJ, Xi Z: **Fluorogenic Assay for Acetohydroxyacid Synthase: Design and Applications**. *Analytical Chemistry* 2019, **91**(21):13582-13590.

83. Deaver JA, Eum SY, Toborek M: **Circadian Disruption Changes Gut Microbiome Taxa and Functional Gene Composition**. *Frontiers in microbiology* 2018, **9**:737.

84. Zeng Z, Guo X, Zhang J, Yuan Q, Chen S: **Lactobacillus paracasei modulates the gut microbiota and improves inflammation in type 2 diabetic rats**. *Food & function* 2021, **12**(15):6809-6820.

85. Yue C, Li M, Li J, Han X, Zhu H, Yu G, Cheng J: **Medium-, long- and medium-chain-type structured lipids ameliorate high-fat diet-induced atherosclerosis by regulating inflammation, adipogenesis, and gut microbiota in ApoE(-/-) mice**. *Food & function* 2020, **11**(6):5142-5155.

86. Iversen KN, Dicksved J, Zoki C, Fristedt R, Pelve EA, Langton M, Landberg R: **The Effects of High Fiber Rye, Compared to Refined Wheat, on Gut Microbiota Composition, Plasma Short Chain Fatty Acids, and Implications for Weight Loss and Metabolic Risk Factors (the RyeWeight Study)**. *Nutrients* 2022, **14**(8):1669.

87. Asgharian M, Gholizadeh P, Samadi Kafil H, Ghojazadeh M, Samadi A, Soleymani J, Jouyban A, Tayebi Khosroshahi H: **Correlation of inflammatory biomarkers with the diversity of Bacteroidaceae, Bifidobacteriaceae, Prevotellaceae and Lactobacillaceae families in the intestinal microbiota of patients with end stage renal disease**. *Advances in medical sciences* 2022, **67**(2):304-310.

88. Jiang S, Xie S, Lv D, Wang P, He H, Zhang T, Zhou Y, Lin Q, Zhou H, Jiang J *et al*: **Alteration of the gut microbiota in Chinese population with chronic kidney disease**. *Scientific reports* 2017, **7**(1):2870.

89. Wang D, Russel WA, Sun Y, Belanger K, Ay A: **Machine learning and network analysis of the gut microbiome from patients with schizophrenia and non-psychiatric subject**

**controls reveal behavioral risk factors and bacterial interactions**. *Schizophrenia Research* 2022, **251**:49-58.

90. Nikolova VL, Smith MRB, Hall LJ, Cleare AJ, Stone JM, Young AH: **Perturbations in Gut Microbiota Composition in Psychiatric Disorders: A Review and Meta-analysis**. *JAMA psychiatry* 2021, **78**(12):1343-1354.

91. Reiter A, Bengesser SA, Hauschild AC, Birkl-Töglhofer AM, Fellendorf FT, Platzer M, Färber T, Seidl M, Mendel LM, Unterweger R *et al*: **Interleukin-6 Gene Expression Changes after a 4-Week Intake of a Multispecies Probiotic in Major Depressive Disorder-Preliminary Results of the PROVIT Study**. *Nutrients* 2020, **12**(9):2575.
